# CircGNB1 drives osteoarthritis pathogenesis by inducing oxidative stress in chondrocytes

**DOI:** 10.1002/ctm2.1358

**Published:** 2023-08-03

**Authors:** Yi Liang, Lifeng Shen, Weiyu Ni, Yuhong Ding, Wentao Yang, Tianyuan Gu, Chenfeng Zhang, Jasper H. N. Yik, Dominik R. Haudenschild, Shunwu Fan, Shuying Shen, Ziang Hu

**Affiliations:** ^1^ Department of Orthopedic Surgery Sir Run Run Shaw Hospital Zhejiang University School of Medicine Hangzhou China; ^2^ Key Laboratory of Musculoskeletal System Degeneration and Regeneration Translational Research of Zhejiang Province Hangzhou China; ^3^ Ellison Musculoskeletal Research Center Department of Orthopaedic Surgery University of California System Davis California USA

**Keywords:** caveolin‐1, circGNB1, osteoarthritis, oxidative stress, RNF219, senescence, ubiquitination

## Abstract

**Background:**

Circular RNAs (circRNAs) have risen to prominence as important regulators of biological processes. This study investigated whether circGNB1 functions as a competitive endogenous RNA to regulate the pathological process of oxidative stress in age‐related osteoarthritis (OA).

**Methods:**

The relationship between circGNB1 expression and oxidative stress/OA severity was determined in cartilages from OA patients at different ages. The biological roles of circGNB1 in oxidative stress and OA progression, and its downstream targets were determined using gain‐ and loss‐of‐function experiments in various biochemical assays in human chondrocytes (HCs). The in vivo effects of circGNB1 overexpression and knockdown were also determined using a destabilization of the medial meniscus (DMM) mouse model.

**Results:**

Increased circGNB1 expression was detected in HCs under oxidative and inflammatory stress and in the cartilage of older individuals. Mechanistically, circGNB1 sponged miR‐152‐3p and thus blocked its interaction with its downstream mRNA target, ring finger protein 219 (RNF219), which in turn stabilized caveolin‐1 (CAV1) by preventing its ubiquitination at the K47 residue. CircGNB1 inhibited IL‐10 signalling by antagonizing miR‐152‐3p‐mediated RNF219 and CAV1 inhibition. Consequently, circGNB1 overexpression promoted OA progression by enhancing catabolic factor expression and oxidative stress and by suppressing anabolic genes in vitro and in vivo. Furthermore, circGNB1 knockdown alleviated the severity of OA, whereas circGNB1 overexpression had the opposite effect in a DMM mouse model of OA.

**Conclusion:**

CircGNB1 regulated oxidative stress and OA progression via the miR‐152‐3p/RNF219/CAV1 axis. Modulating circGNB1 could be an effective strategy for treating OA.

## Graphical abstract

1. CircGNB1 sponges miR‐152‐3p and up‐regulates the expression of RNF219.
2. RNF219 binds to CAV1.
3. CircGNB1/miR‐152‐3p/RNF219 axis negatively regulates the degradation of CAV1 protein by inhibiting its ubiquitination.
4. CircGNB1/miR‐152‐3p/RNF219/CAV1 axis plays a significant role in OA through IL‐10 signalling pathway.

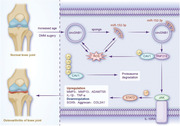

## 1 INTRODUCTION

Osteoarthritis (OA) is a usual chronic degenerative joint disease that often causes discomfort as well as a functional limitation that might have a significant impact on individuals and socioeconomic costs.^1^, ^2^ It involves pathological changes at the total knee joint level, including synovitis, subchondral bone sclerosis, and osteophyte formation; however, its predominant features and hallmarks are cartilage destruction.^3^

Chondrocytes as the primary resident cell type in articular cartilage, their main function is responsible for conserving the homeostasis of the extracellular matrix (ECM) components.^4^ Thus, the progress of OA is associated with biological factors that disrupted the well‐being of chondrocytes and lead to an abnormal state.^5^ Accumulating evidence suggests that an imbalance in the redox state, leading to oxidative stress in chondrocytes, is a key event that perturbs cartilage homeostasis during OA development.^6^, ^7^

Oxidative stress activates proinflammatory pathways^8^ and can trigger an inflammatory response^9^ and chondrocyte senescence,^10^ thereby accelerating the degradation of cartilage ECM during OA pathogenesis.^11^ Previous clinical studies have illustrated that oxidative stress, aging, and OA are interrelated.^12^, ^13^ Therefore, the inhibition of oxidative stress and reactive oxygen species (ROS) generation is considered a major factor in delaying the damage and aging of chondrocytes and preventing the pathological progression of OA.

Circular RNAs (circRNAs) are a specialized subclass of endogenous non‐coding RNAs that arise from the back splicing of exons in eukaryotic precursor mRNAs.^14^, ^15^ By reason of their covalently closed loop structure, circRNAs are resistant to RNase R and reveal higher stability than their linear counterparts.^16^ Previous studies have pointed out that circRNAs serve as microRNA (miRNA) sponges to regulate downstream target genes.^17^, ^18^ circRNAs can also act as templates for protein translation^19^ or interact with RNA‐binding proteins (RBPs) to apply their biological functions.^20^ Recently, the miRNA–mRNA crosstalk can be seen a popular topic in OA development and progression.^21^, ^22^ While the earlier studies have demonstrated that circRNAs play an vital character in regulating chondrocytes during OA pathogenesis,^23^ the mechanism of circRNAs in aging and oxidative stress‐associated OA stays elusive.

RING finger protein 219 (RNF219), one of the members in the RING finger family, comprises a conserved RING finger domain at its N‐terminal end, which is a type of ubiquitin ligase with no obvious characteristics^24^; however, there is currently limited research on RNF219, and its function in OA is still unclear. Caveolin‐1 (CAV1) as a senescence marker associated with oxidative stress in chondrocytes^25^, ^26^ has been pointed out to be associated with cartilage degeneration in OA in humans and rats.^27^ Nevertheless, the mechanisms underlying its occurrence in patients still need to be investigated.

Here, we report that circGNB1 occurs in oxidative stress‐related and age‐induced OA through the miR‐152‐3p/RNF219/CAV1 axis and IL‐10 signalling pathway.

## 2 MATERIALS AND METHODS

### 2.1 Human cartilage collection

In this study, human articular cartilage was collected from patients undergoing total knee replacement surgery according to the guidelines approved by the Ethics Committee of Sir Run Run Shaw Hospital (Zhejiang, China). All patient participants have signed a written consent form. A total of 30 knee joints were collected and separated into two groups based on the ages of participants. 50−69 years old (*n* = 15) were considered younger, while 70−85 years old (*n* = 15) were considered older. All cartilages were divided into two groups: OA lateral and OA medial. The region of interest (ROI) was defined as the tibial plateau cartilage without meniscal protection and was approximately 1‐cm wide and 2‐cm long measured along the midline of the medial or lateral tibial plateau. To assess the severity of OA in patients, the WOMAC grade and OARSI grade were used. The general conditions of patients are listed in Table S2.

### 2.2 Primary articular chondrocyte culture and treatment

Human articular chondrocytes (HCs) were extracted from the knee cartilage of patients who had total knee replacements. HCs from different types of sources were preserved separately. Articular cartilage was removed, minced, and digested with .25% trypsin‐EDTA (Sigma‐Aldrich, St. Louis, MO, USA) for 1 h at 37°C in a constant temperature shaker at 200 rpm, followed by washing with PBS and then digested with .2% type II collagenase (Sigma‐Aldrich, St. Louis, MO, USA) in an incubator/shaker at 37°C overnight. To collect chondrocytes from digest cartilage, the supernatant was filtered through a .075‐mm filter and centrifuged at 800 rpm for 5 min. Cells were then washed three times with PBS and cultured in Dulbecco's Modified Eagle Medium, supplemented with 10 % fetal bovine serum (Thermo Fisher Scientific, Waltham, MA, USA), 1% penicillin‐streptomycin, and .02% tetracycline. The culture was kept at 37°C in a humidified incubator with 5 % CO_2_. For subsequent experiments, primary chondrocytes at confluency of 70%−80% were used. Cells were passaged and transfected in the first generation. Cells were treated with IL‐1 β (R&D Systems, Minnesota, USA) to stimulate inflammation, or H_2_O_2_ (Millipore, Billerica, MA, USA) to stimulate ROS generation at various concentrations.

Details about RNA sequencing, RNA extraction, reverse transcription, qRT‐PCR, circRNA plasmid construction, mouse OA models, measurement of intracellular ROS levels, immunofluorescence, immunohistochemistry (IHC), fluorescence in situ hybridization (FISH), RNA immunoprecipitation (RIP), RNA antisense purification (RAP), knock‐down or overexpression, co‐immunoprecipitation (co‐IP), micro‐CT analysis, dual‐luciferase reporter assay, western blot, β‐galactosidase (β‐gal) staining, nuclear cytoplasmic separation experiment, histological analysis are described in Supporting Information. All primers are listed in Supplementary Table S1.

### 2.3 Statistical analysis

SPSS v22.0 was utilized to demeanor statistical analysis. Results were stated as the mean ± standard deviation (SD). Statistical significance was determined using the Mann–Whitney *U* test, Kruskal–Wallis test, unpaired Student's *t*‐test or one‐way analysis of variance (ANOVA). Differences between groups were judged statistically significant at *p* < .05.

## 3 RESULTS

### 3.1 Increased circGNB1 expression correlates with senescence, oxidative stress and OA severity

**FIGURE 1:**
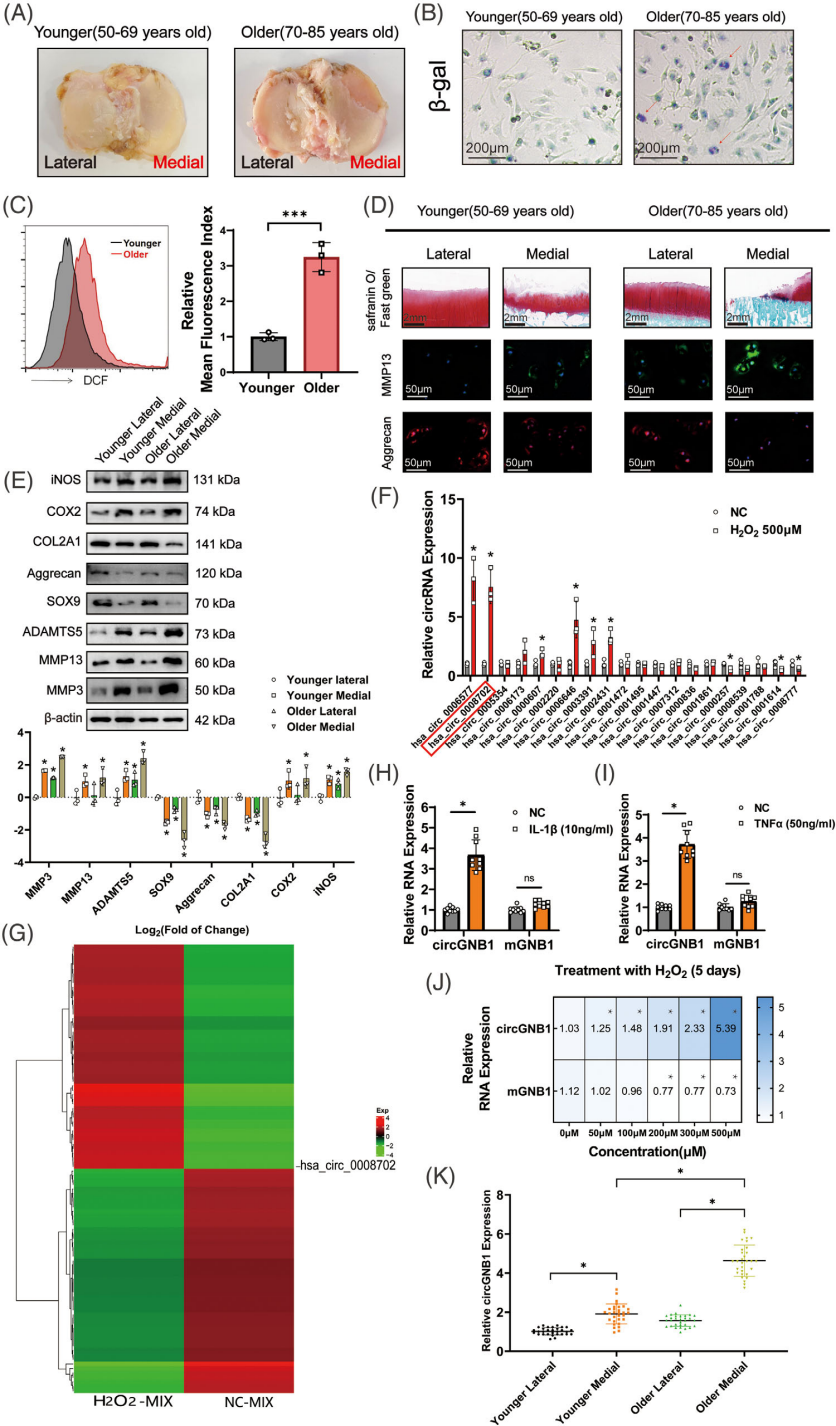
Higher expression of circGNB1 was found in the cartilage of older patients with more severe osteoarthritis (OA). (A) Representative photos of the tibial plateau from patients of various ages. (B) Representative images of primary chondrocytes senescence‐associated β‐galactosidase staining of medial articular cartilage from patients of different ages. Scale bars, 200 μm. (C) Flow cytometric analysis of primary chondrocytes oxygen species (ROS) activity in medial articular cartilage from patients of different ages. *n* = 3 (three different donors).***p < .001. (D) Representative images of Safranin O/fast green staining and Immunofluorescence labelling of MMP13 and Aggrecan in human knee cartilage. Scale bars, 2 mm and 50 μm. (E) Upper, western blot experiments of primary chondrocytes from particular specimens. Lower, using log2 (fold of change) to quantify western blot experiments. *n* = 3 (three different donors). *p < .05 compared to the primary chondrocytes in the Younger Lateral group. (F) Quantification of the top 20 differentially expressed circRNAs (10 up and 10 down) relative expression using quantitative real‐time PCR (qRT‐PCR) in human articular chondrocytes (HCs) stimulated by H_2_O_2_ (500 μM) for 48 h. *n* = 3 (three different donors). *p < .05 compared to negative control (NC). (G) Heat map of top 100 differentially expressed circRNAs (50 up and 50 down), with FDR ≤ .05 and | log2 (fold change) | > 1, between a mixture of five persons’ primary chondrocytes treated with 500 μM of H_2_O_2_ for 5 days (H_2_O_2_‐MIX) and a mixture of the same five persons’ negative control primary chondrocytes (NC‐MIX). (H) RNA levels of circGNB1 and mGNB1 in HCs stimulated by IL‐1β (10 ng/mL) for 48 h. *n* = 3 (three different donors for three different experiments). (I) RNA levels of circGNB1 and mGNB1 in HCs stimulated by TNF‐α (50 ng/mL) for 48 h. *n* = 3 (three different donors for three different experiments). (J) Blue‐scale heat maps showing the RNA levels of circGNB1 and mGNB1 in HCs stimulated by gradient concentrations of H_2_O_2_ (0‐500 μM) for 5 days. *n* = 3 (three different donors). *p < .05 compared to NC. (K) qRT‐PCR quantification of relative circGNB1 expression in HCs from different donors. *n* = 10 (10 different donors for three different experiments). *p*‐Values are shown in graphs and were determined using Mann‐Whitney U test (C and F), Kruskal–Wallis test (E and J), unpaired Student's *t*‐test (H and I) or one‐way ANOVA (K). Data were presented as means ± standard deviation.

We first characterized the presence of senescent cells and oxidative stress in the cartilage of patients of variable ages having OA. On macroscopic examination, the cartilage on the medial tibial plateau typically exhibits more pronounced deterioration and exposure of the subchondral bone than that on the lateral side. As expected, the cartilage on both the medial and lateral tibial plateaus from older patients showed more deterioration than that from younger patients (Figure 1A). Senescence‐associated‐gal staining showed a higher number of senescent cells in older patients than in younger patients (Figure 1B). Flow cytometric analysis announced a significant growth in ROS production in chondrocytes isolated from older patients, indicating greater oxidative stress in these cells (Figure 1C). The more severe loss of cartilage in older patients was confirmed by the loss of proteoglycan staining by Safranin O (Figure 1D), and this was corroborated by the increased severity of OARSI scores in older patients (Figure S1A). In agreement with the above results, immunofluorescence demonstrated increased expression of the catabolic enzyme metalloproteinase (MMP) 13, as well as a decrease in the cartilage matrix protein aggrecan (Figure 1D and Figure S1B).

Furthermore, western blotting presented that the expression of other catabolic enzymes, such as MMP3, MMP13, A disintegrin‐like, and MMP with thrombospondin motifs (ADAMTS) 5, was also markedly increased in older patients compared to younger patients (Figure 1E). In contrast, the expression levels of proteins required for matrix syntheses such as SRY‐box transcription factor 9 (SOX9), aggrecan, and collagen type II alpha 1 (COL2A1) were significantly lower in older patients (Figure 1E). Finally, the factors associated with oxidative stress such as inducible nitric oxide synthase (iNOS) and cyclooxygenase 2 (COX‐2), had higher expression levels in the older patients (Figure 1E). Collectively, these results indicate that the cartilage on the medial femoral condyle in older patients generally exhibits greater cellular senescence, oxidative stress, and OA severity than that in younger patients.

We investigated the relationship between oxidation‐induced circRNAs and OA severity. Previously, using RNA sequencing, we detected 296 up‐regulated and 254 down‐regulated circRNAs in chondrocytes of patients with OA.^28^ We then examined the 10 most up‐regulated and 10 most down‐regulated circRNAs in H_2_O_2_‐treated chondrocytes (Figure 1F). The quantitative real‐time PCR (qRT‐PCR) results showed that circRSU1 (hsa_circ_0006577) and circGNB1 (hsa_circ_0008702) had the top two highest expression among the circRNAs tested, since circRSU1 has been Investigated in our previous research, thus we choose circGNB1 as our target circRNA (Figure 1F,G).

As OA is a chronic inflammatory process involving oxidative stress,^29^ we examined the expression of circGNB1 in chondrocytes under inflammatory conditions. The results showed that circGNB1, but not its precursor linear mRNA (mGNB1), was considerably up‐regulated in chondrocytes treated with the pro‐inflammatory cytokine interleukin‐1β (IL‐1β) or tumor necrosis factor‐α (TNF‐α), compared to untreated controls (Figure 1H,I). Similarly, under increased oxidative stress, circGNB1 expression was significantly up‐regulated in a dose‐dependent manner in chondrocytes treated with H_2_O_2_, whereas it's linear precursor mRNA was slightly down‐regulated (Figure 1J). In addition, qRT‐PCR stood out that circGNB1 expression was higher in the medial condyle with more severe OA than in the lateral condyle and that its expression increased significantly with age (Figure 1K). These data indicated that circGNB1 takes a substantial part in age‐related OA and oxidative stress.

### 3.2 Confirmation of circGNB1 as circRNA

Next, we sought to confirm that circGNB1 is indeed a circRNA. According to the circRNA database (Circbase), circGNB1 is generated by the circularization of exons 2−5 of the GNB1 transcript (NM_002074, chr1, region 1747194−1770677) (Figure S1C). The back‐spliced junction between the 5ʹ end of exon 2 and the 3ʹ end of exon 5 was confirmed by sequencing the circGNB1 PCR product (Figure S1C). Previous studies reported that circRNAs are more resistant to RNase than their mRNA counterparts because of their closed‐loop structures.^23^ Indeed, RNase R treatment confirmed that circGNB1 was resistant to the treatment, whereas mGNB1 levels decreased significantly (Figure S1D). Moreover, treatment with the transcription inhibitor actinomycin D decreased mGNB1 expression, as expected, but did not affect circGNB1 expression (Figure S1E). To verify that native circGNB1 was specifically backspliced, convergent and divergent primers were designed and used. circGNB1 was detected only in cDNA, whereas mGNB1 was detected in both cDNA and genomic DNA (gDNA) (Figure S1F). In addition, FISH and nuclear‐cytoplasmic separation experiments illustrated that circGNB1 was mainly present in the cytoplasm rather than in the nucleus (Figure S1G,H). Collectively, these results confirm that circGNB1 is a circRNA.

### 3.3 The role of circGNB1 in regulating OA phenotypes in chondrocytes

**FIGURE 2:**
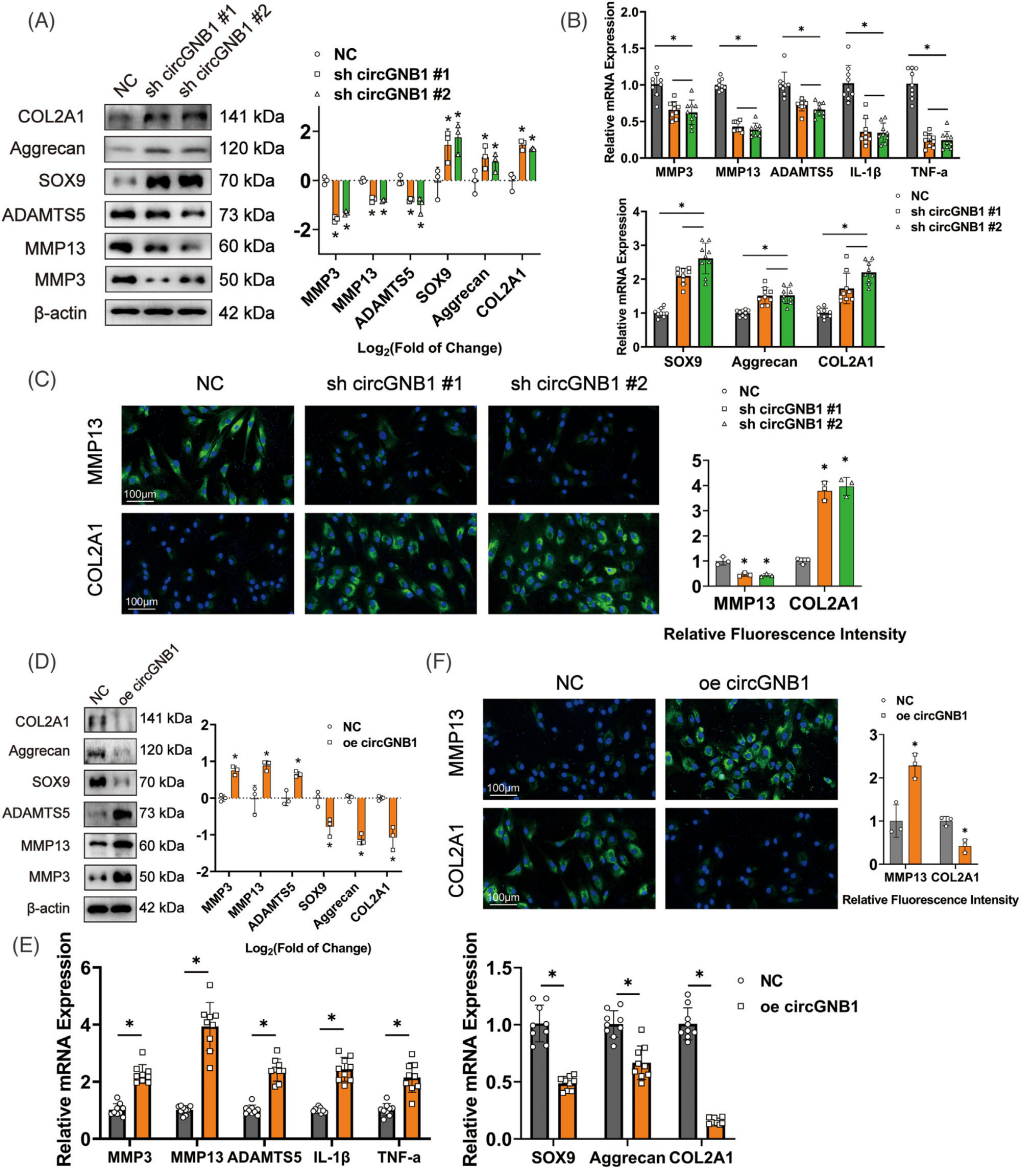
Changes in the expression of circGNB1 correspond to the severity of osteoarthritis (OA) phenotypes in chondrocytes. (A) Left, western blot experiment of extracellular matrix (ECM) associated proteins after down‐regulation of circGNB1. Right, using log2 (fold of change) to quantify western blot experiments. *n* = 3 (three different donors). *p < .05. (B) Quantification of relative RNA levels associated with catabolic enzymes, synthetase and proteoglycans using quantitative real‐time PCR (qRT‐PCR) after down‐regulation of circGNB1. *n* = 3 (three different donors for three different experiments). *p < .05. (C) Left, representative images of immunofluorescence labeling of MMP13 and COL2A1 after down‐regulation of circGNB1. Scale bars, 100 μm. Right, quantification of immunofluorescence with relative fluorescence intensity. *n* = 3 (three different donors). *p < .05. (D) Left, western blot experiment of extracellular matrix (ECM) associated proteins after overexpression of circGNB1. Right, using log2 (fold of change) to quantify western blot experiments. *n* = 3 (three different donors). *p < .05. (E) Quantification of relative RNA levels associated with catabolic enzymes, pro‐inflammatory cytokines, synthetase and proteoglycans using qRT‐PCR in human chondrocytes (HCs) overexpressing circGNB1. *n* = 3 (three different donors for three different experiments). *p < .05. (F) Left, representative images of Immunofluorescence labelling of MMP13 and COL2A1 after overexpression of circGNB1. Scale bars, 100 μm. Right, quantification of immunofluorescence with relative fluorescence intensity. *n* = 3 (three different donors). *p < .05. *p*‐Values are shown in graphs and were determined using Mann–Whitney *U* test (D and F), Kruskal–Wallis test (A and C), unpaired Student's *t*‐test (E) or one‐way ANOVA (B). Data were presented as means ± standard deviation.

Gain‐ and loss‐of‐function analyses were performed to discover the biological functions of circGNB1 in the human articular chondrocytes (HCs). Two lentiviral shRNA plasmids targeting different sites on circGNB1 (sh circGNB1 #1 and sh circGNB1 #2) were generated. qRT‐PCR showed that both sh circGNB1#1 and sh circGNB1#2 significantly reduced circGNB1 expression but not the expression of the linear precursor mGNB1 (Figure S1I). Western blotting and qRT‐PCR showed that in chondrocytes with stable circGNB1 down‐regulation, the expression of the catabolic enzymes MMP3, MMP13, and ADAMTS5 was significantly down‐regulated. In contrast, the expression of the anabolic factors SOX9, Aggrecan, and COL2A1 was significantly elevated (Figure 2A,B). In addition, mRNA expression of pro‐inflammatory factors IL‐1β and TNF‐α was substantially reduced in circGNB1 knockdown cells (Figure 2B). Immunofluorescence confirmed that a decrease in circGNB1 expression cause a decrease in MMP13 and a corresponding increase in COL2A1 protein levels in HCs (Figure 2C). These data indicated that a decrease in circGNB1 expression was associated with a less severe OA phenotype.

Next, we investigated the effect of overexpression circGNB1 on HCs. HCs stably overexpressing circGNB1 were generated by transduction with a lentiviral construct harboring circGNB1 (oe‐circGNB1). These cells expressed significantly higher circGNB1 levels, but not the linear precursor mGNB1 (Figure S1J). Western blotting and qRT‐PCR illustrated that circGNB1 up‐regulation appreciably promoted the expression of MMP3, MMP13, and ADAMTS5 while down‐regulating the expression of SOX9, Aggrecan, and COL2A1 (Figure 2D,E). The mRNA expression of pro‐inflammatory factors IL‐1β and TNF‐α was considerably augmented in cells overexpressing circGNB1 (Figure 2E). Immunofluorescence also confirmed that circGNB1 overexpression promoted MMP13 up‐regulation but decreased COL2A1 expression in HCs (Figure 2F).

By and large, these data imply that circGNB1 expression positively correlates with OA phenotypes in chondrocytes, indicating that circGNB1 plays a role in OA development.

### 3.4 CircGNB1 functions as a miR‐152‐3p sponge to potentiate OA

Previous researches have shown that circRNAs bind to miRNAs and competitively inhibit their interactions with endogenous targets in the cytoplasm.^16^ Given that circGNB1 is chiefly localized in the cytoplasm of HCs, we tested whether it was associated with miRNAs and the RNA‐induced silencing complex (RISC). We found that circGNB1 was significantly enriched in RIP using an antibody against AGO2, which binds miRNAs and RISC in the cytoplasm (Figure S2A), suggesting that circGNB1 functions as an miRNA sponge. By cross‐referencing the miRanda, RNAbybrid, and TargetScan databases, we identified 19 candidate miRNAs as potential targets of circGNB1 (Figure S2B). Next, we designed and validated an antisense RNA probe specific for circGNB1 (Figure S2C). Using RAP, we identified six miRNAs (miR‐6843‐3p, miR‐933, miR‐152‐3p, miR‐6829‐3p, miR‐3074‐5p, and miR‐22‐5p) associated with circGNB1 (Figure S2D). Among these six miRNAs, miR‐152‐3p exhibits the highest degree of conservation among mammals based on the TargetScan database. To validate the interaction between circGNB1 and six miRNAs, we co‐transfected HEK‐293T cells with plasmids expressing various miRNA mimics and a luciferase reporter fused to the circGNB1 sequence. Only cells transfected with miR‐152‐3p, miR‐6829‐3p, or miR‐3074‐5p mimics showed a significant reduction in relative luciferase activity (Figure S2E). Importantly, in H_2_O_2_‐stimulated chondrocytes, the expression of miR‐152‐3p was strikingly suppressed, whereas that of miR‐6829‐3p and miR‐3074‐5p was elevated (Figure S2F). Therefore, we hypothesized that miR‐152‐3p is the main downstream target of circGNB1.

Next, we constructed luciferase reporters expressing either wild‐type (Luc‐circGNB1 WT) or mutant (Luc‐circGNB1 Mut) circGNB1, which did not target miR‐152‐3p (Figure S2G). We discovered that the miR‐152‐3p mimic significantly reduced luciferase signals from Luc‐circGNB1 WT but not in cells transfected with Luc‐circGNB1 Mut or the negative control (Figure S2H). These results demonstrate a sequence‐specific interaction between circGNB1 and miR‐152‐3p. This was further corroborated by the colocalization of circGNB1 and miR‐152‐3p in the cytoplasm of HCs, as shown using FISH (Figure S3A). In addition, our data showed that in contrast to wild‐type circGNB1, mutant circGNB1 defective in miR‐152‐3p binding did not enhance the protein expression of OA markers (Figure S3B). Then, we construct miR‐152‐3p sponge adenovirus, which contains miR‐152‐3p repeat complementary binding sites that inhibit its function. By co‐transfecting sh circGNB1 and miR‐152‐3p sponge adenovirus to HC cells, we found that sh circGNB1+miR‐152‐3p sponge rescued the mRNA changes in OA marker compare to sh circGNB1 (Figure S3C). There was also an inverse relationship between the expression of circGNB1 and its target miR‐152‐3p, as circGNB1 expression was the highest in patients with more advanced OA (Figure 1K), whereas miR‐152‐3p expression was the highest in younger patients with less severe OA (Figure S3D).

**FIGURE 3:**
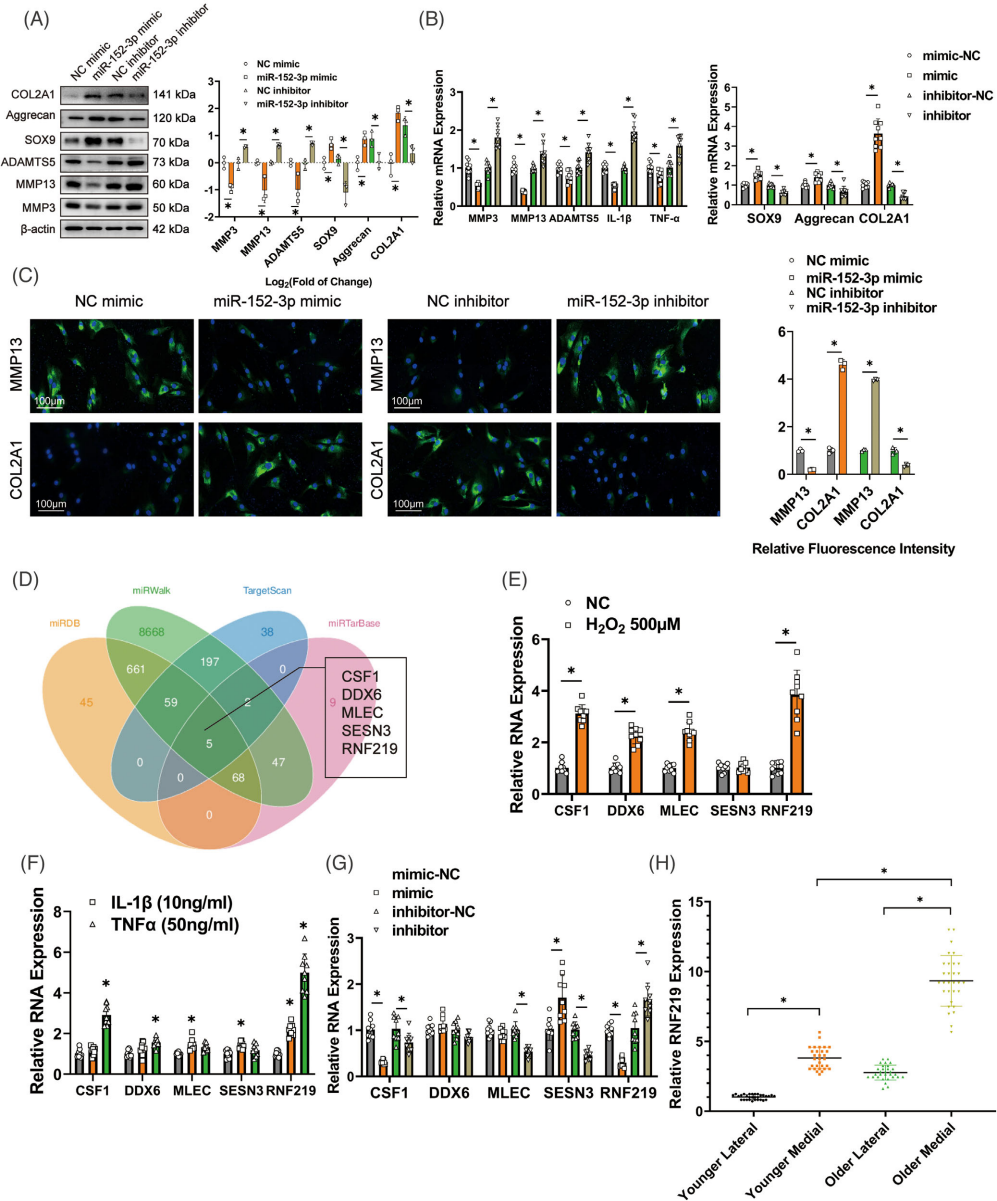
MiR‐152‐3p inhibits the progression of osteoarthritis (OA) in human chondrocytes (HCs) via mediating the downstream target gene RNF219. (A) Left, western blot showed expression of catabolic and anabolic genes in HCs overexpressing or deficient in miR‐152‐3p. Right, quantification of protein bands presented as log2 fold‐change over controls. *n* = 3 (three different donors). *p < .05. (B) qRT‐PCR of expression of catabolic (MMP3, MMP13, and ADAMTS5) and anabolic factors (SOX9, Aggrecan, COL2A1), as well as pro‐inflammatory cytokines (IL‐1β and TNF‐α) in cells overexpressing or deficient in miR‐152‐3p. *n* = 3 (three different donors for three different experiments). *p < .05. (C) Left, representative images of immunofluorescence labelling of MMP13 and COL2A1 in cells overexpressing or deficient in miR‐152‐3p. Scale bars = 100 μm. Right, quantification of fluorescence intensity relative to respective controls. *n* = 3 (three different donors). *p < .05. (D) Potential candidate downstream mRNA targets of miR‐152‐3p by cross referencing miRDB, miRWalk, TargetScan, and miRTarBase databases. (E) qRT‐PCR of candidate mRNA expression in HCs stimulated by H_2_O_2_ (500 μM) for 48 h. *n* = 3 (three different donors for three different experiments). *p < .05. (F) qRT‐PCR of the candidate miR‐152‐3p target mRNAs in HCs stimulated by IL‐1β (10 ng/mL) or TNF‐α (50 ng/mL) for 48 h. *n* = 3 (three different donors for three different experiments). *p < .05. (G) qRT‐PCR of candidate miR‐152‐3p target mRNAs in cells overexpressing or deficient in miR‐152‐3p. *n* = 3 (three different donors for three different experiments). *p < .05. (H) qRT‐PCR of relative RNF219 expression in HCs from different donors. *n* = 10 (10 different donors for three different experiments). *p < .05. *p*‐Values are shown in graphs and were determined using Mann–Whitney *U* test (A and C), unpaired Student's *t*‐test (B, E, and G) or one‐way ANOVA (F and H). Data were presented as means ± standard deviation.

Next, we looked into the character of miR‐152‐3p in OA progression in HCs using gain‐ and loss‐of‐function analyses. Figure S3E shows the high transfection efficiency of the miR‐152‐3p mimic and miR‐152‐3p inhibitor in HCs. Western blotting pointed out that miR‐152‐3p overexpression declined the expression of MMP3, MMP13, and ADAMTS5 while increasing the expression of SOX9, Aggrecan, and COL2A1. On the contrary, miR‐152‐3p down‐regulation had the inverse effect (Figure 3A). Similar results were obtained by qRT‐PCR. In addition, we observed that miR‐152‐3p positively regulates the mRNA levels of IL‐1β and TNF‐α (Figure 3B). Immunofluorescence staining verified that miR‐152‐3p appreciably restrained MMP13 expression and consequently enhanced the COL2A1 expression in HCs. (Figure 3C).

Next, we identified potential downstream target genes of circGNB1/miR‐152‐3p, regarded as the circRNA—miRNA—mRNA regulatory axis. Five candidate downstream target genes (CSF1, DDX6, MLEC, SESN3, and RNF219) were identified by cross‐referencing miRDB, miRWalk, TargetScan, and miRTarBase databases (Figure 3D). Among these five candidates, RNF219 expression was the highest In HCs stimulated with H_2_O_2,_ IL‐1β, or TNF‐α (Figure 3E,F).

To test whether these five candidate genes were direct targets of miR‐152‐3p, miR‐152‐3p was overexpressed or knocked down in HCs. The results illustrated that miR‐152‐3p overexpression significantly suppressed the mRNA expression of CSF1 and RNF219, whereas miR‐152‐3p down‐regulation significantly up‐regulated RNF219 mRNA (Figure 3G). Therefore, RNF219 has a tendency to be the main downstream target of the circGNB1/miR‐152‐3p axis. To confirm this, luciferase constructs were generated based on wild‐type RNF219 or mutant RNF219 defective in miR‐152‐3p interaction (Luc‐RNF219 WT and Luc‐RNF219 Mut) (Figure S3F). Luciferase assays demonstrated that miR‐152‐3p suppressed the wild‐type but not the mutant RNF219 luciferase construct (Figure S3G). In HCs, we found that RNF219 expression was the highest in the medial cartilage of older patients with the most severe OA (Figure 3H), which is consistent with the circGNB1 expression trend (Figure 1K).

### 3.5 RNF219 is a downstream target of the circGNB1/miR‐152‐3p axis in HCs

**FIGURE 4:**
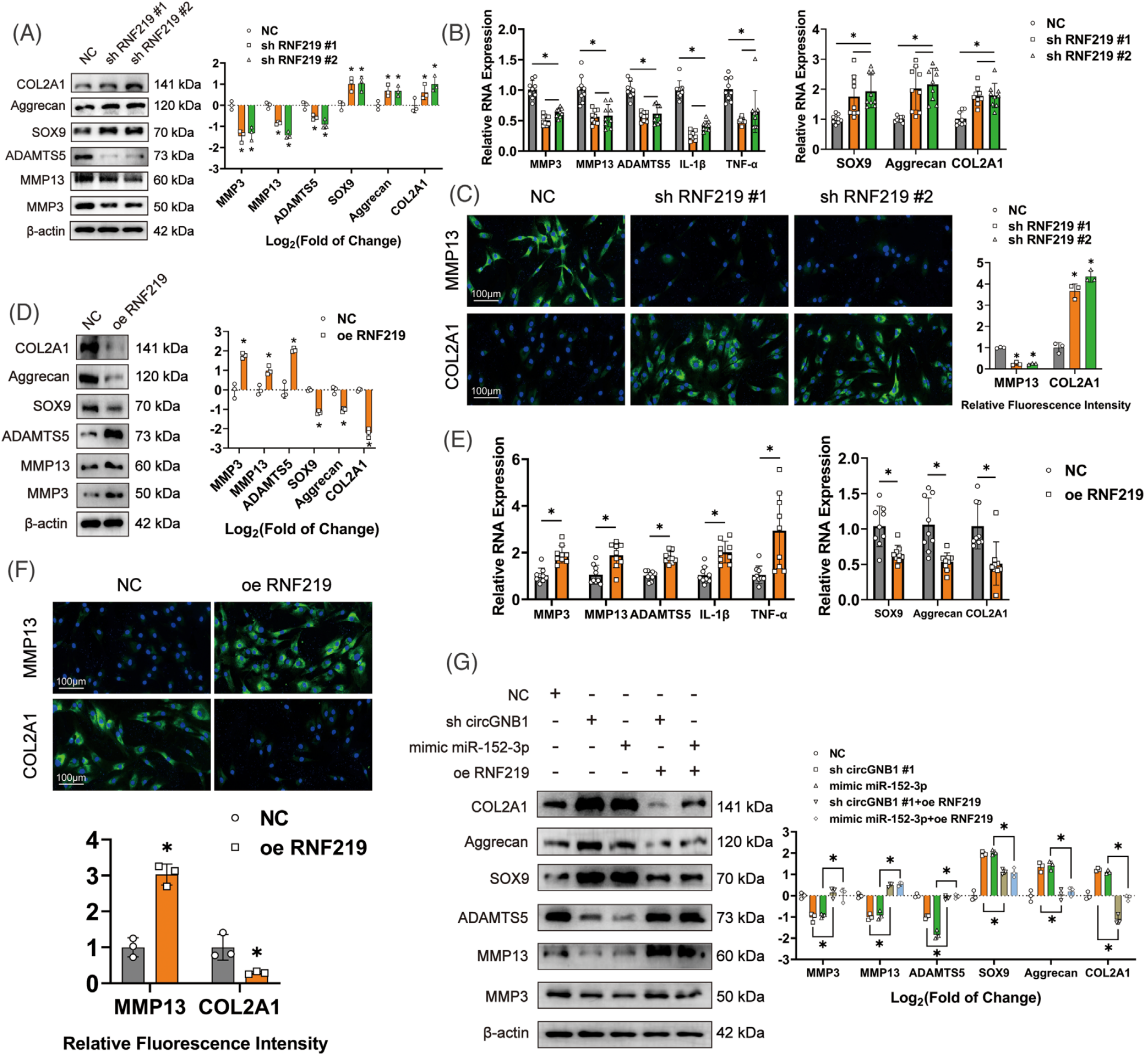
RNF219 promotes the progression of osteoarthritis (OA) in human chondrocytes (HCs). (A) Left, western blot showed expression of catabolic and anabolic genes in HCs deficient in RNF219 expression. Right, quantification of protein bands presented as log2 fold‐change over controls. *n* = 3 (three different donors). *p < .05. (B) qRT‐PCR of relative mRNA levels of catabolic and anabolic factors, as well as pro‐inflammatory cytokines in HCs deficient in RNF219. *n* = 3 (three different donors for three different experiments). *p < .05. (C) Left, representative immunofluorescence staining of MMP13 and COL2A1 after down‐regulation of RNF219. Scale bars = 100 μm. Right, quantification of immunofluorescence intensity relative to controls. *n* = 3 (three different donors). *p < .05. (D) Left, western blots of OA marker expression in HCs overexpressing RNF219. Right, quantification of protein bands presented as log2 fold‐change over controls. *n* = 3 (three different donors). *p < .05. (E) qRT‐PCR of relative mRNA levels of catabolic and anabolic factors, as well as pro‐inflammatory cytokines in HCs overexpressing in RNF219. *n* = 3 (three different donors for three different experiments). *p < .05. (F) Upper panel, representative Immunofluorescence staining of MMP13 and COL2A1 in HCs overexpressing RNF219. Scale bars = 100 μm. Lower panel, quantification of immunofluorescence intensity relative to controls. *n* = 3 (three different donors). *p < .05. (G) Left, western blot experiment of OA marker proteins in HCs expressing various levels of circGNB1, miR‐152‐3p, or RNF219. Right, quantification of protein bands presented as log2 fold‐change over controls. *n* = 3 (three different donors). *p < .05. *p*‐Values are shown in graphs and were determined using Mann‐Whitney U test (D and F), Kruskal–Wallis test (A, C, and G), unpaired Student's *t*‐test (E) or one‐way ANOVA (B). Data were presented as means ± standard deviation.

We explored the role of RNF219 in OA progression in HCs using gain‐ and loss‐of‐function analyses. The mRNA expression of RNF219 was suppressed in HCs expressing RNF219 targeting shRNAs (sh RNF219#1 and sh RNF219#2) (Figure S3H). Western blotting showed that RNF219 down‐regulation in HCs inhibited the expression of MMP3, MMP13, and ADAMTS5 while promoting the expression of SOX9, Aggrecan, and COL2A1 (Figure 4A). Similar results were obtained from qRT‐PCR, and the mRNA levels of IL‐1β and TNF‐α were also down‐regulated by suppressing RNF219 expression (Figure 4B). Immunofluorescence staining also showed that RNF219 inhibition significantly decreased MMP13 expression but increased COL2A1 expression (Figure 4C). Next, we determined the effect of RNF219 overexpression in HCs stably transduced with a lentivirus harboring RNF219 (oe‐RNF219) (Figure S3I). Western blotting showed that RNF219 overexpression notably raised the expression of MMP3, MMP13 and ADAMTS5, but restrained the expression of SOX9, Aggrecan, and COL2A1 (Figure 4D). Similar outcomes were obtained by qRT‐PCR, and RNF219 overexpression also increased the mRNA expression of IL‐1β and TNF‐α (Figure 4E). Immunofluorescence also showed that RNF219 overexpression significantly increased MMP13 protein expression but decreased COL2A1 expression (Figure 4F).

Next, we tested whether RNF219 overexpression could counter the effects of circGNB1 overexpression or miR‐152‐3p knockdown on the promotion of OA phenotypes. Western blotting showed that RNF219 overexpression prevented the corresponding changes in OA phenotypes caused by circGNB1 knockdown or miR‐152‐3p overexpression in HCs in terms of MMP3, MMP13, ADAMTS5, SOX9, Aggrecan and COL2A1 expression (Figure 4G). In general, these data demonstrated that circGNB1 regulates OA progression by adjusting RNF219 expression through competitive binding to miR‐152‐3p.

### 3.6 CAV1 is a novel RNF219 binding partner promoting human articular chondrocyte OA

**FIGURE 5:**
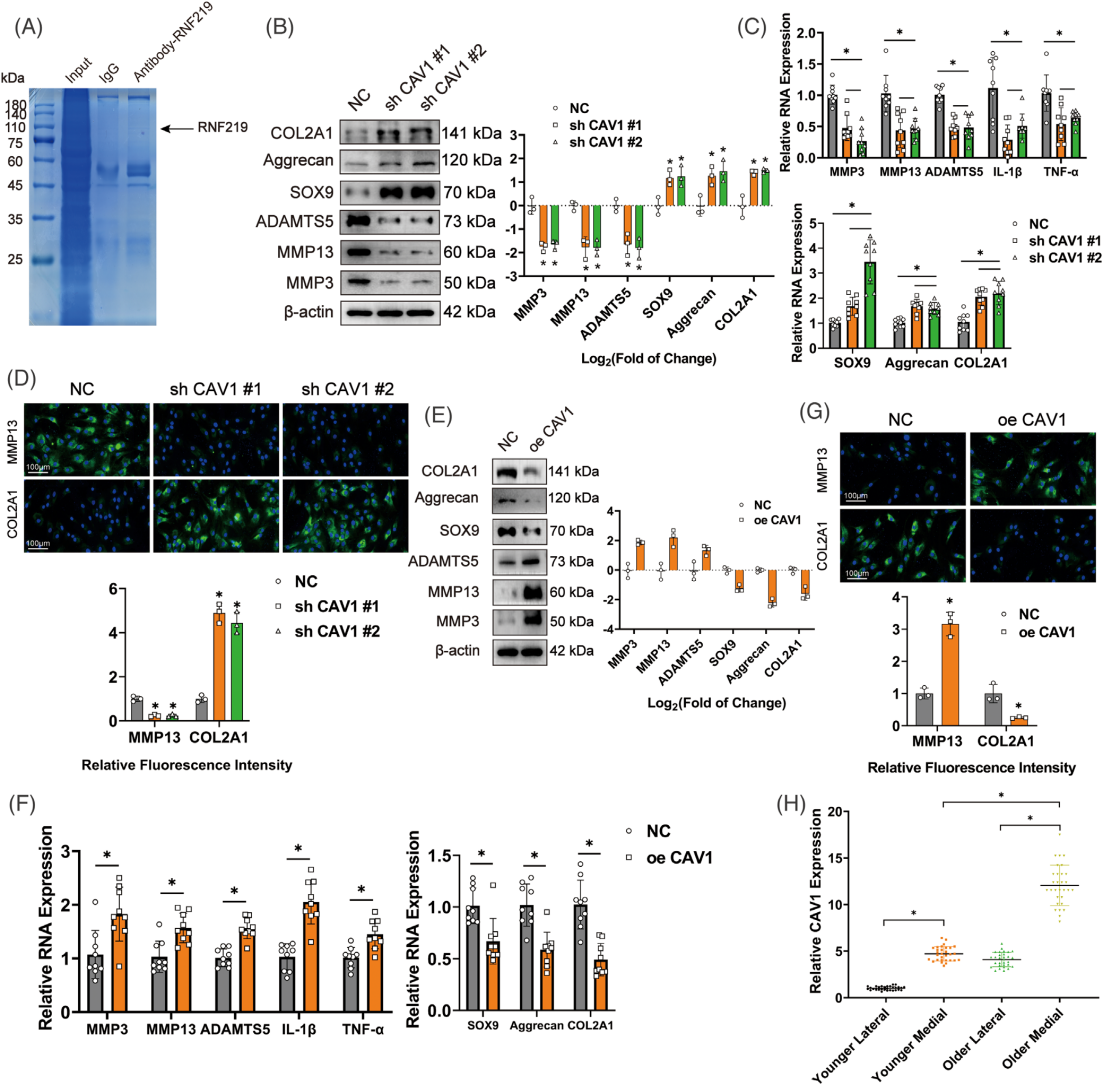
CAV1 is a novel RNF219‐binding protein and promotes osteoarthritis (OA) in human chondrocytes (HCs). (A) Coomassie blue staining of immunoprecipitated RNF219 and associated proteins. (B) Left, western blot showed expression of catabolic and anabolic genes in HCs deficient in CAV1. Right, quantification of protein bands presented as log2 fold‐change over controls. *n* = 3 (three different donors). *p < .05. (C) qRT‐PCR of relative mRNA levels of catabolic and anabolic factors, as well as pro‐inflammatory cytokines in HCs deficient in CAV1. *n* = 3 (three different donors for three different experiments). *p < .05. (D) Upper, representative Immunofluorescence images of MMP13 and COL2A1 after down‐regulation of CAV1 in HCs. Scale bars = 100 μm. Lower, quantification of immunofluorescence intensity relative to controls. *n* = 3 (three different donors). *p < .05. (E) Left, western blots of catabolic and anabolic factors in HCs overexpressing CAV1. Right, quantification of protein bands presented as log2 fold‐change over controls. *n* = 3 (three different donors). *p < .05. (F) qRT‐PCR of relative mRNA levels of catabolic and anabolic factors, as well as pro‐inflammatory cytokines in HCs overexpressing CAV1. *n* = 3 (three different donors for three different experiments). *p < .05. (G) Upper, representative Immunofluorescence images of MMP13 and COL2A1 in HCs after overexpressing CAV1. Scale bars = 100 μm. Lower, quantification of immunofluorescence intensity relative to controls. *n* = 3 (three different donors). *p < .05. (H) qRT‐PCR of relative CAV1 expression in HCs from different donors. *n* = 10 (10 different donors for three different experiments). *p < .05. *p*‐Values are shown in graphs and were determined using Mann–Whitney *U* test (E and G), Kruskal–Wallis test (B and D), unpaired Student's *t*‐test (F) or one‐way ANOVA (C and H). Data were presented as means ± standard deviation.

To further investigate the mechanisms by which RNF219 exerts its biological functions in OA progression, RNF219‐associated proteins were immunoprecipitated (Figure 5A), and the top 10 candidate RNF219‐binding proteins were identified using liquid chromatography‐mass spectrometry (Figure S4A). Among these, only the mRNA expression of MYO18A, CAV1 and UGDH was elevated, while that of CLTC was down‐regulated in HCs treated with IL‐1β or TNF‐α‐treated (Figure S4A). Alternatively, the mRNA expression levels of PLEC and CAV1 were elevated in H_2_O_2_‐treated HCs (Figure S4B). Therefore, these five genes (MYO18A, PLEC, CAV1, CLTC, and UGDH) could play important roles in chondrocytes under inflammatory stress. We designed siRNAs against these five genes and validated the efficient knockdown of gene targets using qRT‐PCR (Figure S4C). Validation by western blotting and qRT‐PCR, we found that CAV1 had a significant role in OA marker expression, as knockdown of CAV1 suppressed the expression of MMP3, MMP13, and ADAMTS5 and promoted the expression of SOX9, Aggrecan and COL2A1. The expression of pro‐inflammatory factors related to oxidative stress, for instance, yclooxygenase‐2 (COX2) and inducible nitric oxide synthase (iNOS), also significantly declined (Figure S4D,E). Thus, we conclude that CAV1 is a novel RNF219‐binding partner and may take a significant part in OA progression in HCs.

To further investigate the role of CAV1, lentiviruses harboring siRNAs against CAV1 (sh CAV1#1 and sh CAV1#2) were stably transduced into HCs, and the knockdown efficiency of CAV1 mRNAs was confirmed (Figure S4F). Western blotting showed that CAV1 down‐regulation significantly suppressed the expression of MMP3, MMP13 and ADAMTS5 but promoted the expression of SOX9, Aggrecan and COL2A1 proteins (Figure 5B). Similar findings were obtained by qRT‐PCR, and knockdown of CAV1 also reduced the mRNA expression of IL‐1β and TNF‐α (Figure 5C). Immunofluorescence labelling also confirmed that the inhibition of CAV1 expression reduced OA phenotypes by reducing MMP13 and increasing COL2A1 protein expression (Figure 5D).

Next, we investigated the effects of CAV1 overexpression in HCs stably transduced with lentiviruses harboring CAV1 (oe‐CAV1) (Figure S4G). Western blotting showed that CAV1 overexpression advanced the expression of MMP3, MMP13 and ADAMTS5, but inhibited the expression of SOX9, Aggrecan and COL2A1 (Figure 5E). qRT‐PCR yielded similar findings, and CAV1 overexpression upregulated the mRNA expression of IL‐1β and TNF‐α (Figure 5F). Immunofluorescence staining demonstrated that CAV1 overexpression promoted MMP13 expression, but reduced COL2A1 expression (Figure 5G).

At the tissue level, CAV1 mRNA expression was the highest in the medial cartilage of older patients (Figure 5H), which also coincided with the expression trends of circGNB1 (Figure 1K) and RNF219 (Figure 3H). In summary, these data showed that CAV1 is a new binding partner of RNF219 and may take an essential part in OA progression in HCs.

### 3.7 The circGNB1/miR‐152‐3p/RNF219 axis prevents CAV1 degradation by inhibiting its ubiquitination

**FIGURE 6:**
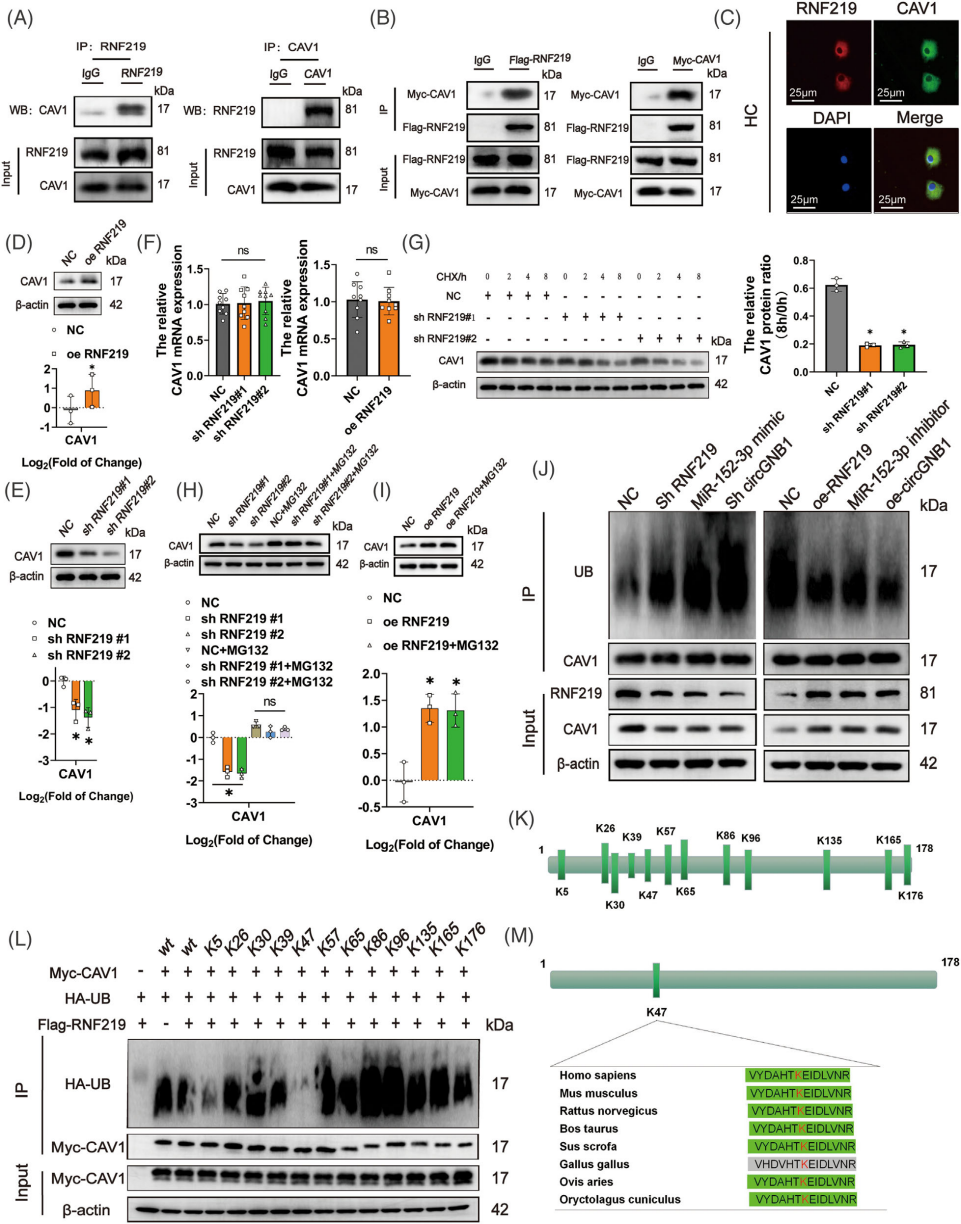
The circGNB1/miR‐152‐3p/RNF219 axis inhibits ubiquitination of CAV1 at residue K47. (A) Western blots of co‐immunoprecipitated endogenous RNF219 and CAV1 in human chondrocytes (HCs). (B) Western blots of co‐immunoprecipitated Flag‐RNF219 and Myc‐CAV1 in transfected HEK‐293T cells. (C) Immunofluorescence showing co‐localization of RNF219 and CAV1 in HCs. Scale bars = 25 μm. (D) Upper, western blots of CAV1 in HCs overexpressing RNF219. Lower, quantification of fold‐change (log2) in protein bands density. *n* = 3 (three different donors). *p < .05. (E) Upper, western blots of CAV1 in HCs deficient in RNF219. Lower, quantification of fold‐change (log2) in protein bands density. *n* = 3 (three different donors). *p < .05. (F) The relative mRNA expression of CAV1 after RNF219 knockdown and overexpression. *n* = 3 (three different donors for three different experiments). (G) Western blot quantification of CAV1 in HCs deficient in RNF219, in the presence of 200 μg/mL cycloheximide (CHX) at different times. *n* = 3 (three different donors). *p < .05. (H) Upper, western blot of CAV1 in the presence or absence of proteosome inhibitor MG132 in RNF219‐knockdown HCs. Lower, quantification of fold‐change (log2) in protein bands density. *n* = 3 (three different donors). *p < .05. (I) Upper, effect of MG132 treatment on CAV1 protein level mediated by RNF219 overexpression. Lower, quantification of fold‐change (log2) in protein bands density. *n* = 3 (three different donors). *p < .05. (J) Western blot of anti‐CAV1 IP showing ubiquitinated CAV1 in HCs expressing different levels of RNF219, miR‐152‐3p, or circGNB1. (K) Potential ubiquitylation sites of CAV1 from Pubmed database. (L) Western blot of IP samples from MG132‐treated cells co‐transfected with Myc‐tagged wildtype CAV1 or mutants and HA‐tagged ubiquintin. (M) Sequence alignment showing the K47 residue is conserved in mammalian CAV1. *p*‐Values are shown in graphs and were determined using Mann–Whitney *U* test(D), Kruskal–Wallis test (E, G, H, and I), unpaired Student's *t*‐test (F Right) or one‐way ANOVA (F Left). Data were presented as means ± standard deviation.

The interaction between RNF219 and CAV1 was verified using immunoprecipitation assays, as both endogenous and tagged RNF219 and CAV1 proteins were co‐immunoprecipitated using anti‐RNF219 or anti‐CAV1 antibodies in HCs (Figure 6A) and anti‐FLAG or anti‐Myc antibodies in transfected HEK‐293T cells (Figure 6B). Immunofluorescence staining also showed that RNF219 and CAV1 were colocalized in the cytoplasm of HCs (Figure 6C). We next investigated the effects of RNF219 expression on CAV1 ubiquitination because proteins with RING finger motifs (RNFs) are linked to the ubiquitination pathway.^30^ Our results showed that RNF219 overexpression significantly increased CAV1 protein expression (Figure 6D) but did not increase the expression of CAV1 mRNA (Figure 6F). Moreover, CAV1 degradation was accelerated in the presence of cycloheximide (CHX) when RNF219 was knocked down (Figure 6E,G). Importantly, RNF219‐mediated changes in CAV1 degradation were not detected in the presence of the proteasome inhibitor, MG132 (Figure 6H,I). We then constructed an RNF219 plasmid with a mutation in the RING domain (Figure S4H), which showed that mutant RNF219 could also stabilize CAV1 (Figure S4I). These results indicated that RNF219 antagonizes the ubiquitin‐dependent degradation of CAV1 in an independent of E3 ligands manner. To further confirm this, IP experiments were used to show that CAV1 ubiquitination increased when RNF219 or circGNB1 was knocked down, or miR‐152‐3p was overexpressed. However, CAV1 ubiquitination decreased when RNF219 or circGNB1 was overexpressed or miR‐152‐3p was knocked down (Figure 6J).

Next, we sought to identify the ubiquitination sites (s) in CAV1. Twelve lysine residues were identified as potential ubiquitination sites in CAV1 using the PubMed Gene database (Figure 6K). These 12 lysine residues were individually mutated to arginine, and K47R showed a significant reduction in CAV1 ubiquitination (Figure 6L), indicating that K47 is the major ubiquitination site. Interestingly, sequence alignment showed that K47 is highly conserved among mammalian CAV1 proteins, suggesting an important function of this residue (Figure 6M). In summary, these results pointed out that the circGNB1/miR‐152‐3p/RNF219 axis regulates CAV1 ubiquitination via the K47 residue.

### 3.8 The circGNB1/miR‐152‐3p/RNF219/CAV1 axis is associated with oxidative stress in HCs

**FIGURE 7:**
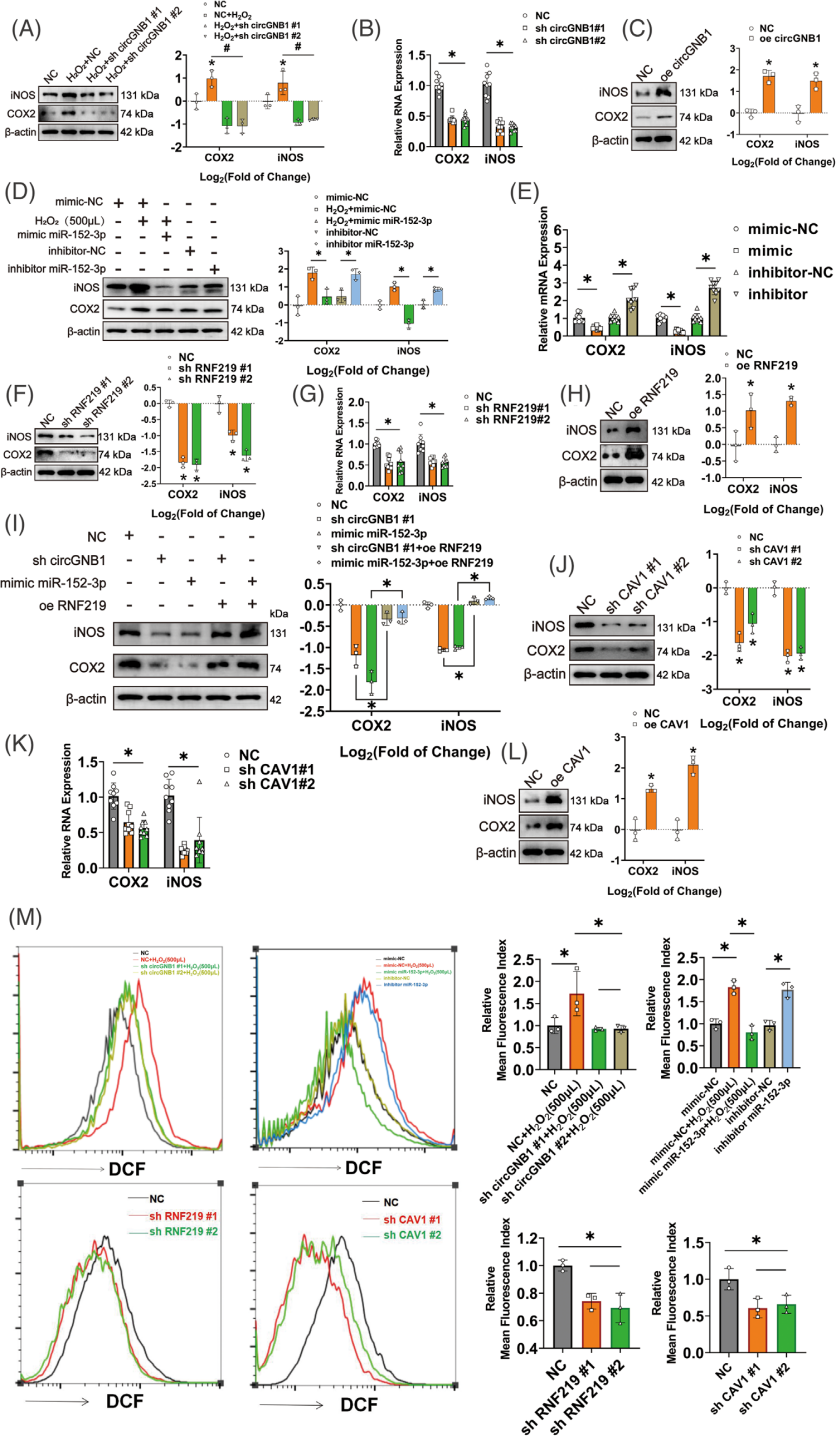
The circGNB1/miR‐152‐3p/RNF219/CAV1 axis is associated with oxidative stress in human chondrocytes (HCs). (A) Left, western blot of reactive oxygen species (ROS)‐associated pro‐inflammatory proteins (iNOS and COX2) in circGNB1‐knockdown HCs with or without H_2_O_2_ (500 μM) stimulation. Right, quantification of fold‐change (log2) in protein bands density. *n* = 3 (three different donors). *p < .05. (B) qRT‐PCR of mRNA levels of ROS‐associated proteins in circGNB1‐knockdown HCs. *n* = 3 (three different donors for three different experiments). *p < .05. (C) Left, western blots of ROS‐associated proteins in HCs overexpressing circGNB1. Right, quantification of fold‐change (log2) in protein bands density. *n* = 3 (three different donors). *p < .05. (D) Left, western blots of ROS‐associated pro‐inflammatory proteins in HCs overexpressing or deficient in miR‐152‐3p, with or without H_2_O_2_ (500 μM) stimulation. Right, quantification of fold‐change (log2) in protein bands density. *n* = 3 (three different donors). *p < .05. (E) Quantification of relative mRNA levels of ROS‐associated pro‐inflammatory cytokines using qRT‐PCR in HCs overexpressing or deficient in miR‐152‐3p. *n* = 3 (three different donors for three different experiments). *p < .05. (F) Left, western blots of ROS‐associated pro‐inflammatory proteins in HCs deficient in RNF219. Right, quantification of fold‐change (log2) in protein bands density. *n* = 3 (three different donors). *p < .05. (G) Quantification of relative RNA levels of ROS‐associated pro‐inflammatory cytokines using qRT‐PCR in HCs deficient in RNF219. *n* = 3 (three different donors for three different experiments). *p < .05. (H) Left, western blots of ROS‐associated pro‐inflammatory proteins in HCs overexpressing RNF219. Right, quantification of fold‐change (log2) in protein bands density. *n* = 3 (three different donors). *p < .05. (I) Left, western blots of ROS‐associated pro‐inflammatory proteins after in HCs overexpressing or deficient in circGNB1,miR‐152‐3p, or RNF219. Right, quantification of fold‐change (log2) in protein bands density. *n* = 3 (three different donors). *p < .05. (J) Left, western blots of ROS‐associated pro‐inflammatory proteins in HCs deficient in CAV1. Right, quantification of fold‐change (log2) in protein bands density. *n* = 3 (three different donors). *p < .05. (K) Quantification of relative mRNA levels of ROS‐associated pro‐inflammatory cytokines using qRT‐PCR in CAV1‐knockdown HCs. *n* = 3 (three different donors for three different experiments). *p < .05. (L) Left, western blots of ROS‐associated pro‐inflammatory proteins in HCs overexpressing CAV1. Right, quantification of fold‐change (log2) in protein bands density. *n* = 3 (three different donors). *p < .05. (M) Flow cytometric analysis of ROS activity in HCs overexpressing or deficient in circGNB1, miR‐152‐3p, RNF219, or CAV1, with or without H_2_O_2_ (500 μM) stimulation. *n* = 3 (three different donors). *p < .05. *p*‐Values are shown in graphs and were determined using Mann–Whitney *U* test (C, H, and L), Kruskal–Wallis test (A, D, F, I, J, and M), unpaired Student's *t*‐test (E) or one‐way ANOVA (B, G, and K). Data were presented as means ± standard deviation.

As the levels of the circGNB1/miR‐152‐3p/RNF219/CAV1 axis components were significantly altered in H_2_O_2_‐treated chondrocytes, we performed a detailed investigation of their roles in chondrocytes under oxidative stress. First, circGNB1 down‐regulation inhibited H_2_O_2_‐induced COX2 and iNOS protein and mRNA expression (Figure 7A,B) and decreased ROS production in HCs (Figure 7M). In contrast, circGNB1 promoted COX2 and iNOS protein expression (Figure 7C). However, miR‐152‐3p had the opposite effect. miR‐152‐3p knockdown rescued the increase in oxidative stress and ROS production in H_2_O_2_‐treated HCs, whereas miR‐152‐3p inhibition promoted oxidative stress in HCs (Figure 7D,E,M).

The role of RNF219 in oxidative stress was similar to that of circGNB1 in HCs, although more pronounced. RNF219 expression down‐regulation significantly inhibited oxidative stress and reduced ROS production in HCs, even without H_2_O_2_ induction (Figure 7F,G,M), whereas overexpression of RNF219 alone promoted oxidative stress in untreated HCs (Figure 7H). Furthermore, RNF219 rescued the circGNB1‐ or mimicked miR‐152‐3p‐induced changes in COX2 and iNOS (Figure 7I). Finally, the role of CAV1 in oxidative stress was similar to that of RNF219 in potentiating oxidative stress in HCs (Figure 7J‐M). In summary, we conclude that the circGNB1/miR‐152‐3p/RNF219/CAV1 axis regulates oxidative stress in HCs and may affect OA progression.

### 3.9 The circGNB1/miR‐152‐3p/RNF219/CAV1 axis occurs via the IL‐10 signalling pathway

**FIGURE 8:**
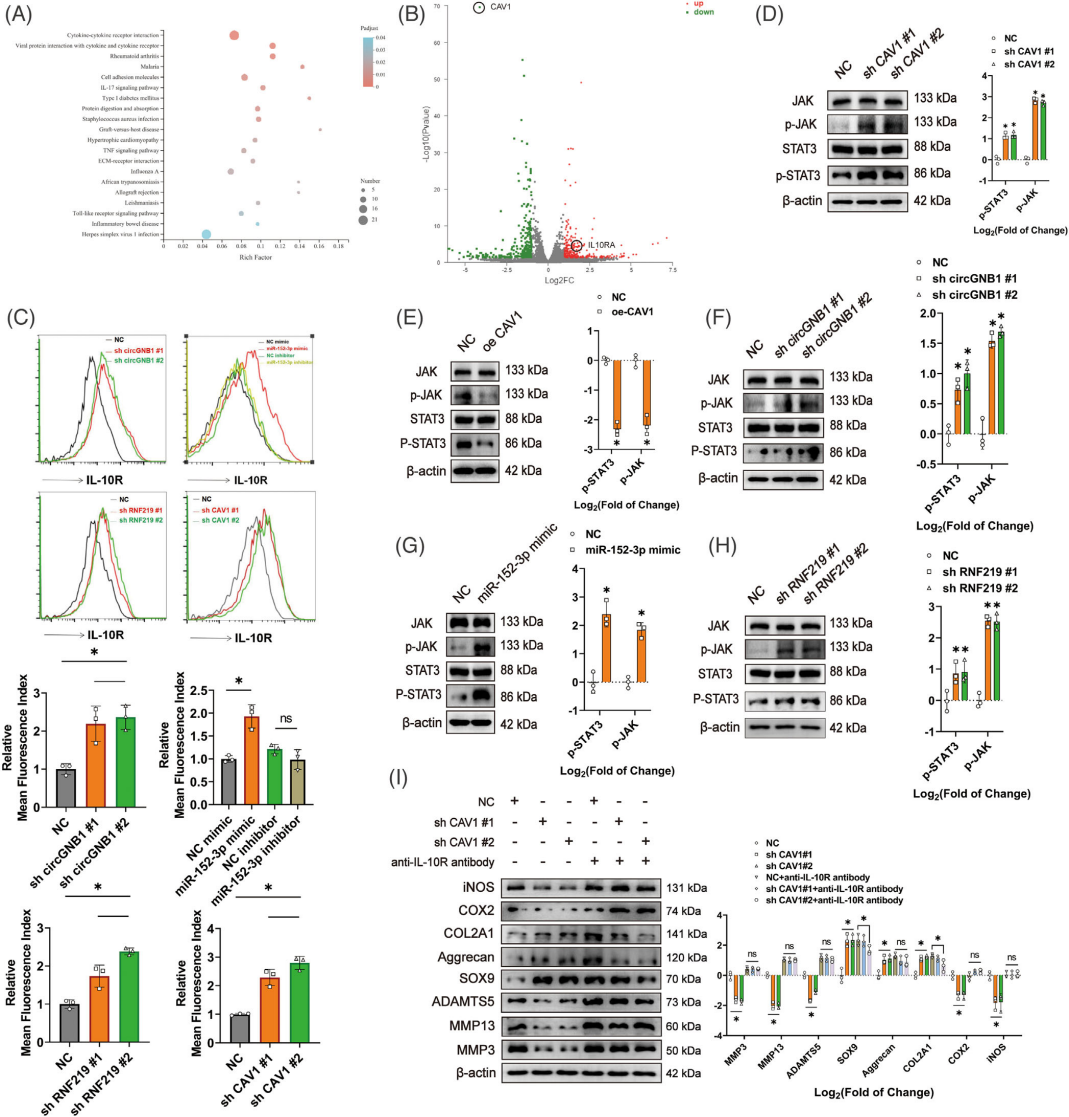
The circGNB1/miR‐152‐3p/RNF219/CAV1 axis signals through the IL‐10 signalling pathway. (A) Kyoto encyclopedia of genes and genomes (KEGG) pathway analysis of differentially expressed (up‐regulated and down‐regulated) genes in CAV1‐knockdown human chondrocytes (HCs). (*p* < .05). (B) Mean average plots comparing RNA‐seq data from HCs transfected with negative control (NC) or sh CAV1 (*n* = 3/group). Significantly (*n* = 3, *p* < .05) down‐regulated (in green) and up‐regulated (in red) genes for each sample set were shown. (C) Flow cytometric analysis of IL‐10R expression (as relative mean fluorescence index) in HCs overexpressing or deficient in circGNB1, miR‐152‐3p, RNF219, or CAV1. *n* = 3 (three different donors). *p < .05. (D) Left, western blots of phosphorylated STAT3 (p‐STAT3), phosphorylated JAK (p‐JAK), STAT3 and JAK in CAV1‐knockdown HCs. Right, quantification of fold‐change (log2) in protein bands density. *n* = 3 (three different donors). *p < .05. (E) Left, western blots of p‐STAT3, p‐JAK, STAT3 and JAK in CAV1‐overexpressing HCs. Right, quantification of fold‐change (log2) in protein bands density. *n* = 3 (three different donors). *p < .05. (F) Left, western blots of p‐STAT3, p‐JAK, STAT3 and JAK in circGNB1‐knockdown HCs. Right, quantification of fold‐change (log2) in protein bands density. *n* = 3 (three different donors). *p < .05. (G) Left, western blots of p‐STAT3, p‐JAK, STAT3 and JAK in HCs overexpressing miR‐152‐3p. Right, quantification of fold‐change (log2) in protein bands density. *n* = 3 (three different donors). *p < .05. (H) Left, western blots of p‐STAT3, p‐JAK, STAT3, JAK in HCs deficient in RNF219. Right, quantification of fold‐change (log2) in protein bands density. *n* = 3 (three different donors). *p < .05. (I) Left, western blots of ECM‐ and ROS‐associated proteins in CAV1‐knockdown HCs with or without anti‐IL‐10R antibody treatment. Right, quantification of fold‐change (log2) in protein bands density. *n* = 3 (three different donors). *p < .05. *p*‐Values are shown in graphs and were determined using Mann–Whitney *U* test (E and G) or Kruskal–Wallis test (C, D, F, H, and I). Data were presented as means ± standard deviation.

Next, we explored the signalling pathways participated in the circGNB1/miR‐152‐3p/RNF219/CAV1 axis. We examined the transcriptomes of CAV1‐knockdown and control HCs (*n* = 3) using RNA‐seq (Figure S5A‐D) and analyzed the top 20 differentially expressed gene function‐enriched signalling pathways using KEGG functional enrichment analysis (Figure 8A). We found that CAV1‐knockdown was most associated with the cytokine‐cytokine receptor interaction (Figure 8A). The differentially expressed genes (| log2 (fold change) | >1.5) involved in cytokine‐cytokine receptor interactions were analyzed using qRT‐PCR (Figure S5E) showed the most statistically significant up‐regulation of IL‐10RA expression in CAV1‐knockdown and circGNB1‐knockdown HCs (Figure 8B and Figure S5E). Given that many studies have shown that the IL‐10 signalling pathway is one of the most essential anti‐inflammatory signalling pathways,^31^, ^32^, ^33^ we speculated that the circGNB1/miR‐152‐3p/RNF219/CAV1 axis may mediate OA progression through the IL‐10 pathway. Using flow cytometric analysis, we found that down‐regulation of circGNB1, RNF219, and CAV1 and up‐regulation of miR‐152‐3p significantly up‐regulated IL‐10R expression in HCs (Figure 8C). Because IL‐10R plays its role through JAK/STAT3 pathway,^34^, ^35^ we assessed whether the circGNB1/miR‐152‐3p/RNF219/CAV1 axis affects JAK and STAT3 phosphorylation. Indeed, western blotting showed that CAV1 overexpression down‐regulated p‐JAK and p‐STAT3 (Figure 8E), while down‐regulation of CAV1, circGNB1, and RNF219 and overexpression of miR‐152‐3p led to elevated p‐JAK and p‐STAT3 levels (Figure 8D,F‐H). Using IL‐10R‐neutralizing antibodies, we showed that the regulatory effect of CAV1 on ECM and oxidative stress‐related proteins was inhibited (Figure 8I). In summary, we conclude that the circGNB1/miR‐152‐3p/RNF219/CAV1 axis regulates OA progression through the IL‐10 signalling pathway in HCs.

### 3.10 CircGNB1/miR‐152‐3p/RNF219/CAV1 axis promotes OA in vivo

**FIGURE 9:**
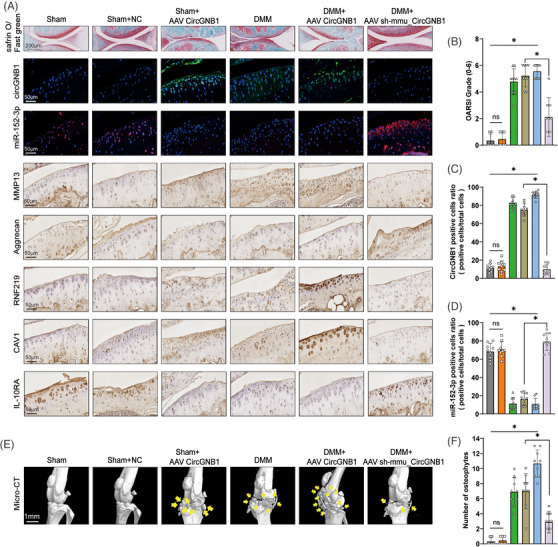
CircGNB1 promotes osteoarthritis (OA) in vivo. (A) Representative images of Safranin O/fast green, fluorescence in situ hybridization (FISH) staining of circGNB1, miR‐152‐3p and immunohistochemistry (IHC) staining of MMP13, Aggrecan, RNF219, CAV1, IL‐10RA in knee cartilage from mice overexpressing or deficient in circGNB1. Scale bars = 200 and 50 μm, respectively. (B) The OARSI grade knee joints in mice. *n* = 9 (nine different mice). *p < .05. (C) Quantification of CircGNB1 positive cells of CircGNB1 staining cartilage. *n* = 9 (nine different mice). *p < .05. (D) Quantification of miR‐152‐3p positive cells of miR‐152‐3p staining cartilage. *n* = 9 (nine different mice). *p < .05. (E) Representative three‐dimensional (3D) reconstruction micro‐CT images of mouse knee joints demonstrating aberrant osteophyte growth (yellow arrows). Scale bars, 1 mm. (F) Quantification of the number of osteophytes. *n* = 9 (nine different mice). *p < .05. *p*‐Values are shown in graphs and were determined using Kruskal–Wallis test (B and F) or one‐way ANOVA (C and D). Data were presented as means ± standard deviation.

We further investigated the in vivo roles of circGNB1 using the destabilization of the medial meniscus (DMM) mouse model of OA (Figure S6A) via intra‐articular injection of adeno‐associated viruses (AAV)‐overexpressing circGNB1 (AAV CircGNB1) or its shRNA (AAV‐sh‐mmu_CircGNB1). The efficiency of intra‐articular injection of the AAV overexpression system was confirmed by FISH staining (Figure 9A,C). Safranin O/fast green staining of proteoglycan and IHC staining of MMP13 and Aggrecan indicated that injection of AAV overexpressing circGNB1 into the mouse knee joint in the sham group (Sham+AAV CircGNB1) effectively induced the production of MMP13, leading to a decrease in Aggrecan and increased degradation of the cartilage matrix (Figure 9A). The detrimental effects of circGNB1 overexpression on cartilage were similar to those in the DMM group, and circGNB1 overexpression in the DMM group (DMM+AAV CircGNB1) further increased cartilage and joint degradation. In contrast, measured to the DMM group, knockdown of circGNB1 in the DMM group (DMM+AAV sh‐mmu_CircGNB1) suppressed MMP13 induction and rescued the degradation of Aggrecan and cartilage matrix (Figure 9A and Figure S6B,C). In addition, the OARSI scores also showed that no significant degeneration was seen in the Sham and Sham + NC groups, while the Sham+AAV CircGNB1 group, DMM group and DMM+AAV CircGNB1 group all produced significant pathological degeneration. In contrast, OA was rescued in the DMM+AAV sh‐mmu_CircGNB1 group compared to the DMM group (Figure 9B). Next, the function of the circGNB1/miR‐152‐3p/RNF219/CAV1/IL‐10RA axis in OA progression was confirmed by IHC staining (Figure 9A,D and Figure S6D–F).

Furthermore, micro‐CT scans showed that the knee joints in the Sham and Sham + NC groups were normal, whereas those in the Sham+AAV CircGNB1 group, DMM group and DMM+AAV CircGNB1 group had significantly more osteophytes, with most osteophytes observed in the DMM +AAV CircGNB1 group. However, by injecting AAV sh‐mmu_CircGNB1 into the DMM group, the number of osteophytes was significantly reduced compared to that in the DMM group (Figure 9E,F).

The overall outcomes indicate that circGNB1 promotes OA in vivo. Next, we used the same mouse DMM model to further validate the function of the downstream components of circGNB1 (miR‐152‐3p and RNF219) in vivo by intra‐articular injection of AAV overexpressing miR‐152‐3p (AAV miR‐152‐3p‐EGFP) or AAV RNF219 shRNA (AAV RNF219 shRNA‐EGFP), given that CAV1 has been shown to promote OA progression in vivo^27^ while IL‐10 is well known as an important anti‐inflammatory factor in OA.^36^ Safranin O/fast green staining of proteoglycans, IHC staining of MMP13 and Aggrecan, OARSI scoring, and micro‐CT scans showed that overexpression of miR‐152‐3p and knockdown of RNF219 rescued OA in vivo (Figure S6G‐M).

In summary, our data demonstrated that the circGNB1/miR‐152‐3p/RNF219/CAV1 axis promotes OA progression in vivo.

## 4 DISCUSSION

Abundant evidence has shown that OA pathogenesis is mainly caused by an imbalance between catabolism and anabolism.^37^ circRNAs can regulate cellular activities in a pleiotropic manner,^18^ and numerous studies have explored their role in OA initiation and progression.^38^, ^39^, ^40^ These findings indicated that circRNAs are promising diagnostic biomarkers and potential therapeutic targets in OA. Increasing evidence indicates a significant role of circRNAs in oxidative stress, apoptosis, and autophagy,^41^, ^42^, ^43^ all of which have important implications in OA. For example, circSERPINE2 could alleviate chondrocyte apoptosis and suppress the progression of OA.^41^ In addition, circRHOT1 suppressed chondrocyte autophagy by acting as a competing endogenous RNAs.^43^ However, few studies have focused on the relationships between circRNAs, ROS, senescence, and age‐related OA.

CircGNB1 is a potential therapeutic target and diagnostic biomarker of triple‐negative breast cancer.^44^ However, its role in OA progression remains unclear. Excessive intracellular ROS have a pro‐inflammatory effect on chondrocytes,^45^, ^46^ leading to a vicious cycle of OA pathogenesis. Here, we showed that circGNB1 affects ROS production in chondrocytes and is involved in OA development. Particularly, circGNB1 expression was up‐regulated in a dose‐dependent manner in H_2_O_2_‐induced chondrocytes. Meanwhile, it was also correspondingly up‐regulated in IL‐1β‐ and TNF‐α‐treated chondrocytes, and in the cartilage of medial tibial plateaus from older OA patients. Thus, circGNB1 expression positively correlated with chondrocyte inflammation and degeneration. Moreover, circGNB1 expression inhibition resulted in a significant reduction in intracellular ROS and broad inhibition of ECM degradation. Importantly, circGNB1 overexpression increased OA severity in the DMM mouse model, whereas circGNB1 silencing had the opposite effect.

RNF219 (also known as C13orf7) plays a role in prostate tumors,^47^ attention‐deficit/hyperactivity disorder^48^ and other diseases,^49^ although its role in OA remains unclear. In this article, our results indicated that RNF219 expression was up‐regulated in the cartilage of the medial tibial plateaus in older patients. Our data also confirmed RNF219's pro‐inflammatory effects on chondrocytes, as the knockdown of RNF219 resulted in a significant reduction in ROS and catabolic enzymes while enhancing the expression of anabolic enzymes.

This study also revealed the role of CAV1, a key caveolae component,^50^ in OA progression. The caveolin scaffolding domain of CAV1 enables it to operate as a scaffold protein and interact with a variety of molecules, which in turn mediate various processes, including signal transmission, caveolar homeostasis, and oncogenesis.^51^ Importantly, CAV1 overexpression induces senescence in age‐related diseases, and CAV1 is a senescence marker associated with oxidative stress in chondrocytes.^25^, ^26^ Our data show that CAV1 is up‐regulated in the cartilage of the medial tibial plateaus in older patients with OA and has a pro‐inflammatory effect on chondrocytes. Past studies have illustrated that the CAV1 gene promoter is activated through NF‐κB‐dependent and p38 MAPK‐Sp1‐dependent pathways under oxidative stress.^25^ Furthermore, studies have suggested a critical role for CAV1 in oxidative stress‐induced DNA damage repair and signalling.^26^ Therefore, a bidirectional regulatory relationship exists between oxidative stress and CAV1 expression.

A number of studies have reported that regulating E3 ubiquitin‐protein ligase plays a prominent role in RNF family proteins,^30^ but they can also function as negative regulators of the ubiquitin‐dependent signalling cascade.^52^, ^53^ For example, RNF169 interacts with double‐stranded DNA breaks and prevents RNF168 E3 ligase from docking, leading to the suppression of DNA damage‐induced ubiquitination.^52^ In this study, we showed that RNF219 affects CAV1 stability in an E3 ligand‐independent manner by binding to and preventing the proteasome‐mediated degradation of CAV1. Down‐regulation of RNF219 by silencing circGNB1 or overexpressing miR‐152‐3p promotes CAV1 ubiquitination and degradation. In addition, K47 was identified as a key ubiquitylation site in CAV1.

IL‐10 is an anti‐inflammatory cytokine and can potentially reestablish joint homeostasis.^54^, ^55^ In chondrocytes and the surrounding cartilage ECMs of patients with OA, IL‐10 and TNF levels are inversely related.^56^ Prior researches suggested that the expression of MMP1 and MMP13 genes is inhibited by IL‐10, suggesting that it is chondroprotective in human chondrocytes.^57^ In HCs deficient in CAV1, IL‐10RA was significantly up‐regulated. Moreover, RNA‐seq and KEGG pathway analyses implied that cytokine‐cytokine receptor interaction pathways were the top pathways affected by CAV1 silencing, suggesting that CAV1 may have a bearing on the progression of OA through the IL‐10 signalling pathway.

The expression of IL‐10RA, p‐JAK and p‐STAT3 was up‐regulated after silencing circGNB1, RNF219 and CAV1 or overexpressing miR‐152‐3p. These results demonstrated the activation of the IL‐10 signalling pathway in this system. Using an anti‐IL‐10R antibody, the regulatory effect of CAV1 on cartilage ECMs production was inhibited. Hence, we inferred that the circGNB1/miR‐152‐3p/RNF219/CAV1 axis is involved in OA progression via the IL‐10 signalling pathway.

**FIGURE 10:**
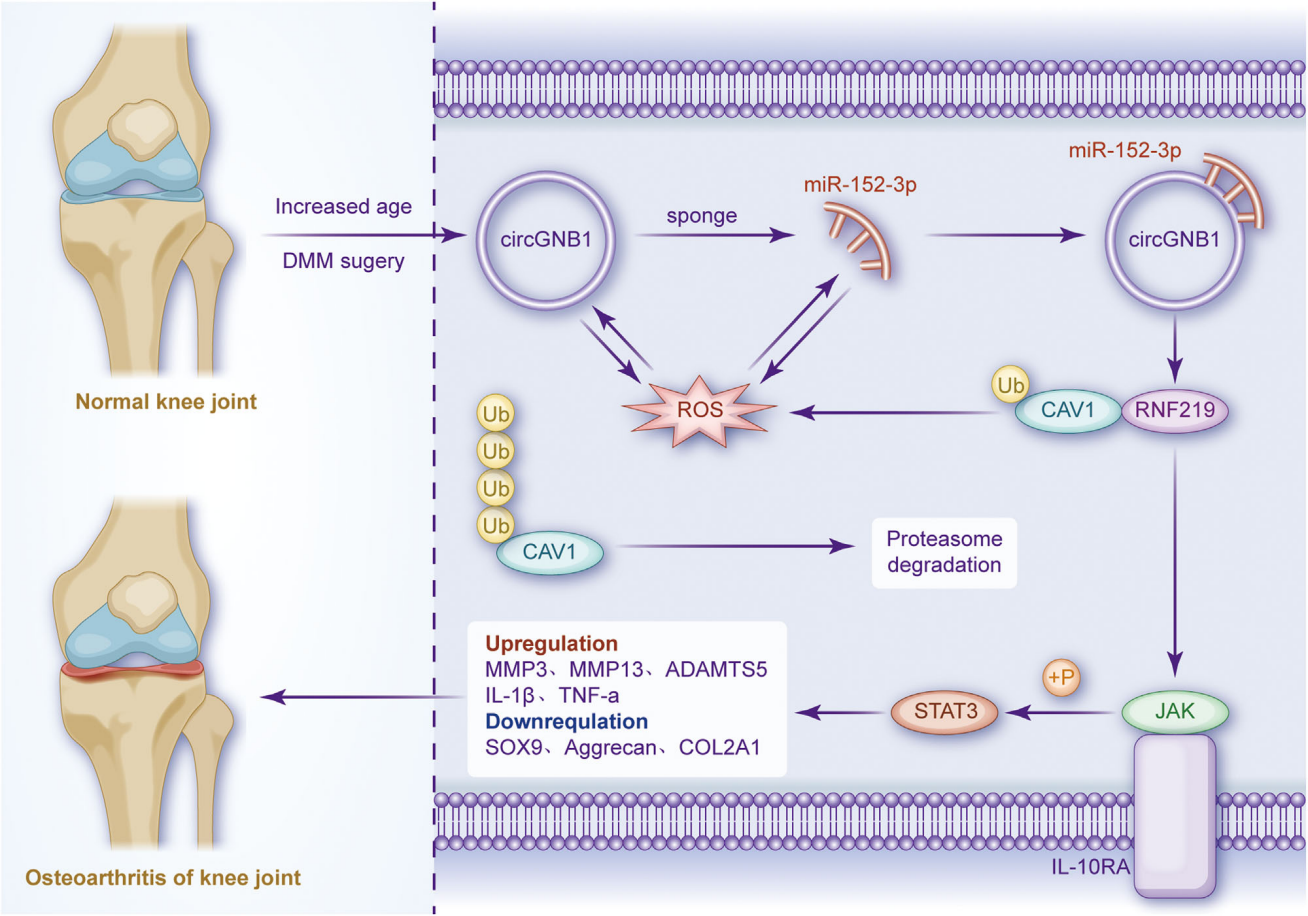
Schematic diagram depicting the mechanism of circGNB1 in promoting osteoarthritis (OA).

In summary, this study revealed a novel role for the circGNB1/miR‐152‐3p/RNF219/CAV1 axis in regulating oxidative stress, chondrocyte phenotypes and OA progression. Mechanistically, circGNB1 interferes with the miR‐152‐3p mRNA target RNF219, which regulates CAV1 ubiquitination and OA progression through the IL‐10 signalling pathway (Figure 10). Thus, the circGNB1/miR‐152‐3p/RNF219/CAV1 axis may serve as novel therapeutic targets and diagnostic markers for OA.

## CONFLICT OF INTEREST STATEMENT

The authors declare that they have no known competing financial interests or personal relationships exist.

## Supporting information

Supporting Information

Supporting Information

Supporting Information

Supporting Information

## Data Availability

All data supporting the findings of this study are available within the paper and its supplementary information.
