# Supplementary material for: CircGNB1 drives osteoarthritis pathogenesis by inducing oxidative stress in chondrocytes
Source: Clin Transl Med. 2023 Aug 3;13(8):e1358. doi: 10.1002/ctm2.1358 (PMC10400757; doi:10.1002/ctm2.1358)
Supplement: Supplementary file 1 — Supporting Information [file CTM2-13-e1358-s004.docx]

**CircGNB1 drives osteoarthritis pathogenesis by inducing oxidative stress in chondrocytes**

Yi Liang^#^, Lifeng Shen^#^, Weiyu Ni^#^, Yuhong Ding, Wentao Yang, Tianyuan Gu, Chenfeng Zhang, Jasper H. N. Yik, Dominik R. Haudenschild, Shunwu Fan, Shuying Shen, Ziang Hu

^#^These authors contributed equally to this work.

**Supplementary Figures**

**Supplementary Figure 1**

**
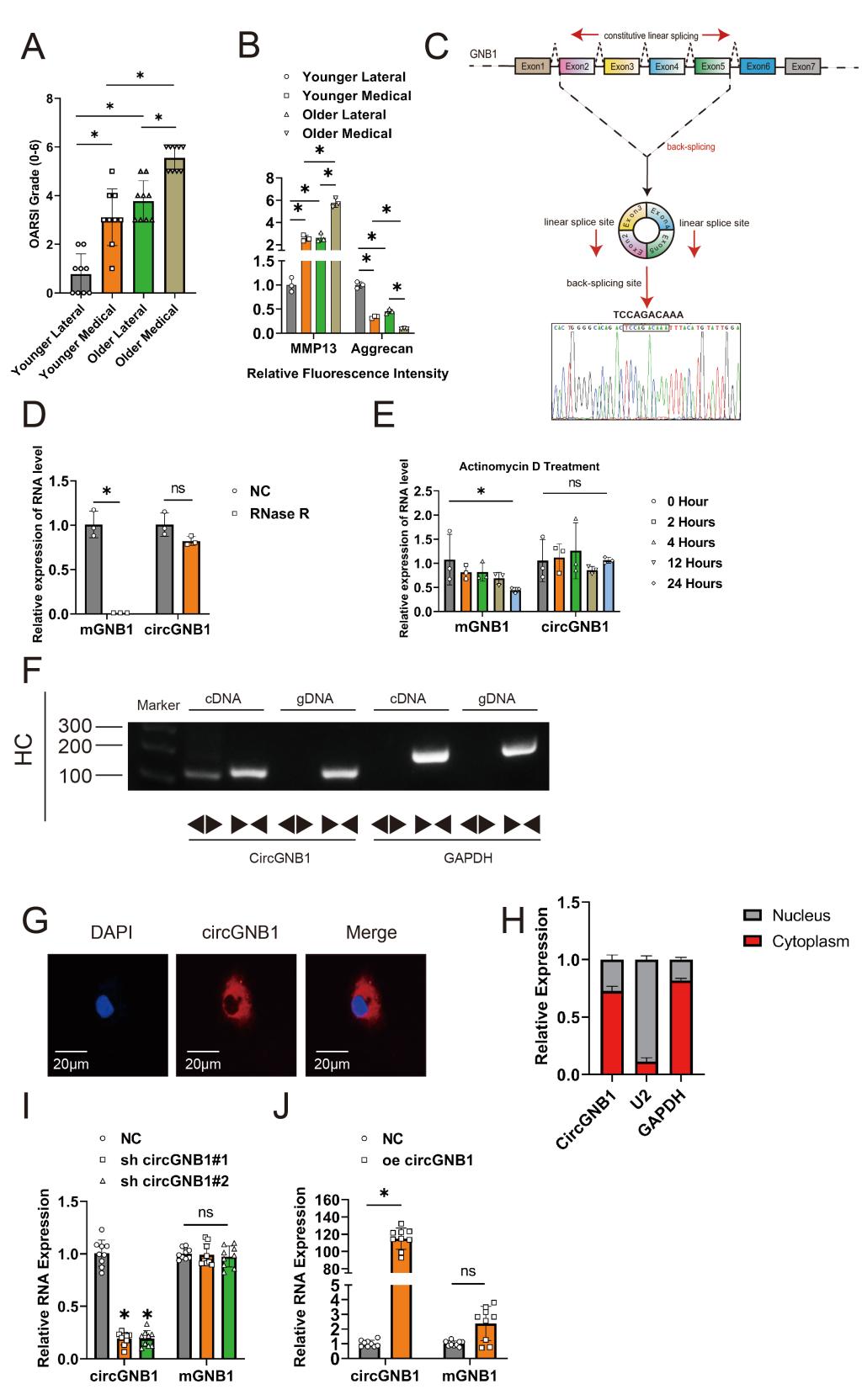
**

**Supplementary Figure 1: Identification of circGNB1 in HCs.** **(A)** The OARSI grade was used to evaluate histological alterations in human knee cartilage. (n=3). *p < 0.05. **(B)** Quantification of immunofluorescence with relative fluorescence intensity. n=3 (three different donors). *p < 0.05. **(C)** Annotated exon formation diagram for circGNB1; using the designated divergent flanking primers, sanger sequencing of PCR production was performed. **(D)** qRT-PCR was used to detect the expression of circGNB1 and mGNB1 in HCs after with or without RNase R treatment. n=3 (three different donors). *p < 0.05. **(E)** qRT-PCR was used to detect the expression of circGNB1 and mGNB1 in HCs after with or without Actinomycin D time-gradient treatment. n=3 (three different donors). *p < 0.05. **(F)** The existence of circGNB1 in HCs was confirmed, and circGNB1 was amplified in cDNA but not in gDNA using divergent primers. As a negative control, GAPDH was used. **(G)** Representative images of RNA FISH indicated that circGNB1 was primarily localized in the cytoplasm. Scale bars, 20 µm. **(H)** Nuclear cytoplasmic separation experiment analysis of circGNB1. **(I)** qRT-PCR was used to detect the expression of circGNB1 and mGNB1 in HCs after circGNB1 knockdown. n=3 (three different donors for three different experiments). *p < 0.05. **(J)** qRT-PCR was used to detect the expression of circGNB1 and mGNB1 in HCs after circGNB1 overexpression. n=3 (three different donors for three different experiments). *p < 0.05. P values are shown in graphs and were determined using Mann-Whitney U test(D), Kruskal-Wallis test(A,B,E), unpaired Student’s t-test(J) or one-way ANOVA(I). Data were presented as means ± standard deviation.

**Supplementary Figure 2**

**
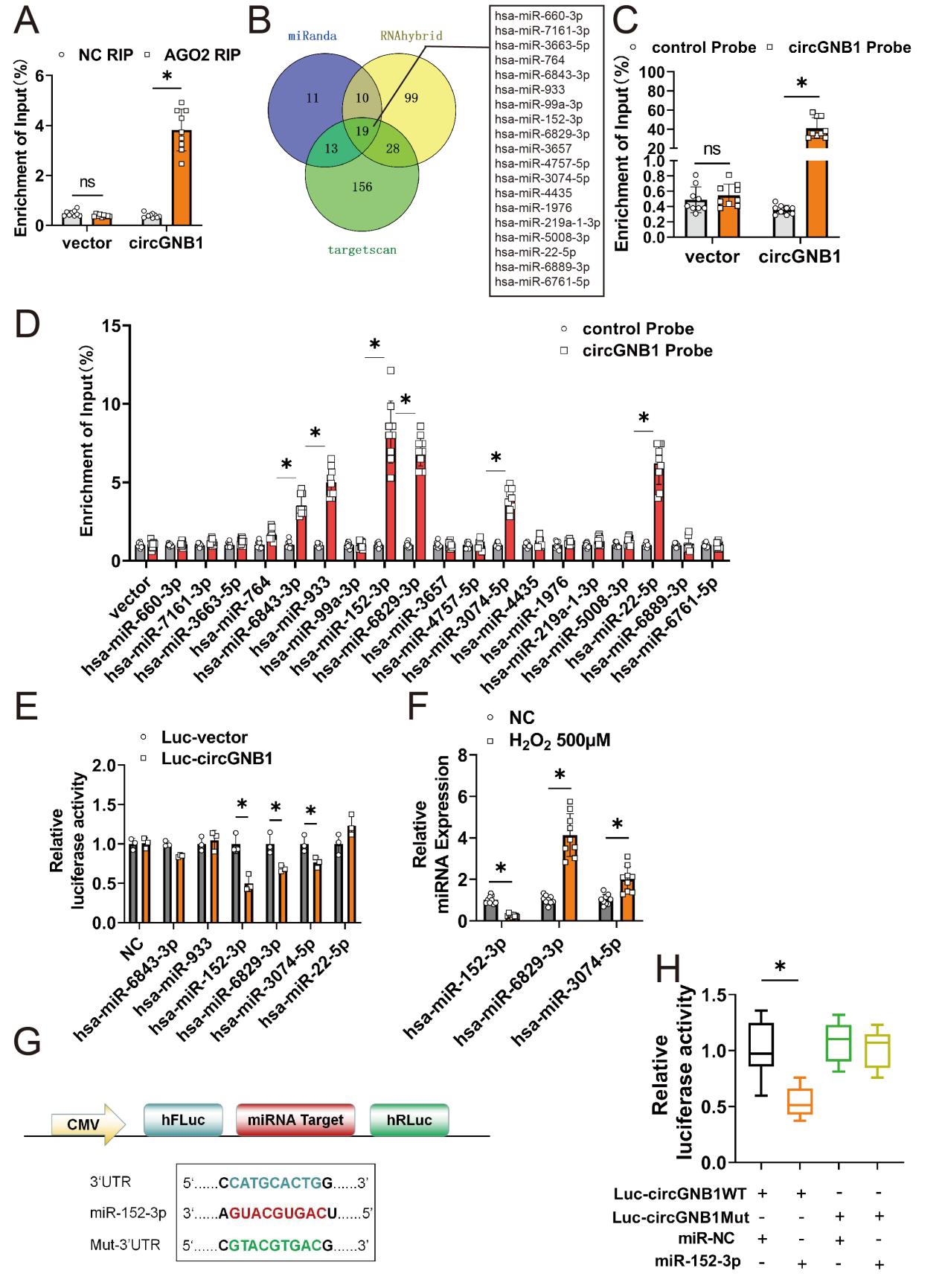
**

**Supplementary Figure 2: CircGNB1 efficiently sponges miR-152-3p. (A)** qRT-PCR quantification of argonaute-2 (AGO2)-bound circGNB1 using RIP. Input values were normalized to negative control (NC). n=3 (nine different experiments). *p < 0.05. **(B)** Venn diagram showed the 19 potential target miRNAs of circGNB1 identified through cross referencing the miRanda, RNAhybrid, and, TargetScan databases. **(C)** Quantification of the efficiency of the circGNB1 probe for RNA antisense purification (RAP) analysis using qRT-PCR. IP/ input values normalized to control probe. n=3 (three different donors for three different experiments). *p < 0.05. **(D)** Quantification of the circGNB1-bound miRNAs using qRT-PCR, IP/ input values were normalized to control probe. n=3 (three different donors for three different experiments). *p < 0.05. **(E)** Relative luciferase activity of the circGNB1 luciferase reporter plasmids in HEK-293T cells co-transfection with different candidate miRNA mimics. n=3 (three different experiments). *p < 0.05. **(F)** Quantification of candidate miRNAs relative expression using qRT-PCR in HCs stimulated by H_2_O_2_ (500 µM) for 48 h. n=3 (three different donors for three different experiments). *p < 0.05. **(G)** Dual-luciferase reporters (hFLuc-XbaL-hRLuc) with wild-type or mutant circGNB1 sequence in the XbaL region are shown schematically. **(H)** After co-transfection of dual-luciferase reporters (Luc-circGNB1 WT or Luc-GNB1 Mut) with miR-152-3p mimic or its control mimic into HEK-293T cells, relative luciferase activity was measured. n=3 (three different experiments). *p < 0.05. P values are shown in graphs and were determined using Mann-Whitney U test(E), Kruskal-Wallis test(H) or unpaired Student’s t-test(A,C,D,F). Data were presented as means ± standard deviation.

**Supplementary Figure 3**

**
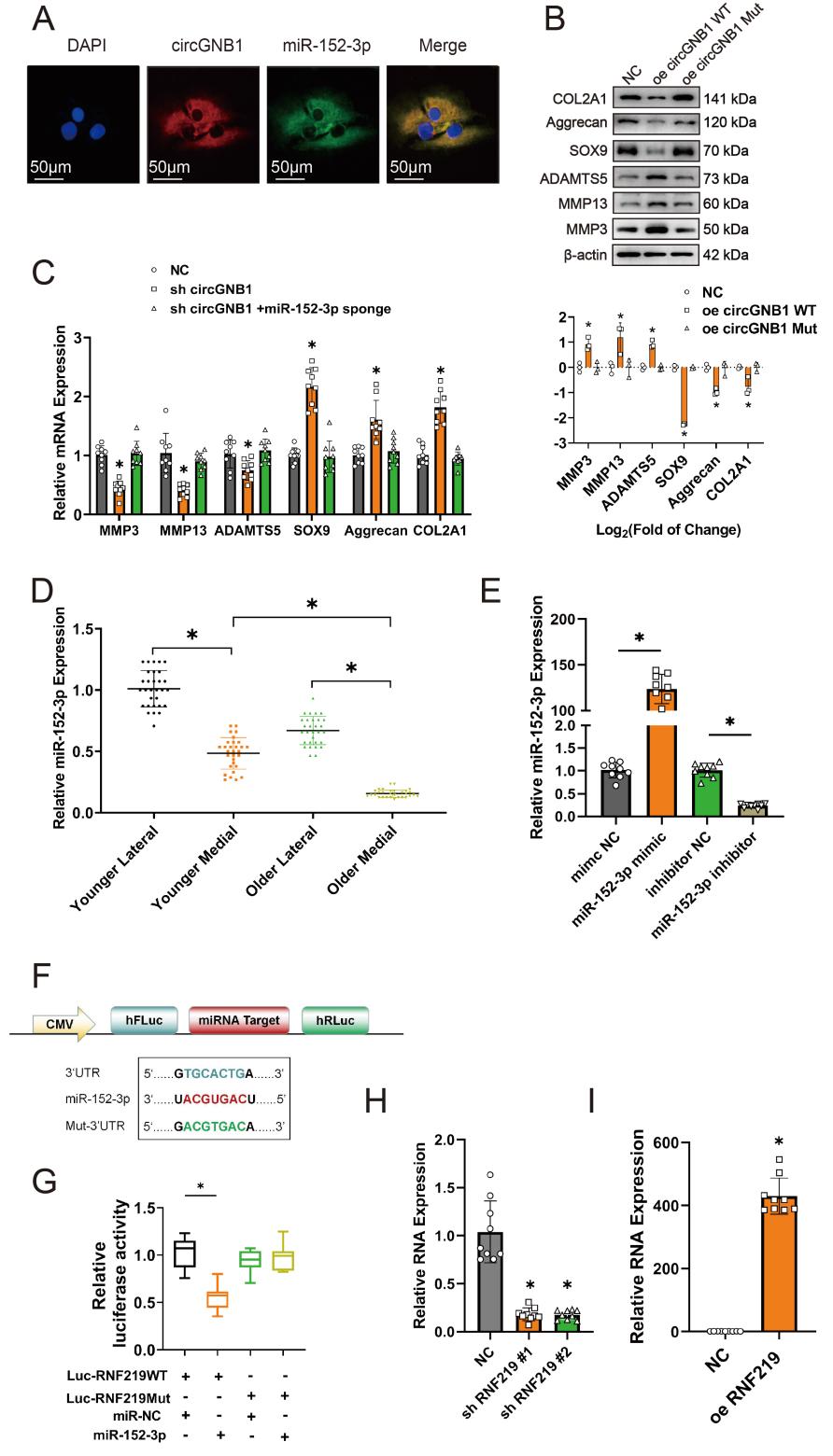
**

**Supplementary Figure 3: Knockdown and overexpression efficiency of miR-152-3p, RNF219. (A)** Representative images of FISH staining showing co-localization of circGNB1(red) and miR-152-3p (green). Scale bars, 50 µm. **(B)** Upper, western blots of extracellular matrix (ECM) associated proteins in cells overexpressing wildtype circGNB1 or its mutant defective in miR-152-3p binding. Lower, quantification of fold change in protein band density. n=3 (three different donors). *p < 0.05. **(C)** mRNA expression of MMP3, MMP13, ADAMTS5, SOX9, Aggrecan and COL2A1 in HC cells was detected by qRT-PCR analysis. Cells were infected with control virus or sh circGNB1 with or without miR-152-3p sponge adenovirus. n=3 (three different donors for three different experiments). *p<0.05. **(D)** qRT-PCR quantification of miR-152-3p relative expression in HCs from different donors. n=10 (ten different donors for three different experiments). *p < 0.05. **(E)** qRT-PCR was used to detect the expression of miR-152-3p in HCs after intervention for miR-152-3p. n=3 (three different donors for three different experiments). *p < 0.05. **(F)** Dual-luciferase reporters (hFLuc-XbaL-hRLuc) with wild-type or mutant RNF219 sequence in the XbaL region are shown schematically. **(G)** After co-transfection of dual-luciferase reporters (Luc-RNF219 WT or Luc-RNF219 Mut) with miR-152-3p mimic or its control mimic into HEK-293T cells, relative luciferase activity was measured. n=3 (three different experiments). *p < 0.05. **(H)** qRT-PCR was used to detect the mRNA expression of RNF219 in HCs after RNF219 knockdown. n=3 (three different donors for three different experiments). *p < 0.05. **(I)** qRT-PCR was used to detect the mRNA expression of RNF219 in HCs after RNF219 overexpression. n=3 (three different donors for three different experiments). *p < 0.05. P values are shown in graphs and were determined using Kruskal-Wallis test(B,G), unpaired Student’s t-test(E,I) or one-way ANOVA(C,D,H). Data were presented as means ± standard deviation.

**Supplementary Figure 4**

**
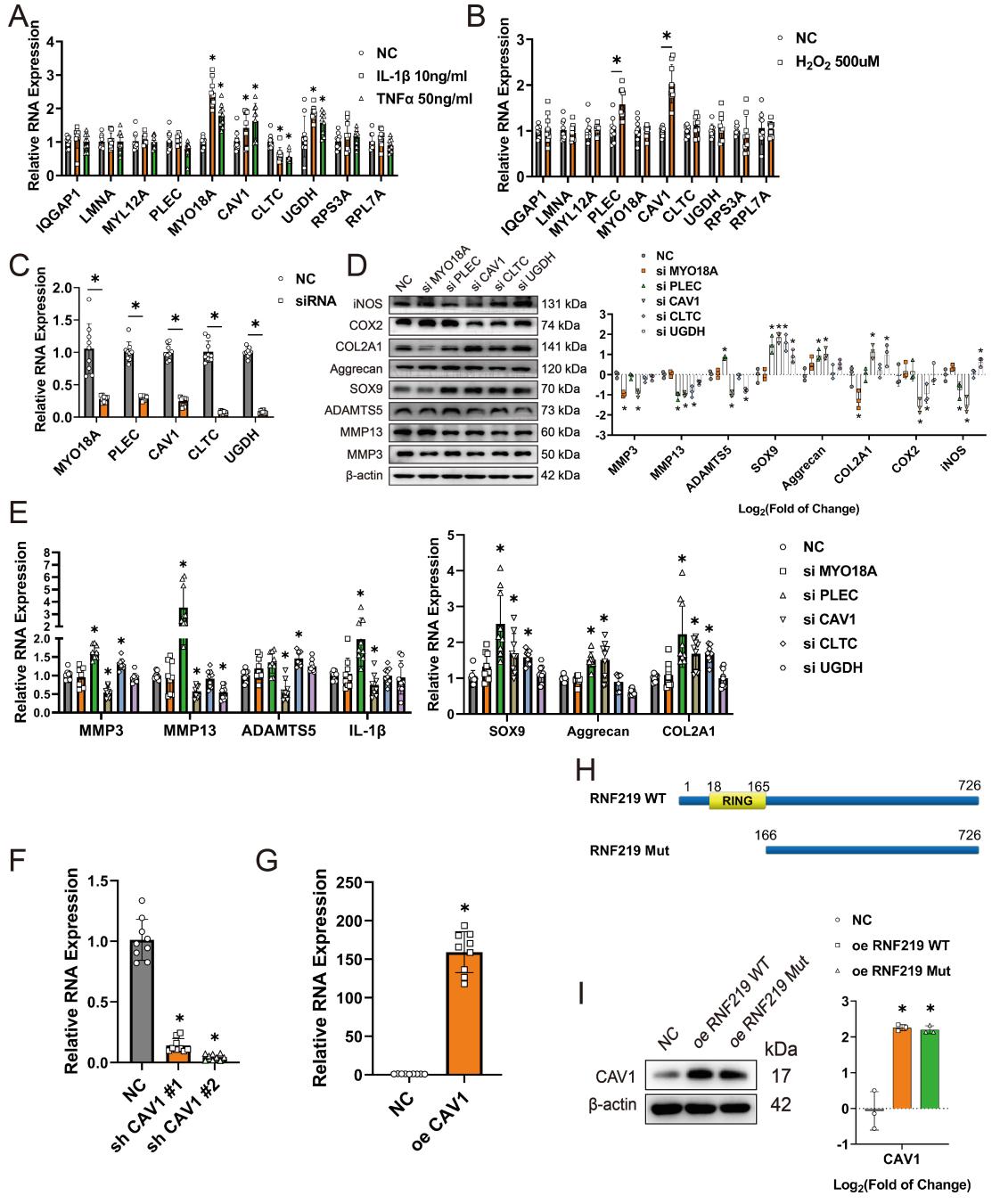
**

**Supplementary Figure 4: CAV1 is selected as the downsteam gene involved in the progression of OA in HCs. (A)** Quantification of the candidate mRNA relative expression using qRT-PCR in HCs stimulated by IL-1β (10 ng/mL) or TNF-α (50 ng/mL) for 48 h. n=3 (three different donors for three different experiments). *p < 0.05. **(B)** Quantification of the candidate mRNA relative expression using qRT-PCR in HCs stimulated by H_2_O_2_ (500 µM) for 48 h. n=3 (three different donors for three different experiments). *p < 0.05. **(C)** Quantification of the candidate mRNA relative expression using qRT-PCR in HCs after candidate genes knockdown. n=3 (three different donors for three different experiments). *p < 0.05. **(D)** Left,after candidate genes knockdown, western blot experiments of extracellular matrix (ECM) associated proteins and reactive oxygen species (ROS) associated pro-inflammatory protein were performed. Right, using log2(fold of change) to quantify western blot experiments. n=3 (three different donors). *p < 0.05. **(E)** Quantification of relative RNA levels associated with catabolic enzymes, pro-inflammatory cytokines, synthetase and proteoglycans using qRT-PCR after candidate genes knockdown. n=3 (three different donors for three different experiments). *p < 0.05. **(F)** qRT-PCR was used to detect the mRNA expression of CAV1 in HCs after CAV1 knockdown. n=3 (three different donors for three different experiments). *p < 0.05. **(G)** qRT-PCR was used to detect the mRNA expression of CAV1 in HCs after CAV1 overexpression. n=3 (three different donors for three different experiments). *p < 0.05. **(H)** Schematic illustration of constructs for RNF219. **(I)** Left, western blots of CAV1 proteins in cells overexpressing wildtype RNF219 or its mutant defective in the RING domain. Right, quantification of fold change in protein band density. n=3 (three different donors). *p < 0.05. P values are shown in graphs and were determined using Kruskal-Wallis test(D,I), unpaired Student’s t-test(B,C,G) or one-way ANOVA(A,E,F). Data were presented as means ± standard deviation.

**Supplementary Figure 5**

**
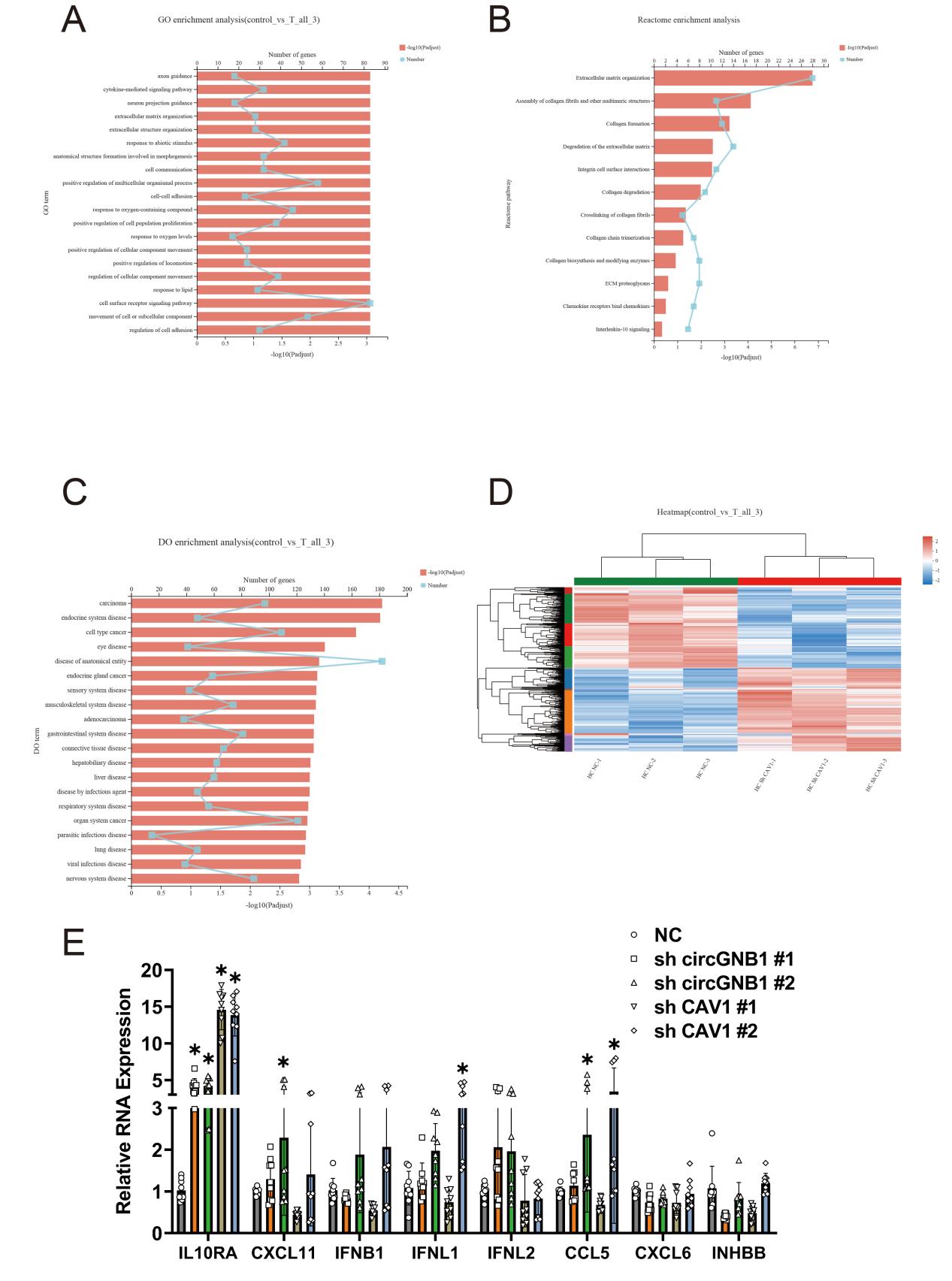
**

**Supplementary Figure 5: Schematic diagram of differentially expressed genes analysis. (A)** GO enrichment analysis, **(B)** Reactome enrichment analysis, **(C)** DO enrichment analysis and **(D)** heatmap of the differentially expressed (upregulated and downregulated) genes between NC and sh CAV1 (P < 0.05). **(E)** Quantification of the candidate mRNA (| log2 (fold change) | > 1.5) relative expression using qRT-PCR in CAV1-knockdown and circGNB1-knockdown HCs. n=3 (three different donors for three different experiments). *p < 0.05. P values are shown in graphs and were determined using one-way ANOVA(E). Data were presented as means ± standard deviation.

**Supplementary Figure 6**

**
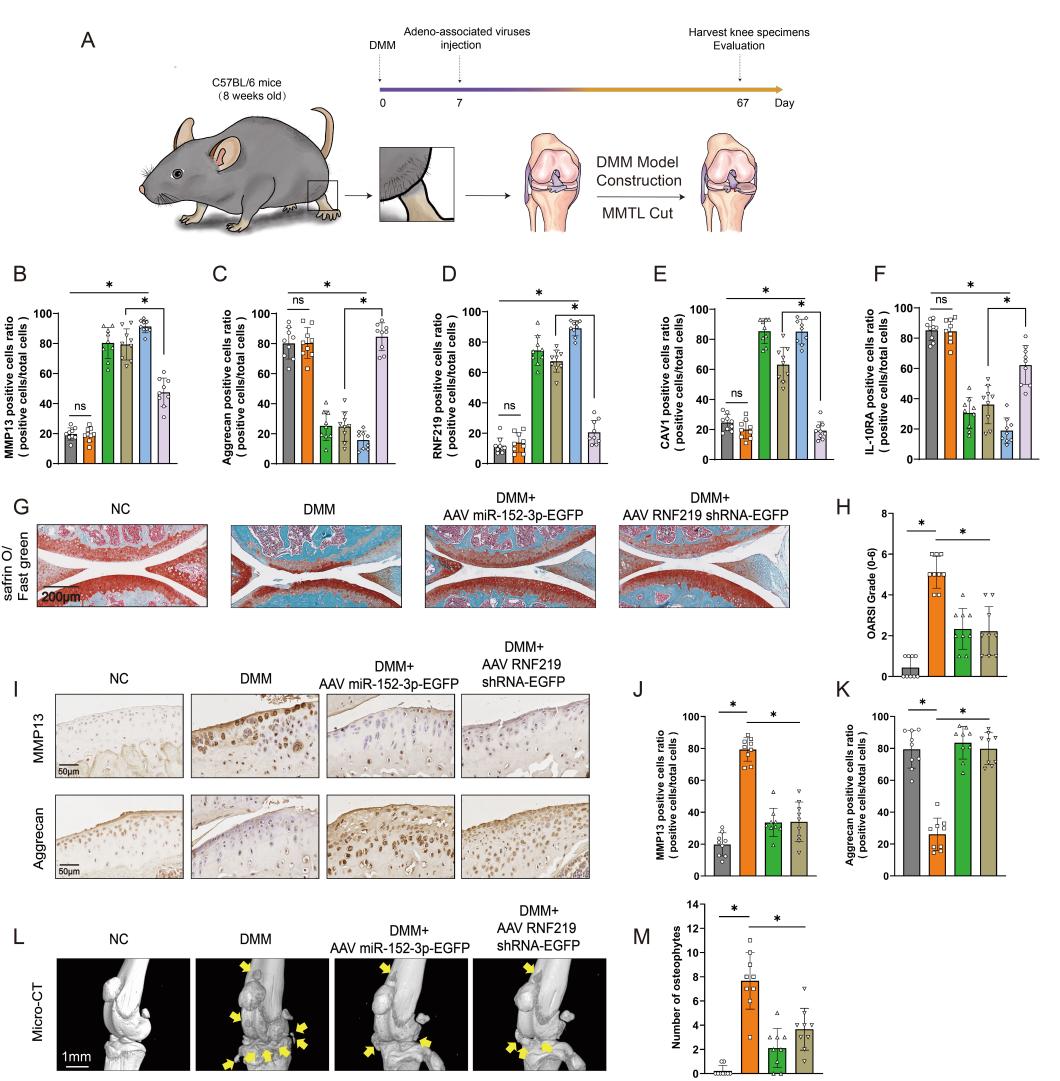
**

**Supplementary Figure 6: CircGNB1/miR-152-3p/RNF219/CAV1 axis promotes OA in vivo. (A)** Schematic diagram of DMM mouse model. **(B)** Quantification of MMP13 positive cells of MMP13 staining cartilage. n=9 (nine different mice). *p < 0.05. **(C)** Quantification of Aggrecan positive cells of Aggrecan staining cartilage. n=9 (nine different mice). *p < 0.05. **(D)** Quantification of RNF219 positive cells of RNF219 staining cartilage. n=9 (nine different mice). *p < 0.05. **(E)** Quantification of CAV1 positive cells of CAV1 staining cartilage. n=9 (nine different mice). *p < 0.05. **(F)** Quantification of IL-10RA positive cells of IL-10RA staining cartilage. n=9 (nine different mice). *p < 0.05. **(G)** Representative images of Safranin O/ Fast green in knee cartilage from mice overexpressing miR-152-3p or deficient in RNF219. Scale bars = 200 µm, respectively. **(H)** The OARSI grade knee joints in mice. n=9 (nine different mice). *p < 0.05. **(I)** Representative images of IHC staining of MMP13 and Aggrecan in knee cartilage from mice overexpressing miR-152-3p or deficient in RNF219. Scale bars = 50 µm, respectively. **(J)** Quantification of MMP13 positive cells of MMP13 staining cartilage. n=9 (nine different mice). *p < 0.05. **(K)** Quantification of Aggrecan positive cells of Aggrecan staining cartilage. n=9 (nine different mice). *p < 0.05. **(L)** Representative three-dimensional (3D) reconstruction micro-CT images of mouse knee joints demonstrating aberrant osteophyte growth (yellow arrows). Scale bars, 1 mm. **(M)** Quantification of the number of osteophytes. n=9 (nine different mice). *p < 0.05. P values are shown in graphs and were determined using Kruskal-Wallis test(H,M) or one-way ANOVA(B,C,D,E,F,J,K). Data were presented as means ± standard deviation.

**Supplementary Figure 7**

**
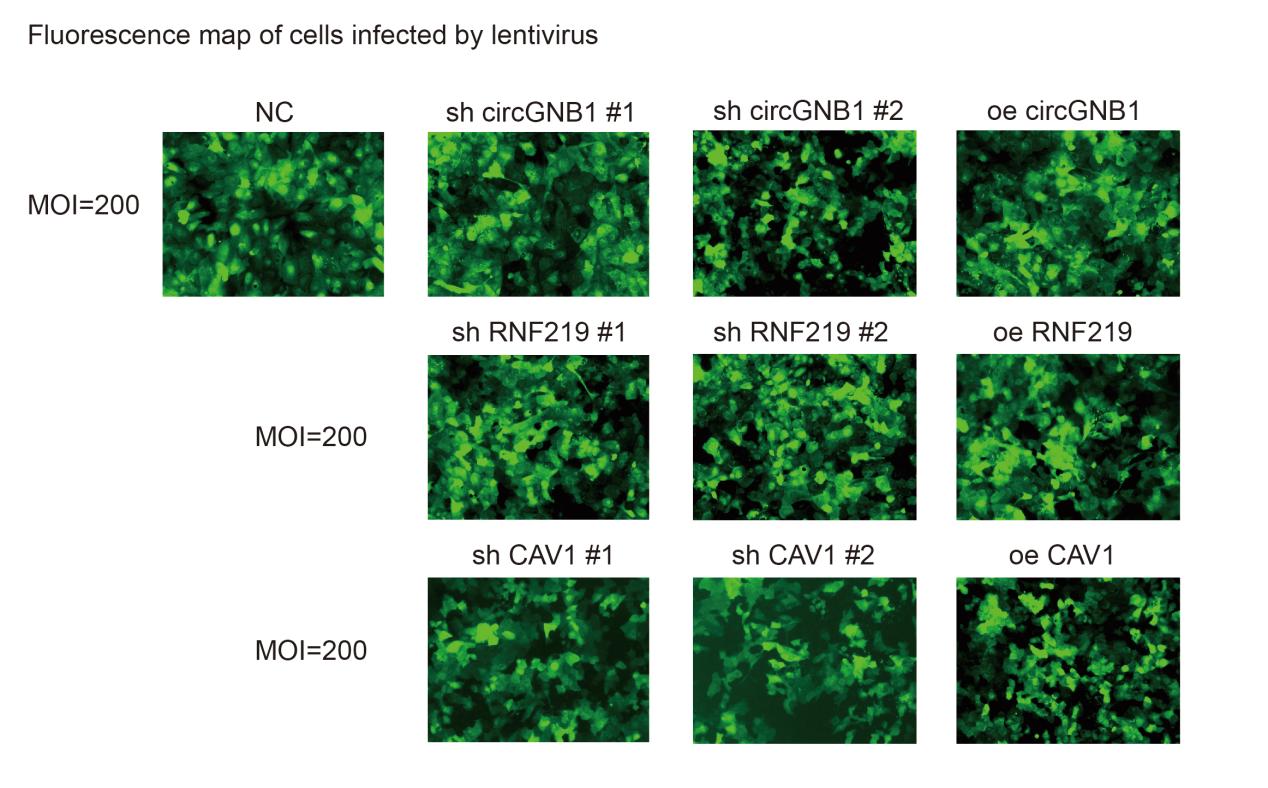
**

**Supplementary Figure 7: Fluorescence map of cells infected by lentivirus.**
