# Supplementary material for: CircGNB1 drives osteoarthritis pathogenesis by inducing oxidative stress in chondrocytes
Source: Clin Transl Med. 2023 Aug 3;13(8):e1358. doi: 10.1002/ctm2.1358 (PMC10400757; doi:10.1002/ctm2.1358)
Supplement: Supplementary file 2 — Supporting Information [file CTM2-13-e1358-s003.docx]

**Figure1D**

**
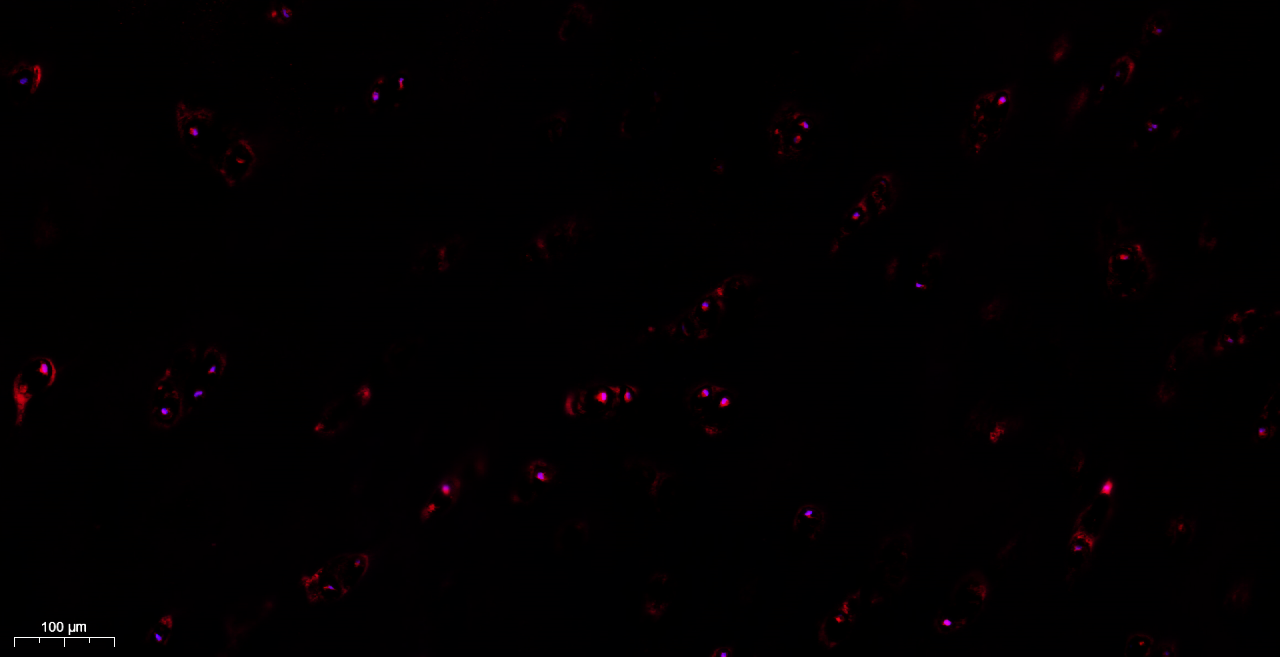
**

older lateral aggrecan


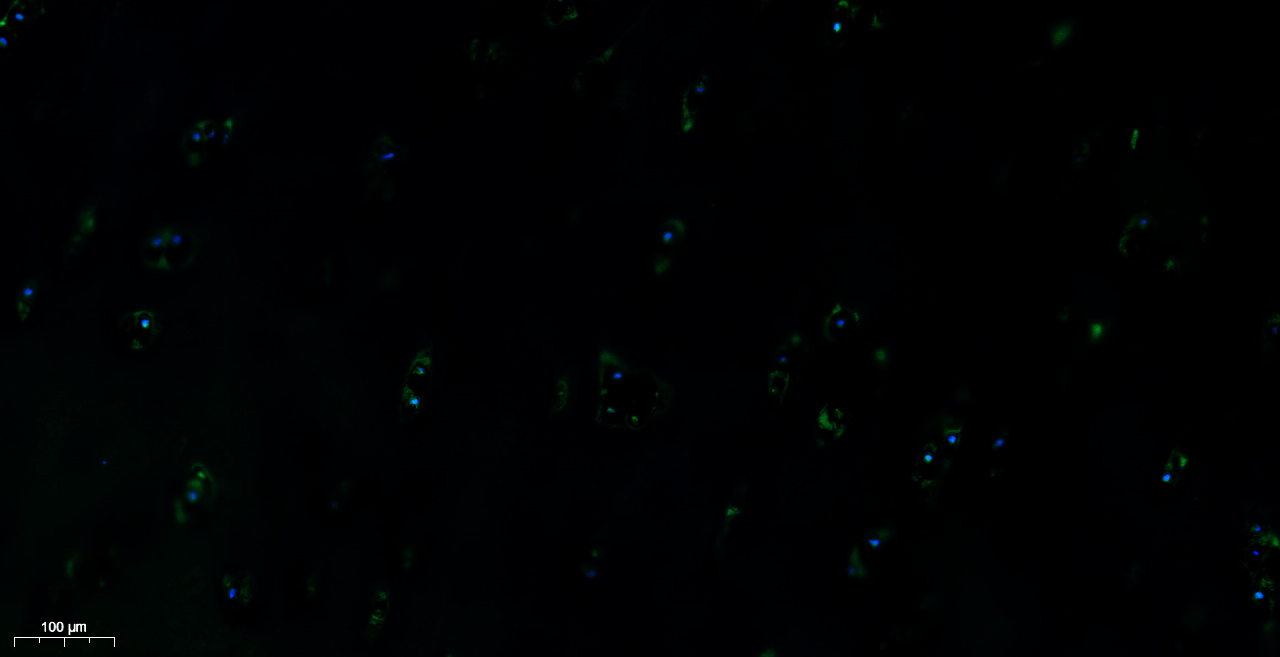


older lateral MMP13


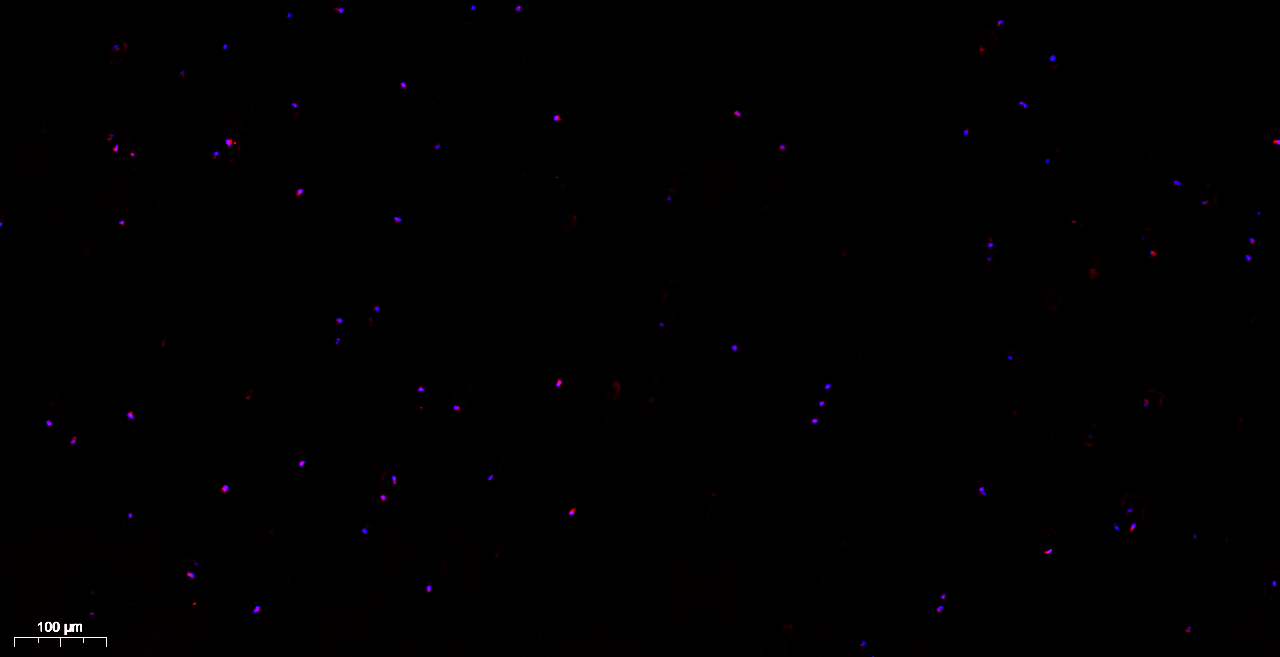


older medial aggrecan


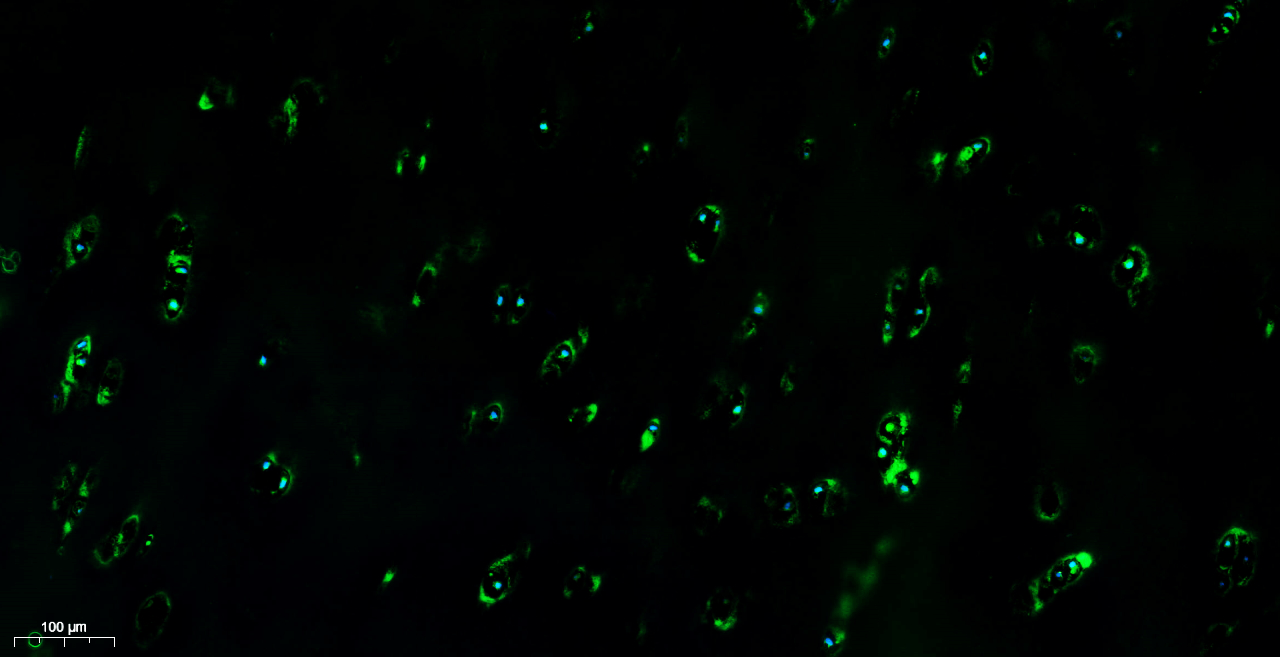


older medical MMP13


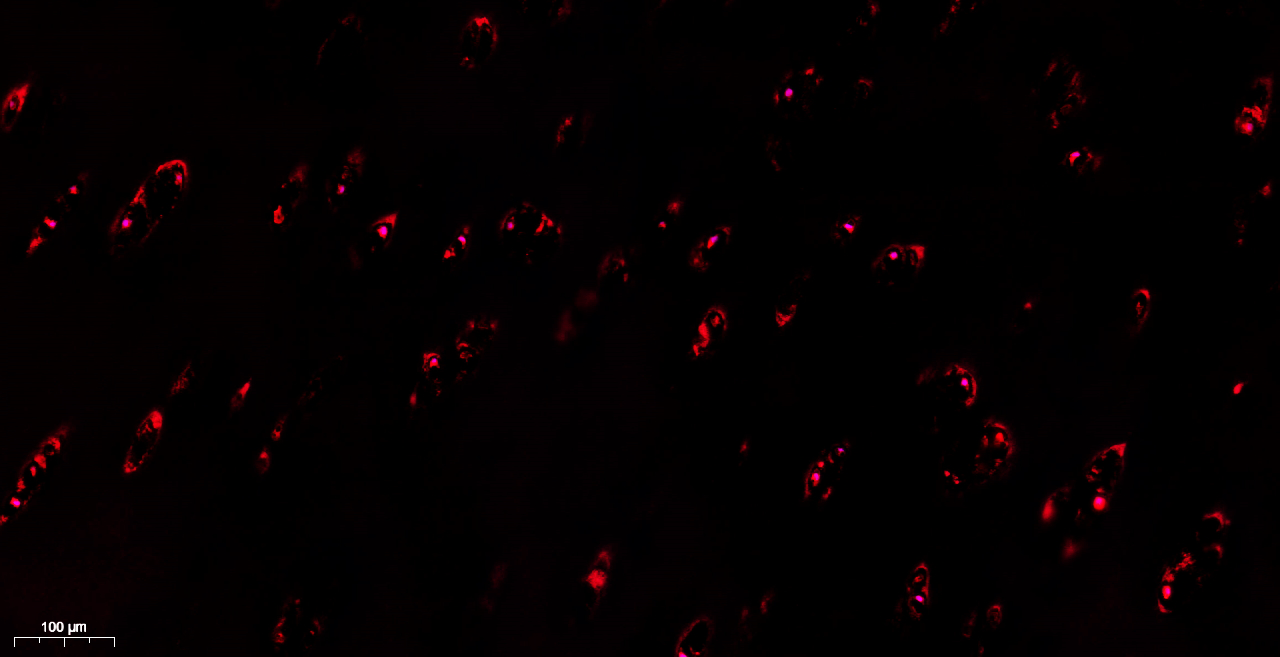


younger lateral aggrecan


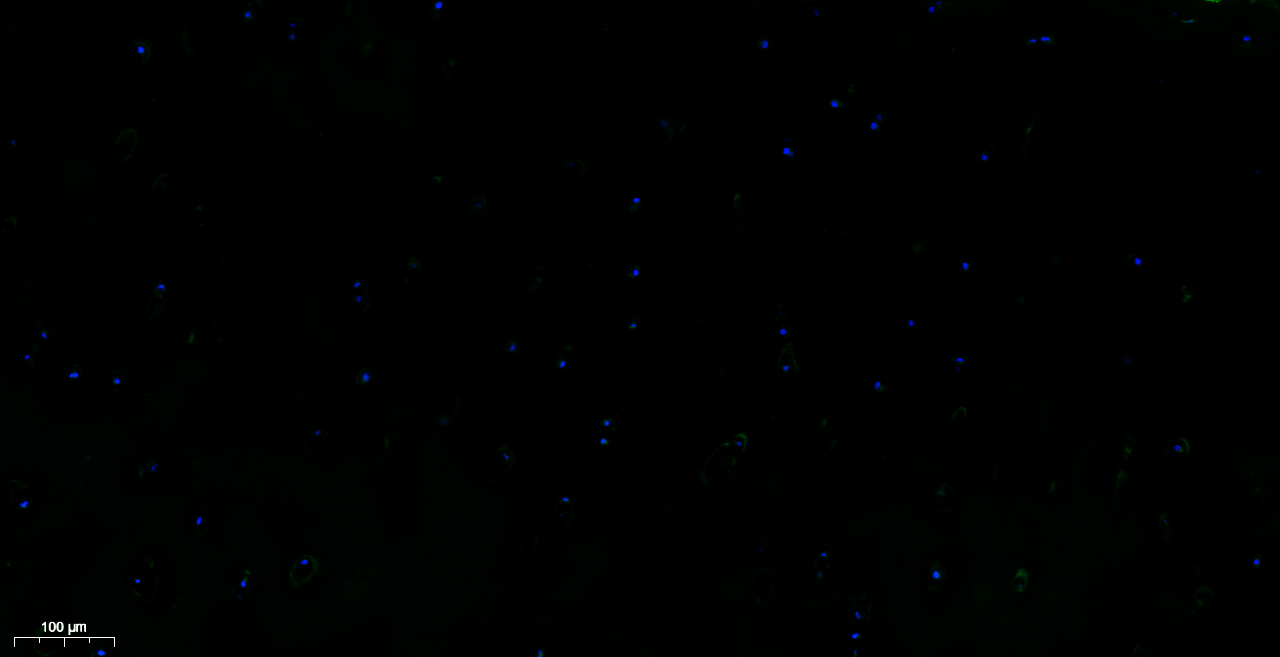


younger lateral MMP13


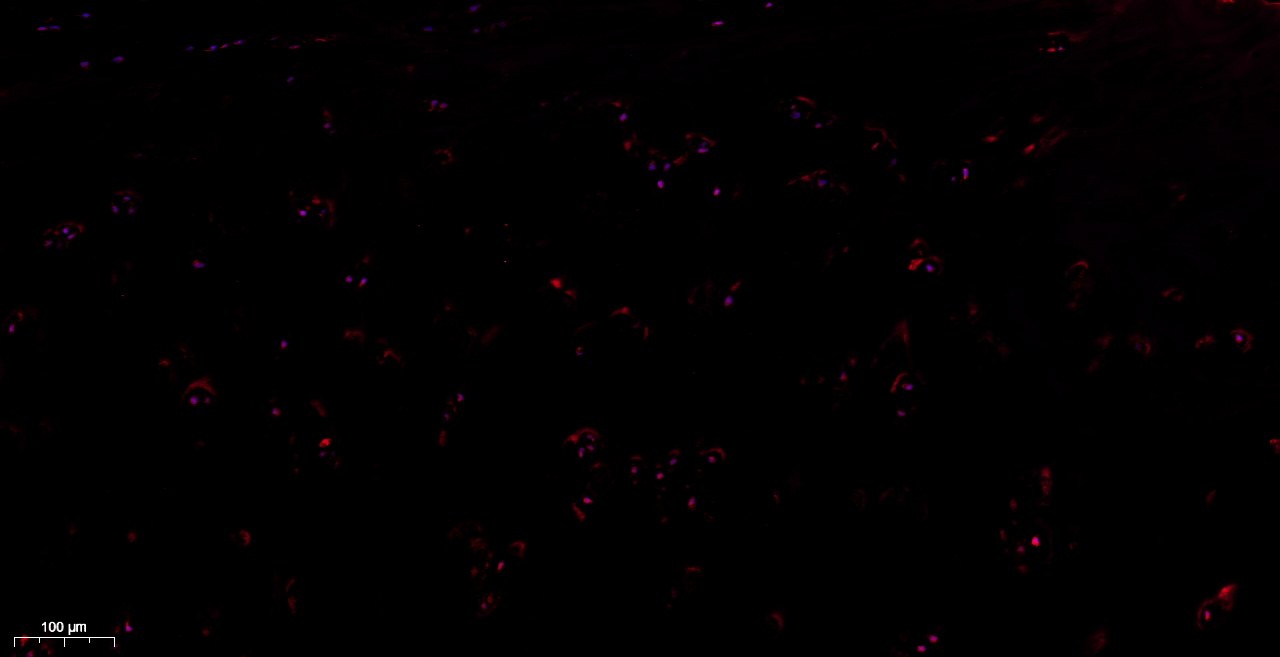


younger medial aggrecan


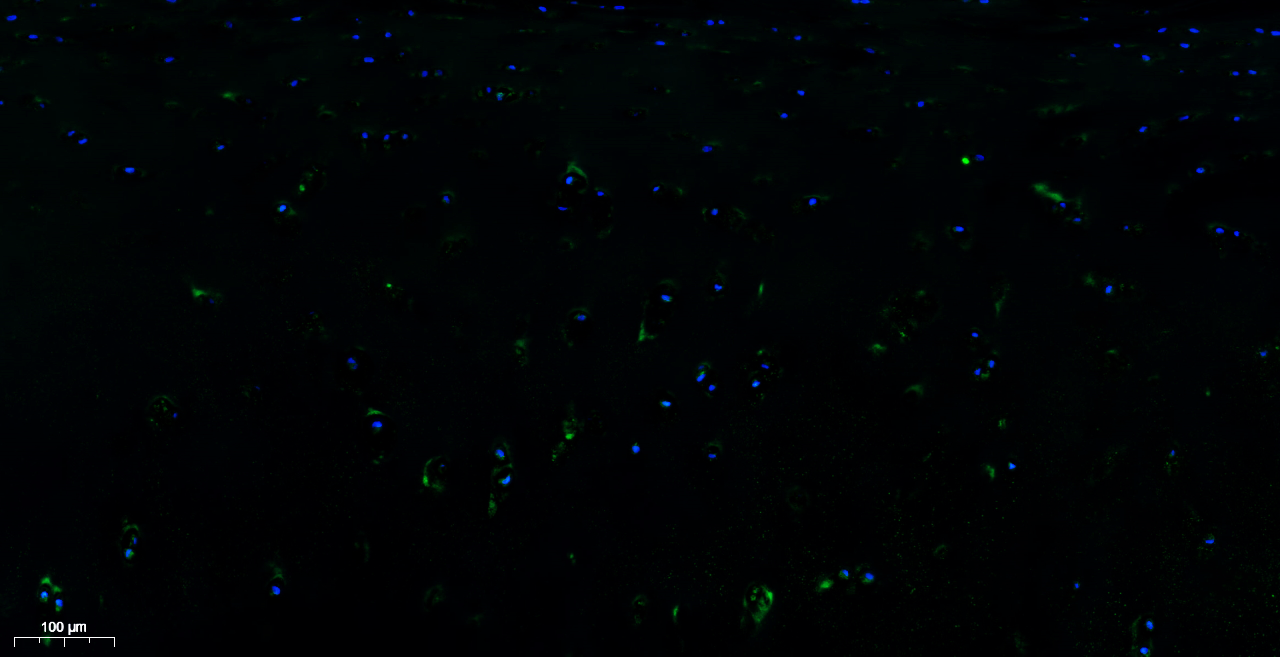


younger medical MMP13

**Figure2C**

**
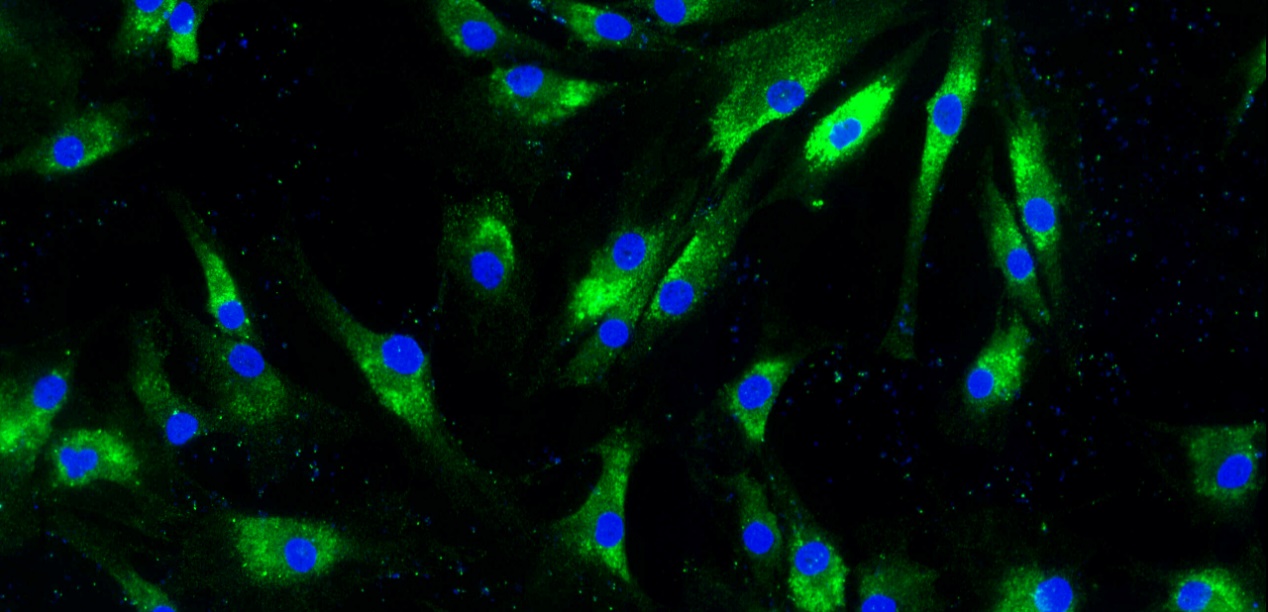
**

NC MMP13


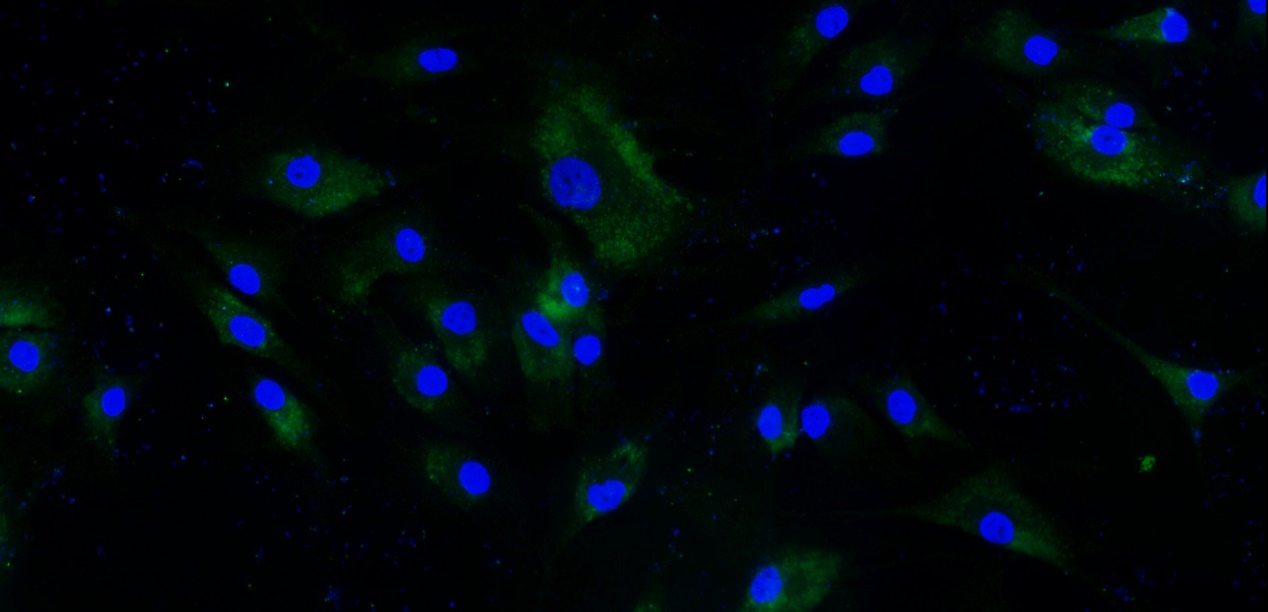


sh circGNB1#1 MMP13


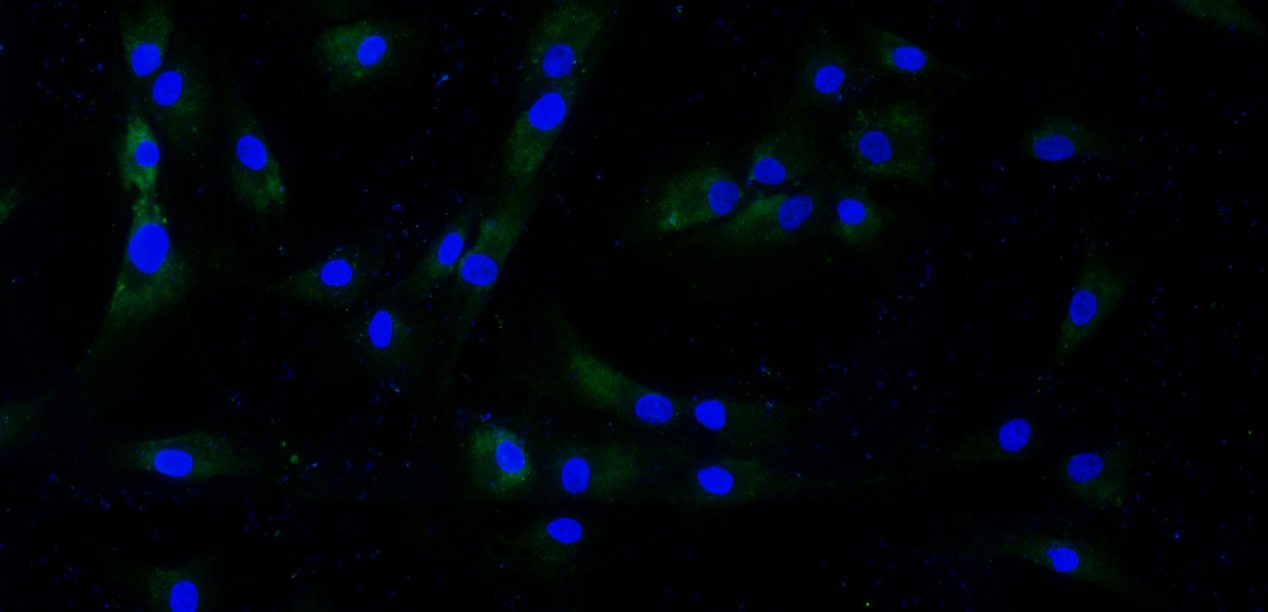


sh circGNB1#2 MMP13


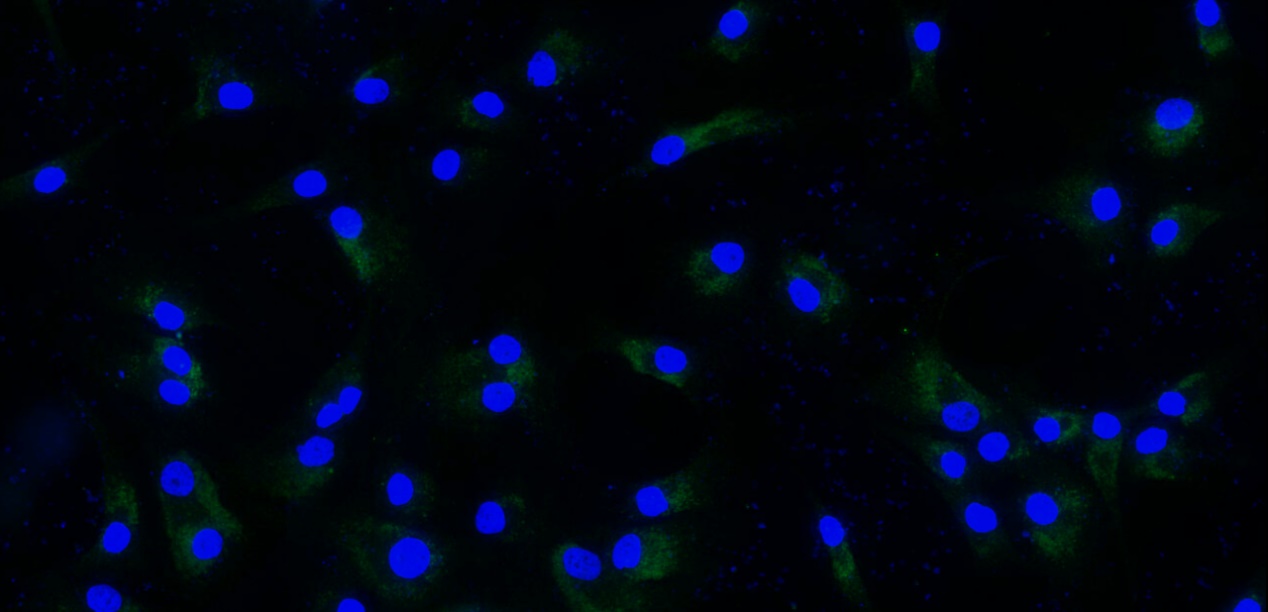


NC COL2A1


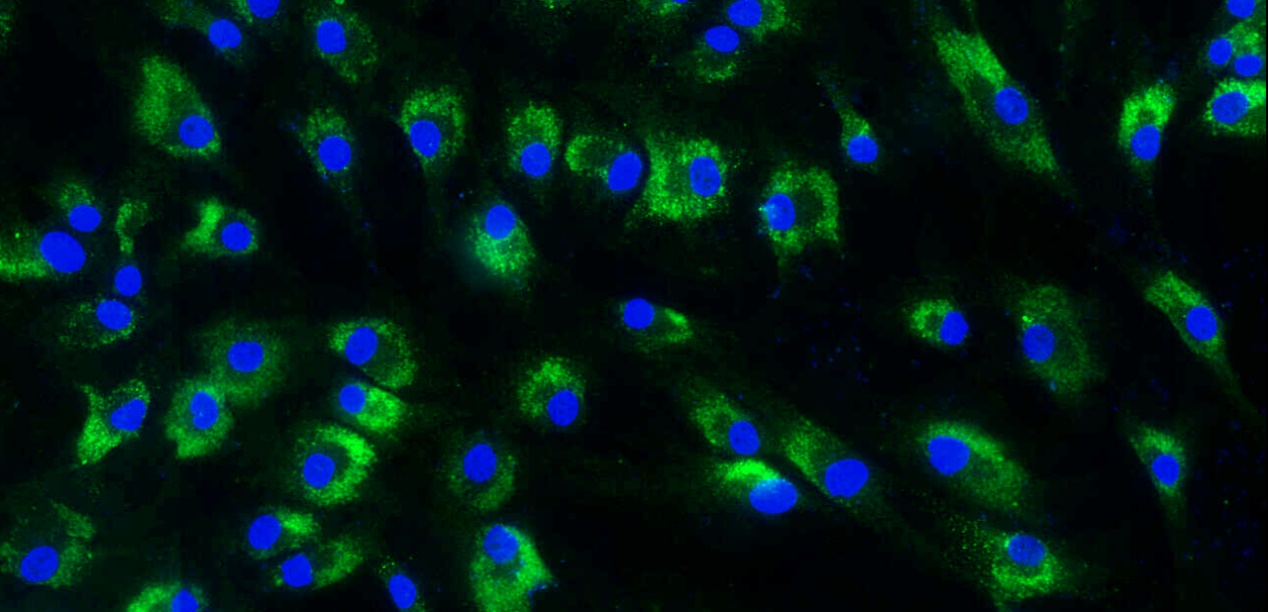


sh circGNB1#1 COL2A1


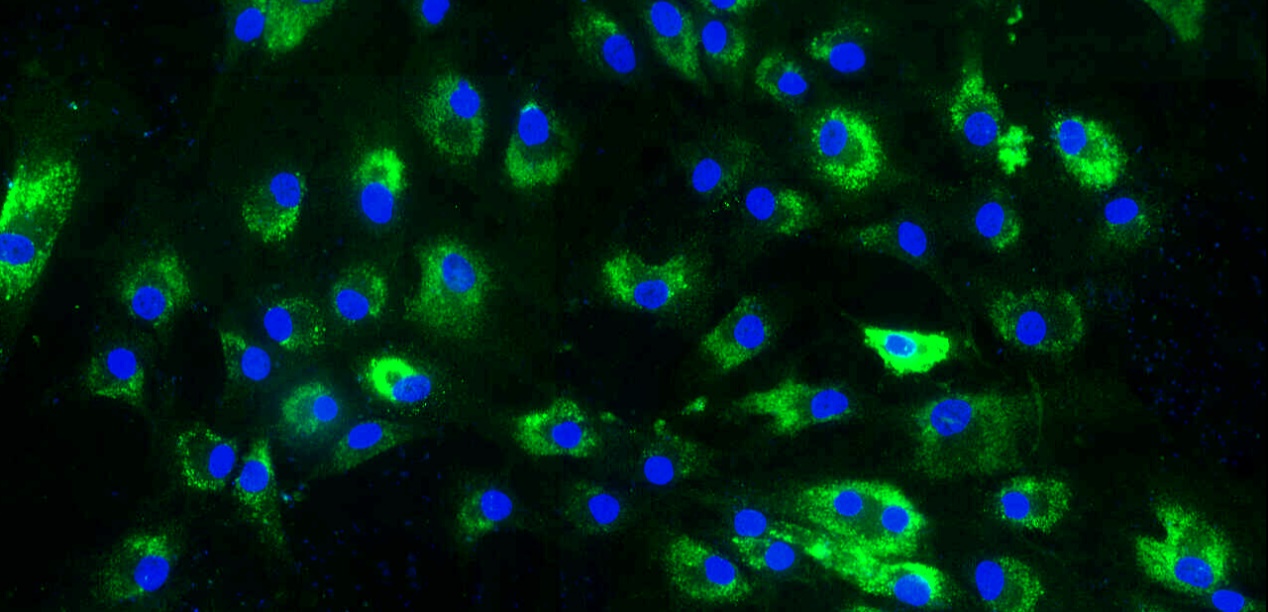


sh circGNB1#2 COL2A1

**Figure2F**

**
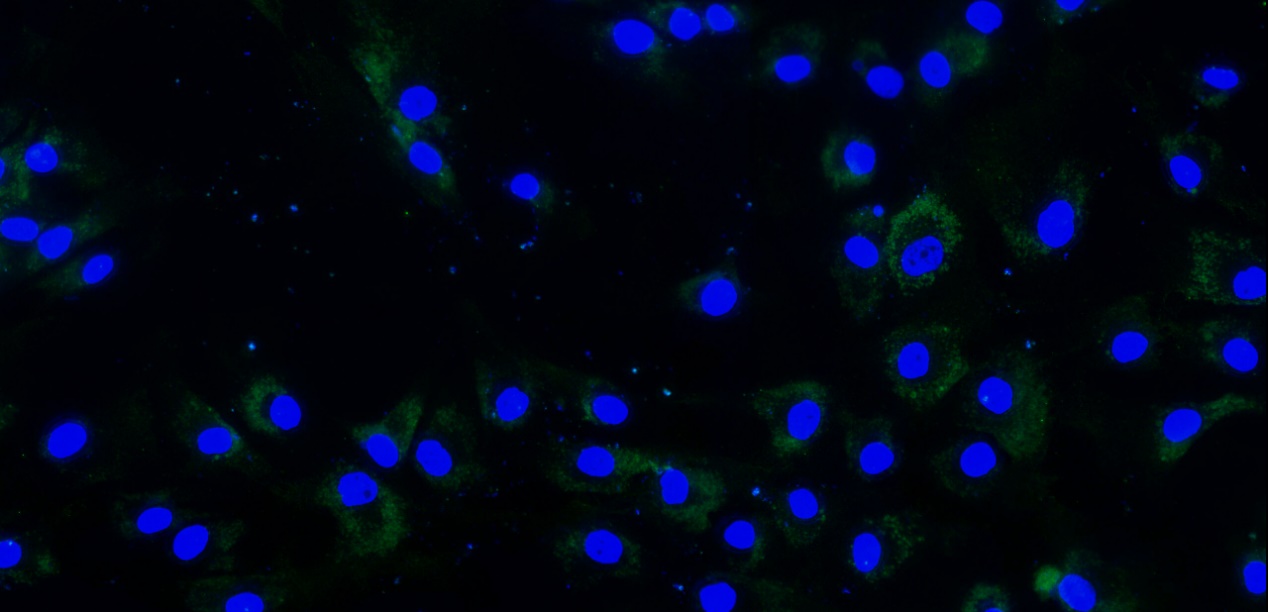
**

NC MMP13


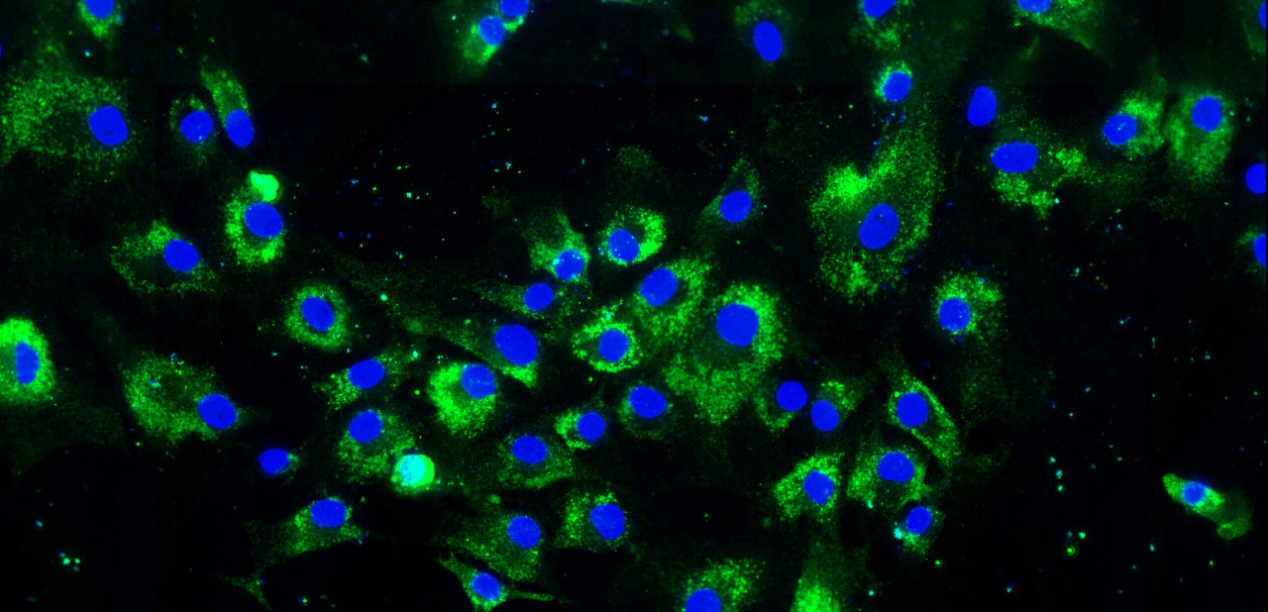


oe circGNB1 MMP13


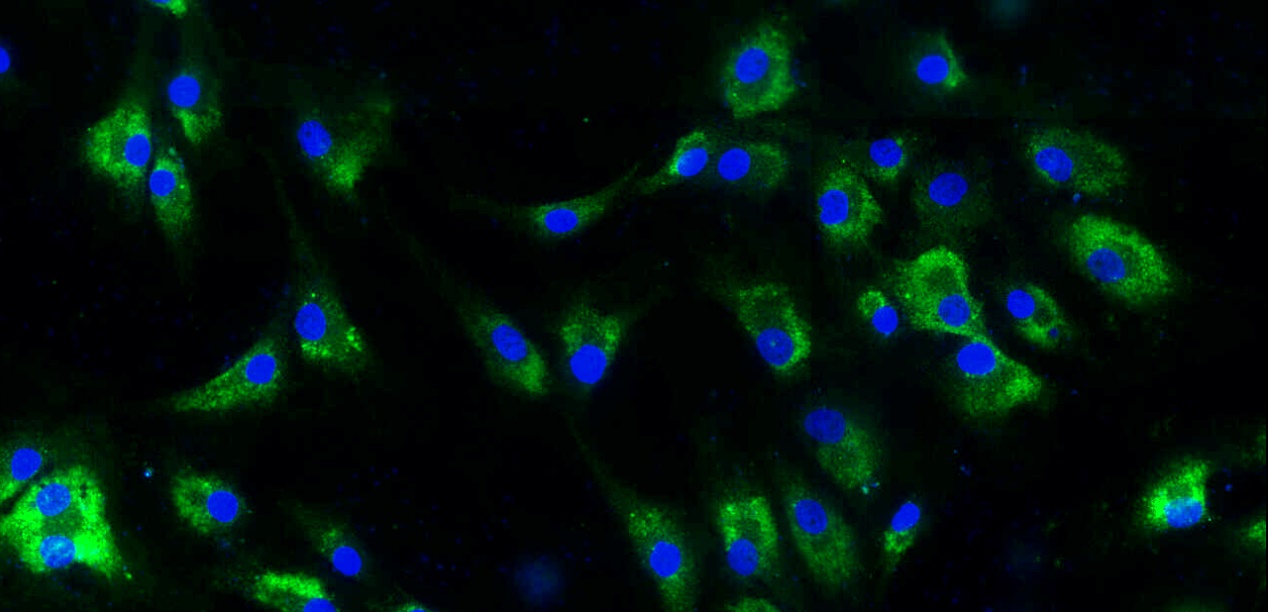


NC COL2A1


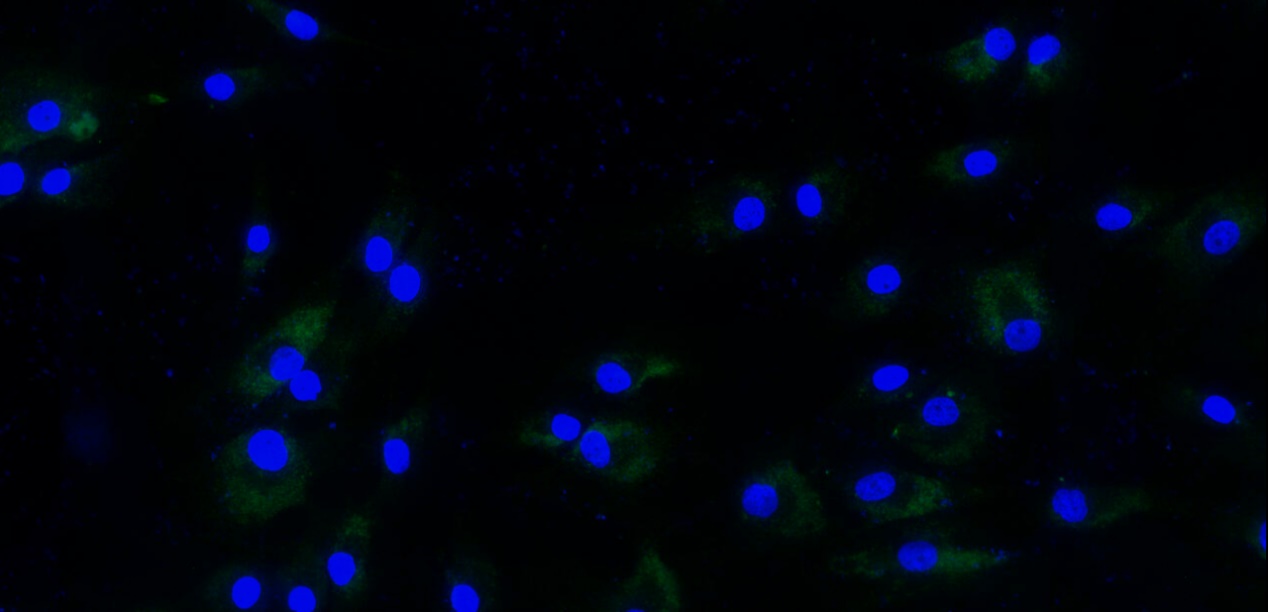


oe circGNB1 COL2A1

**Figure3C**

**
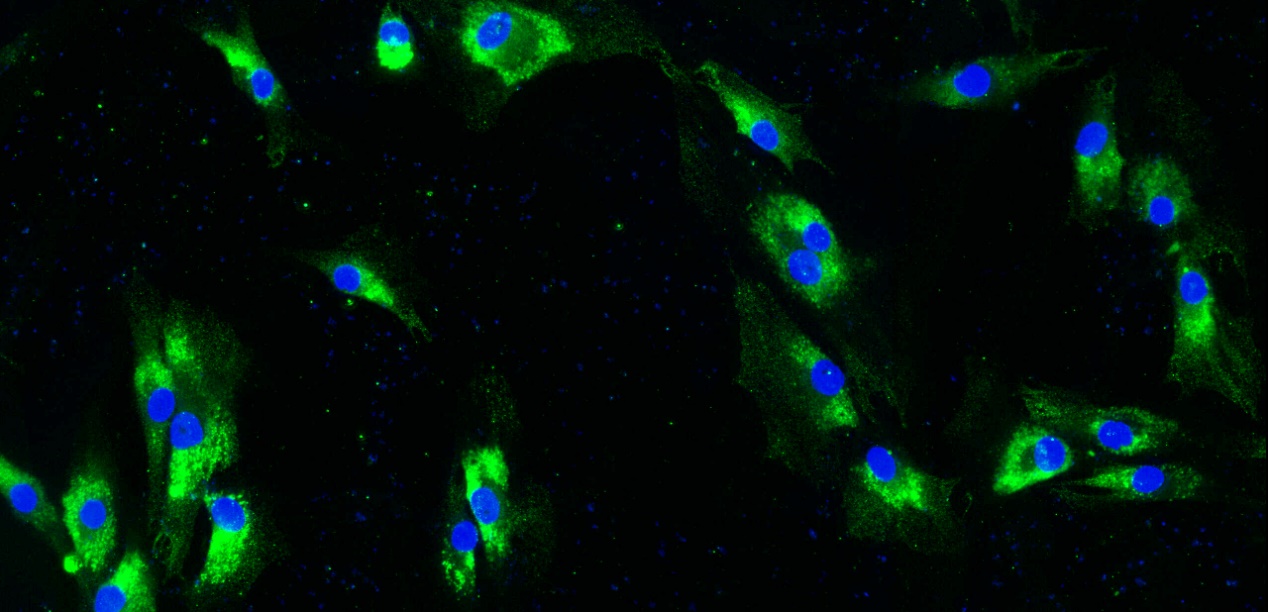
**

NC mimic MMP13


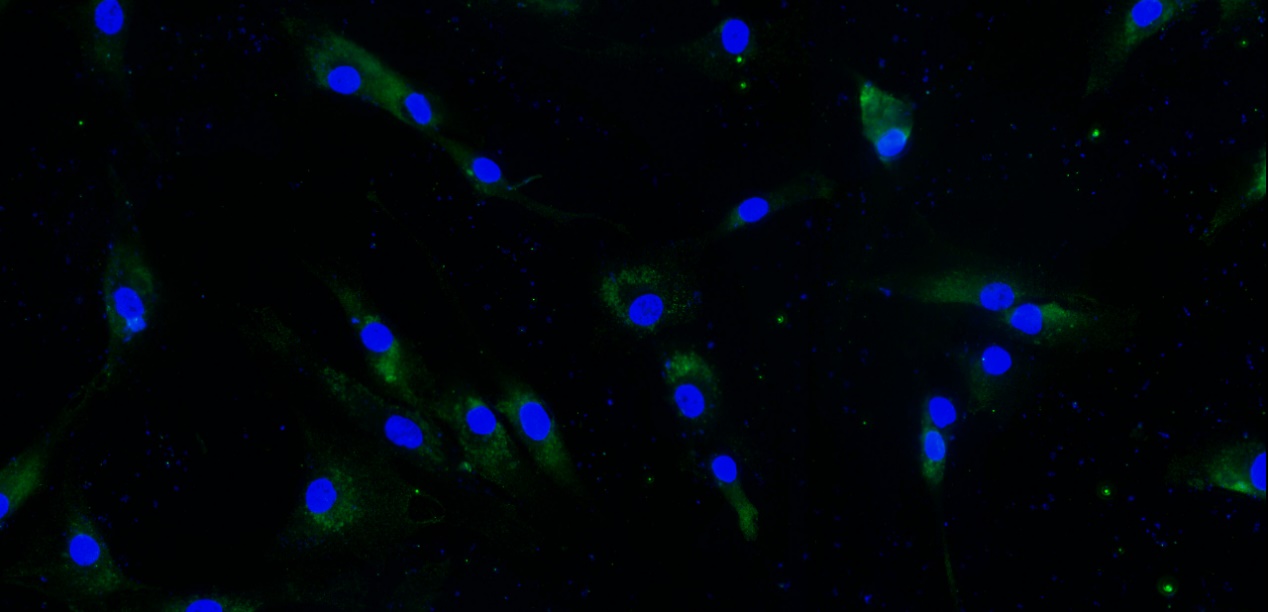


miR-152-3p MMP13


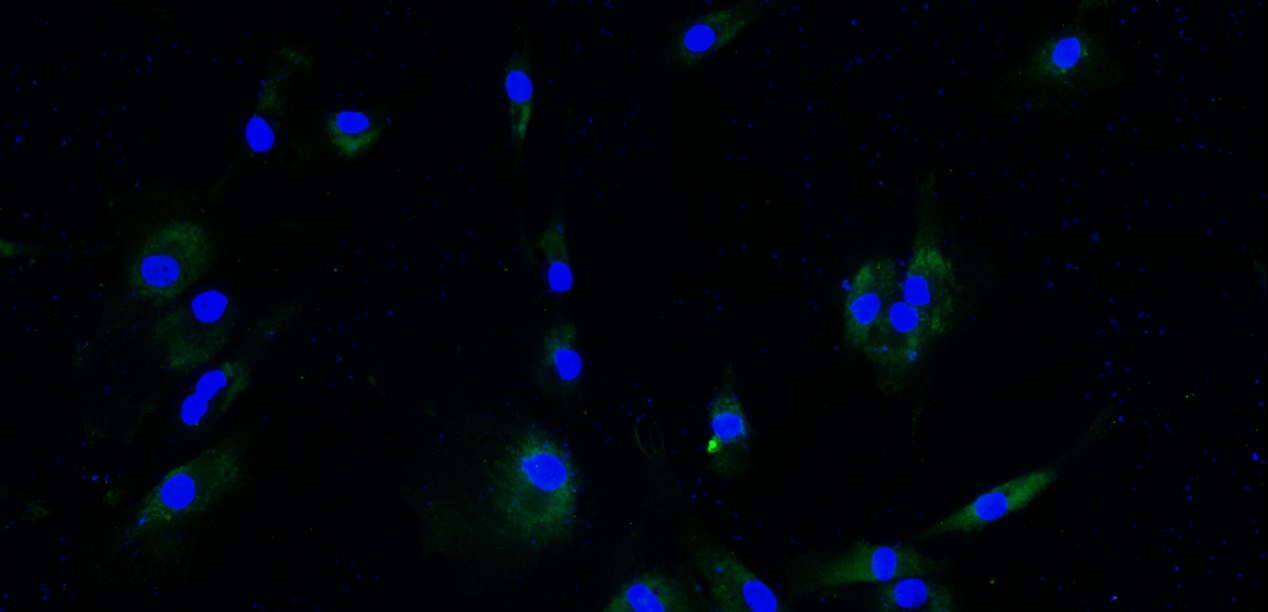


NC inhibitor MMP13


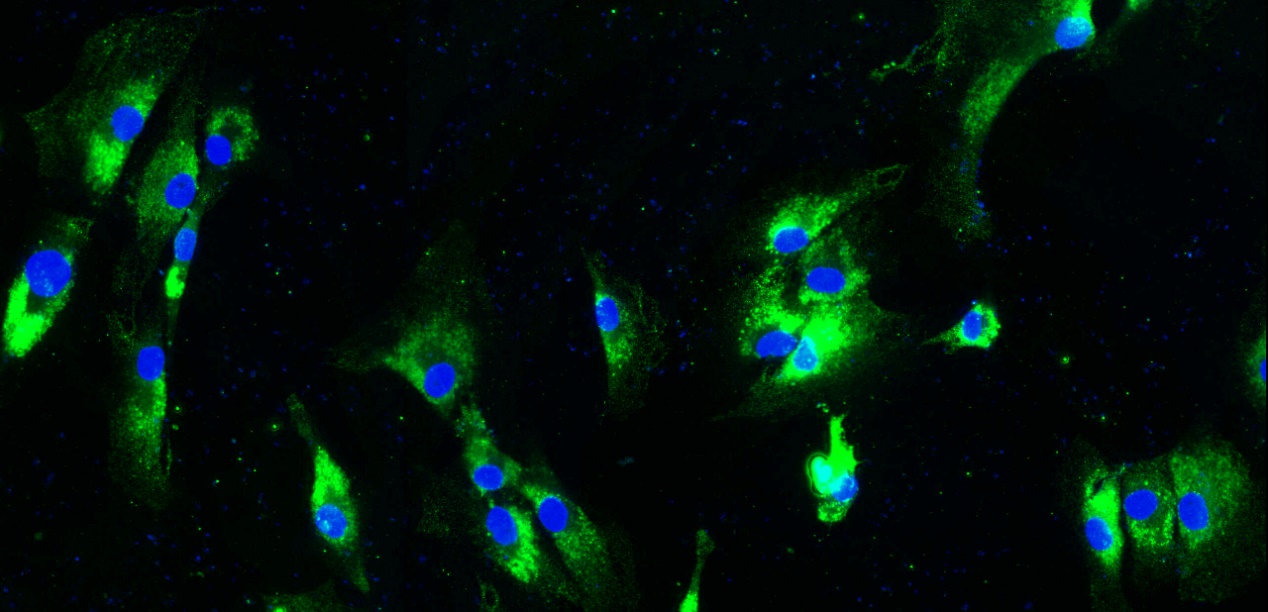


miR-152-3p inhibitor MMP13


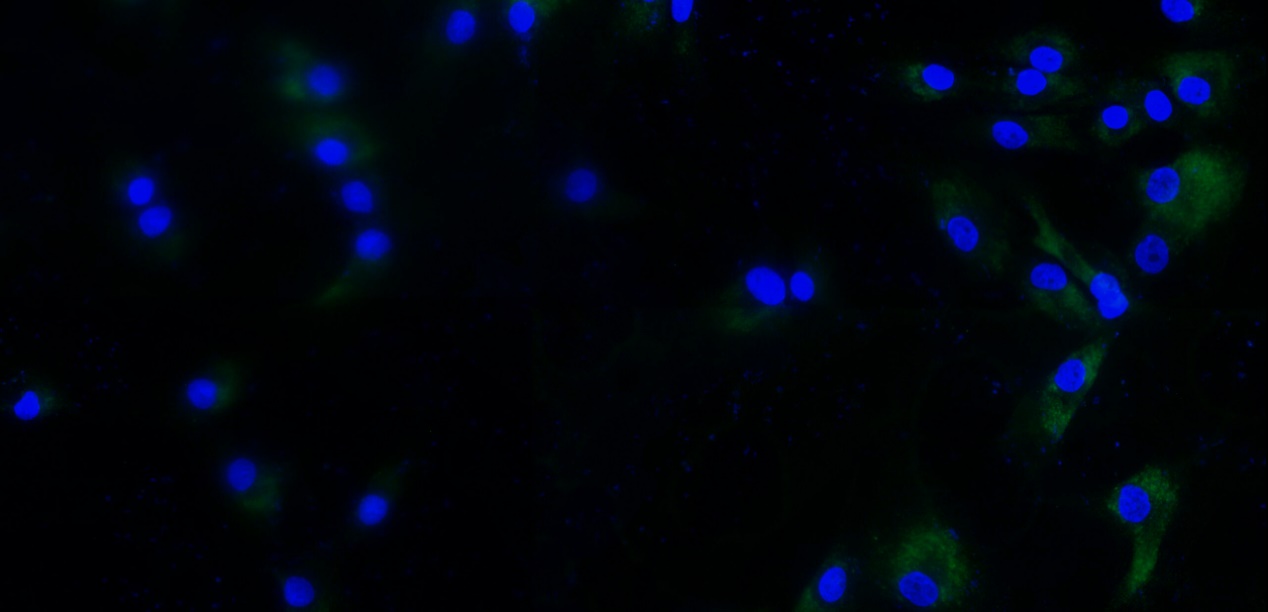


NC mimic COL2A1


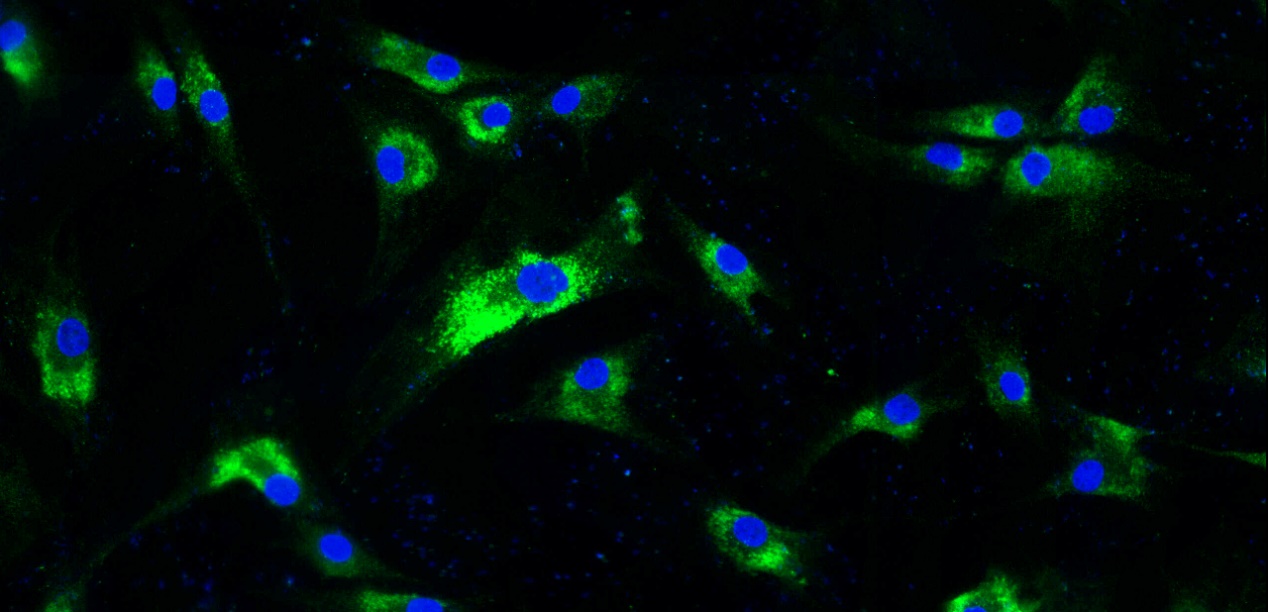


miR-152-3p COL2A1


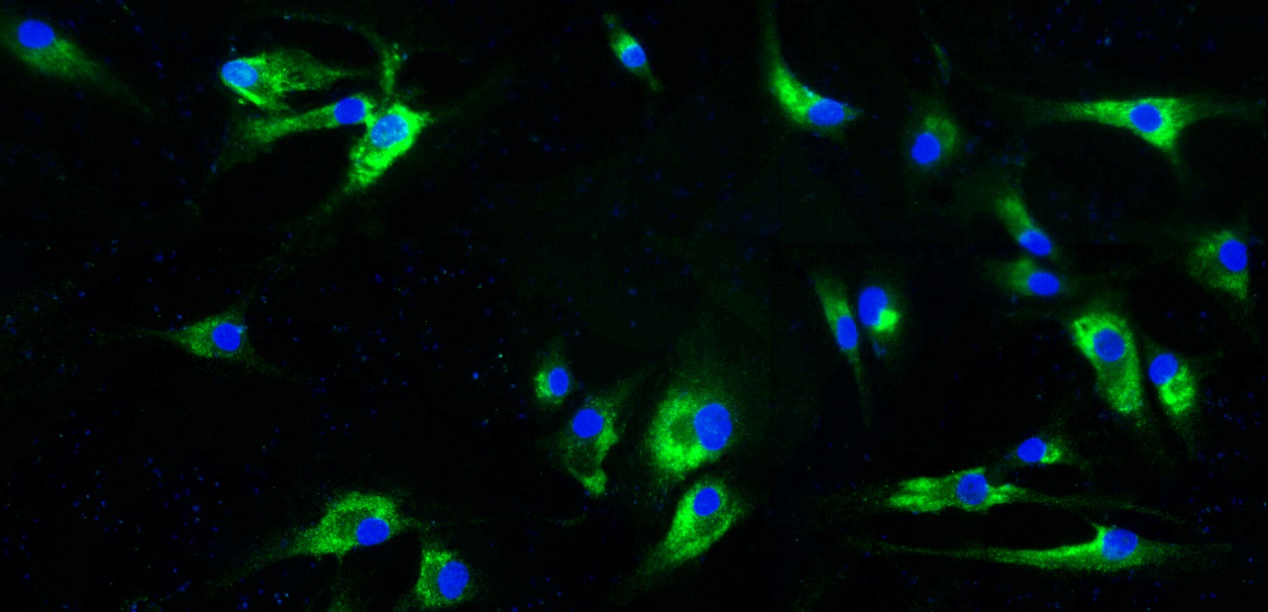


NC inhibitor COL2A1


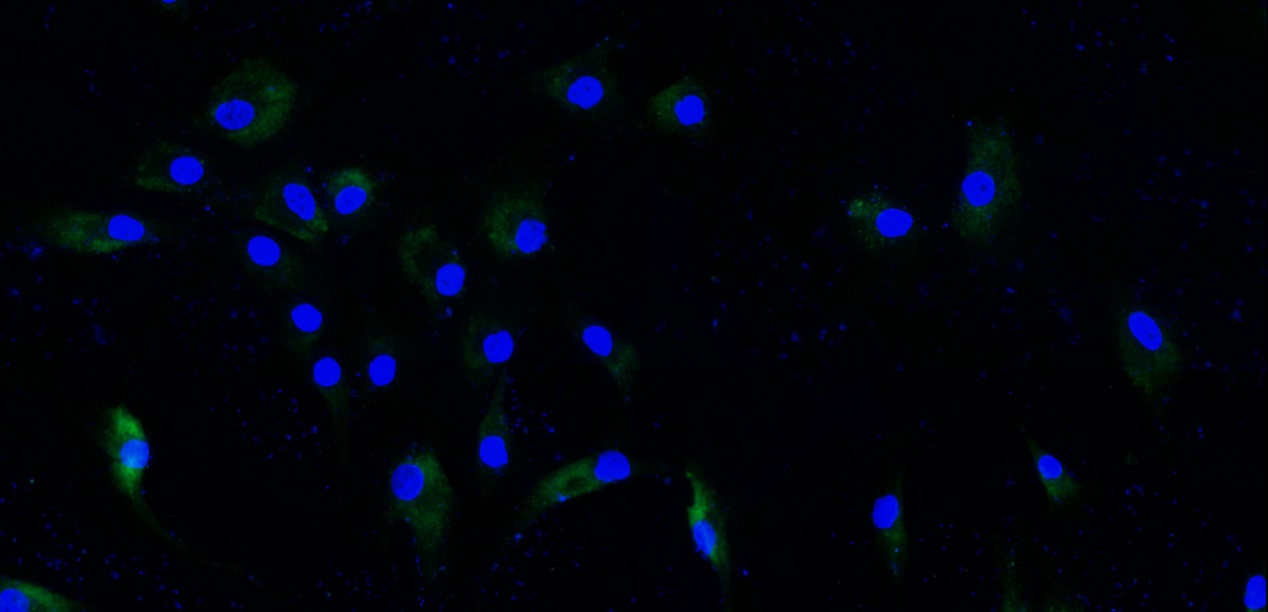


miR-152-3p inhibitor COL2A1

**Figure4C**

**
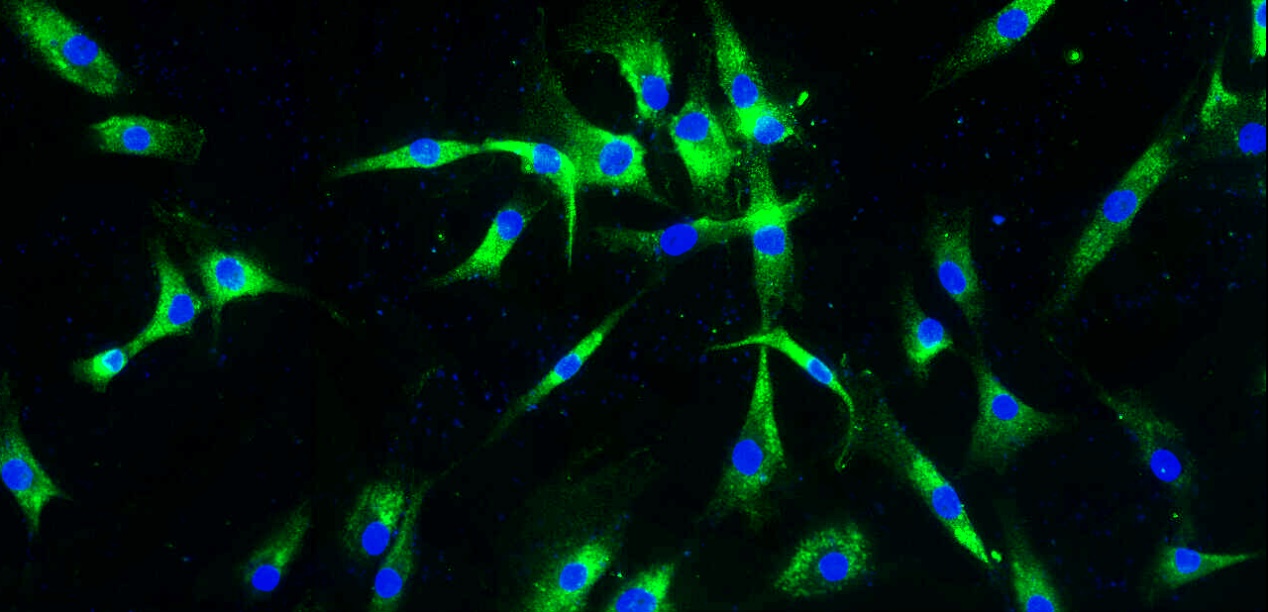
**

NC MMP13


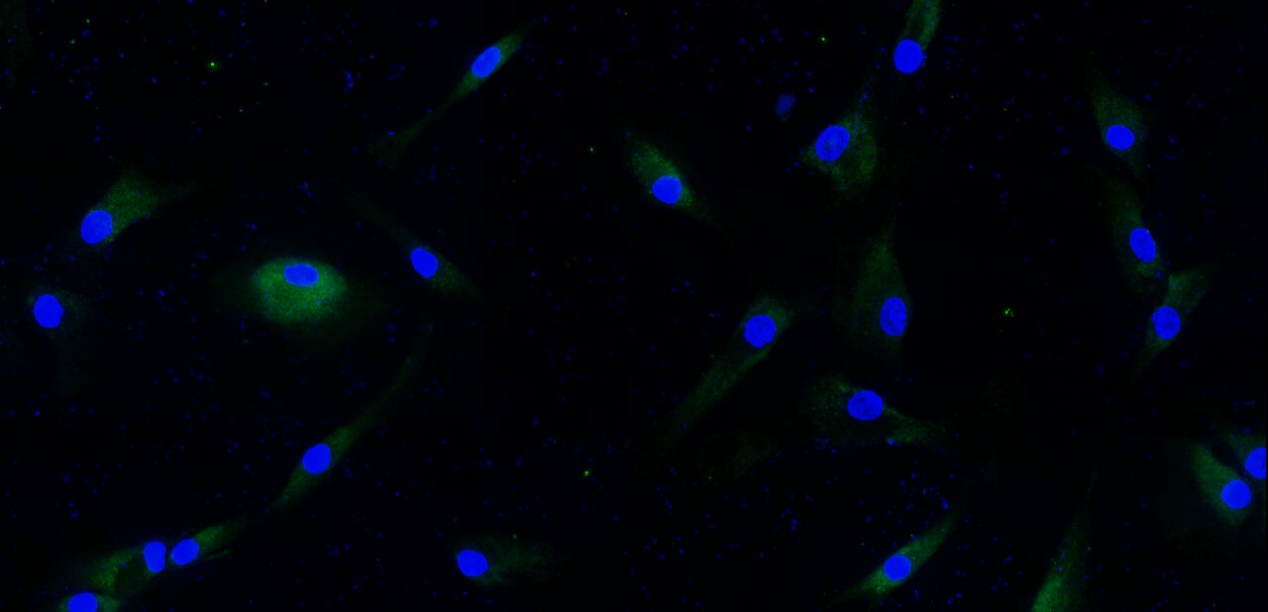


sh RNF219#1 MMP13


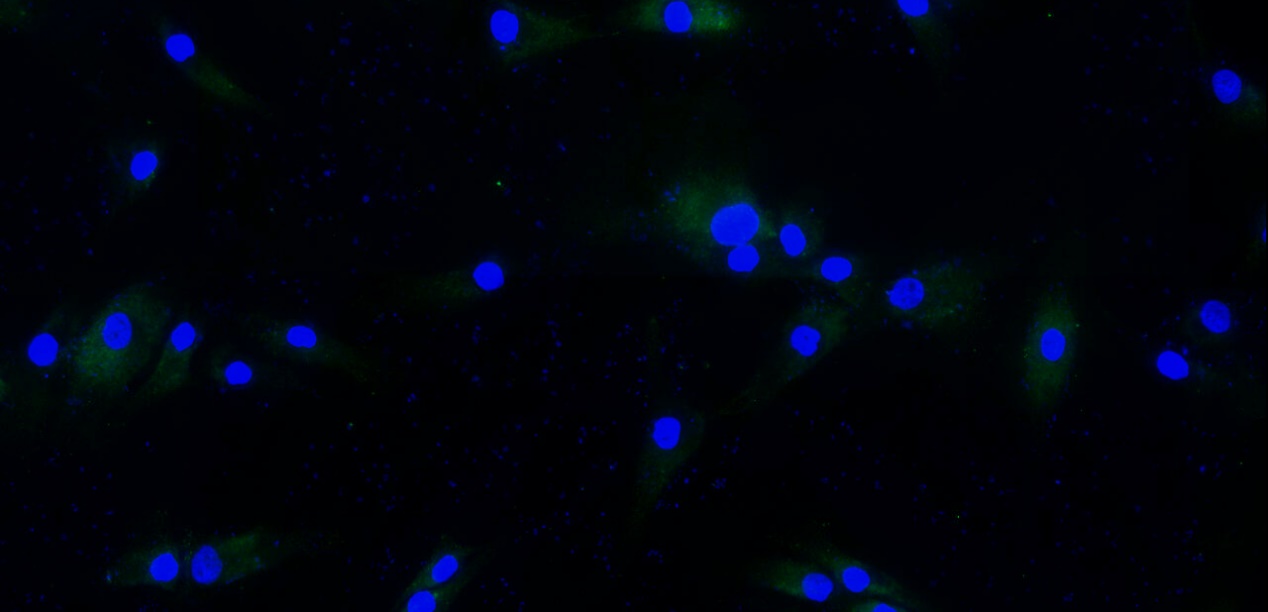


sh RNF219#2 MMP13


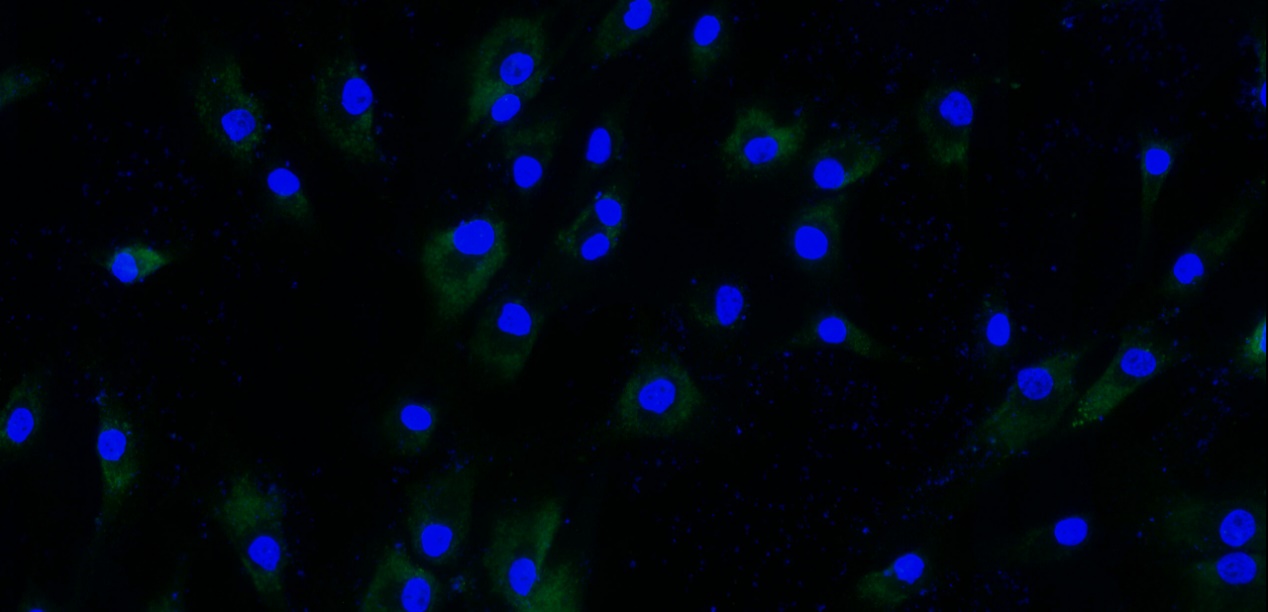


NC COL2A1


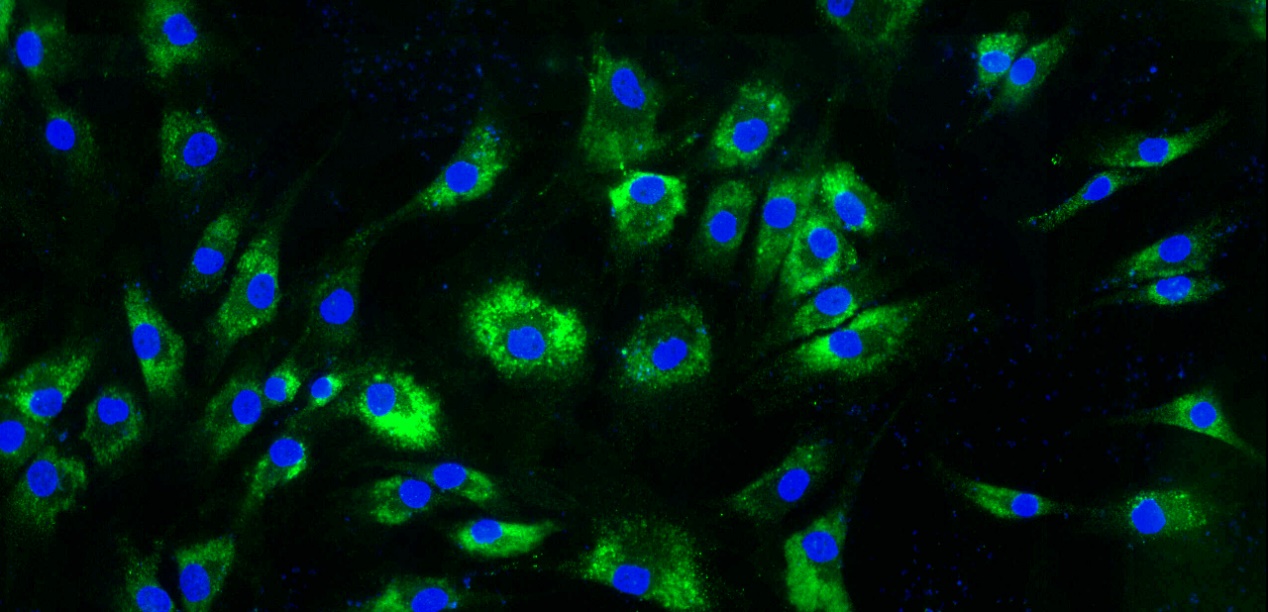


sh RNF219#1 COL2A1


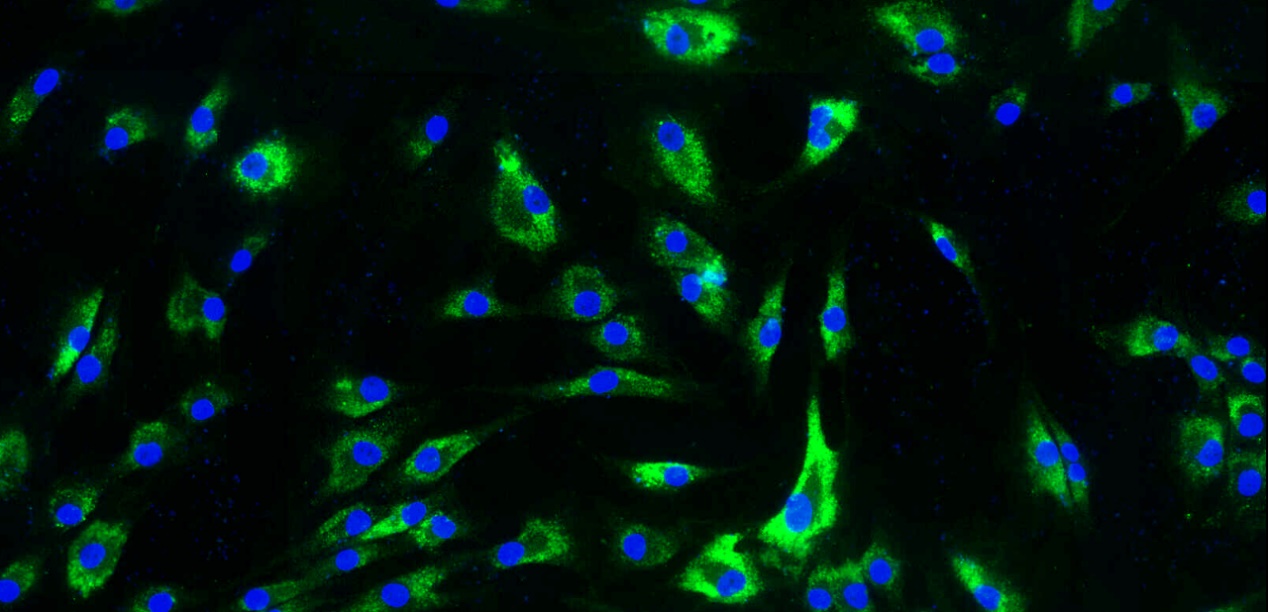


sh RNF219#2 COL2A1

**Figure4F**

**
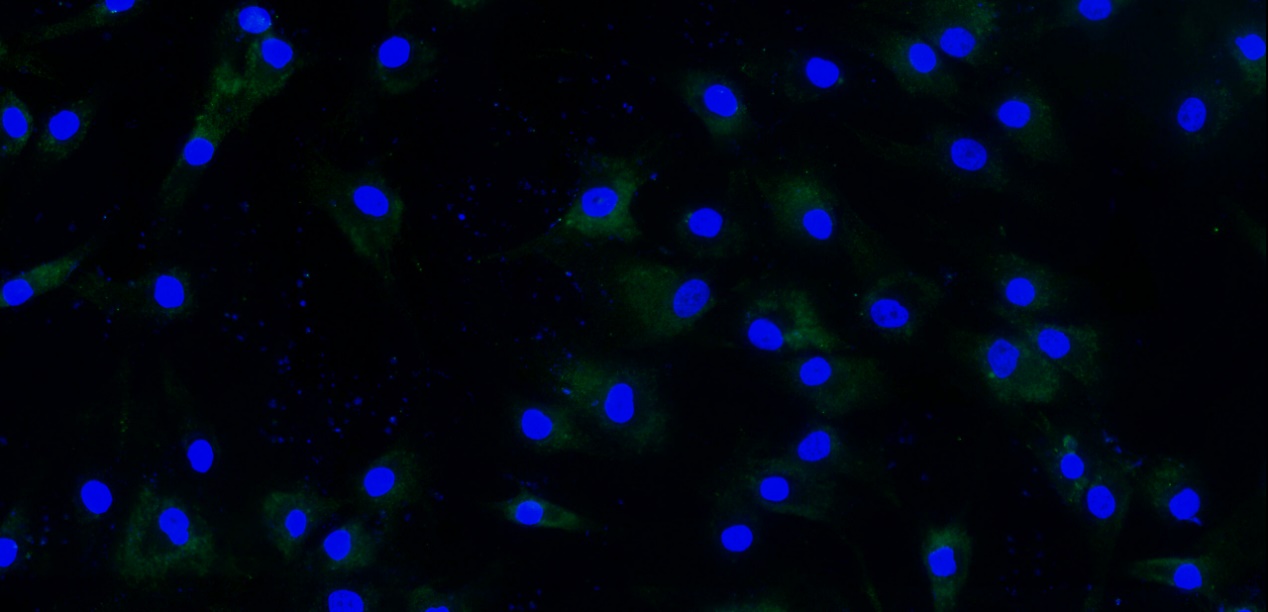
**

NC MMP13


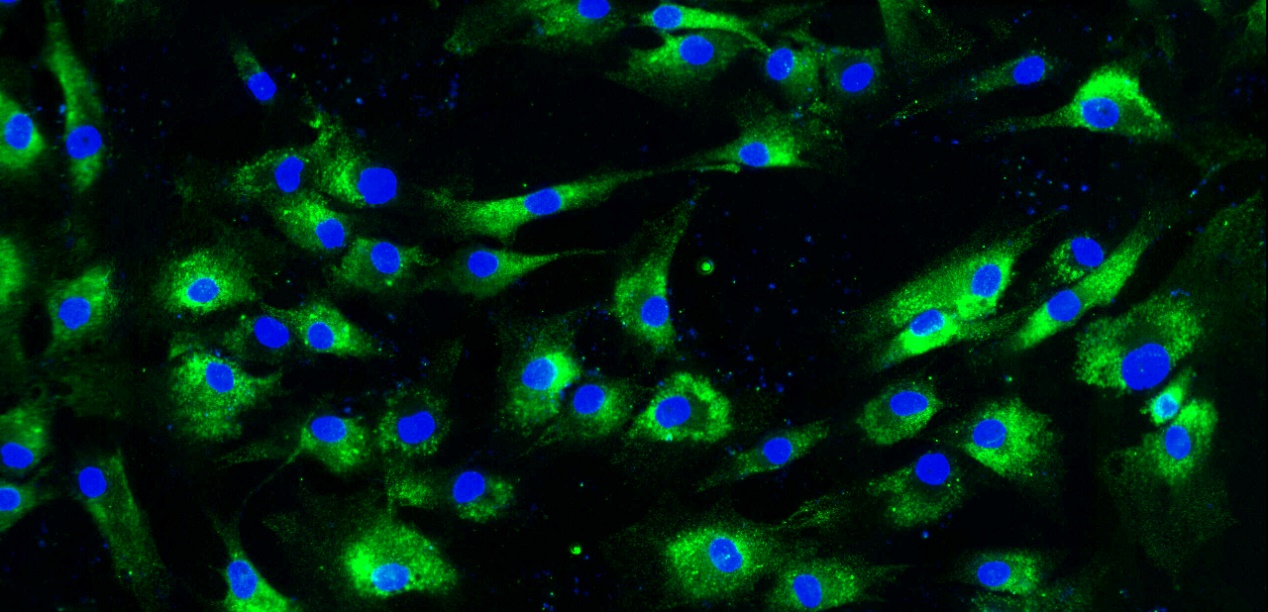


oe RNF219 MMP13


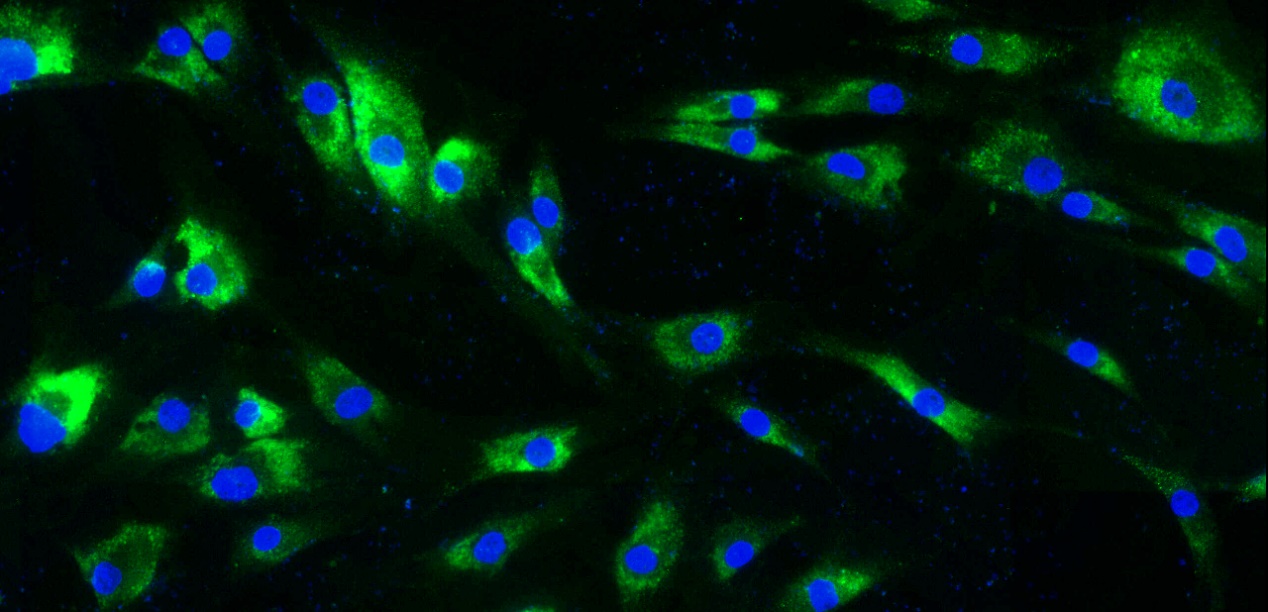


NC COL2A1


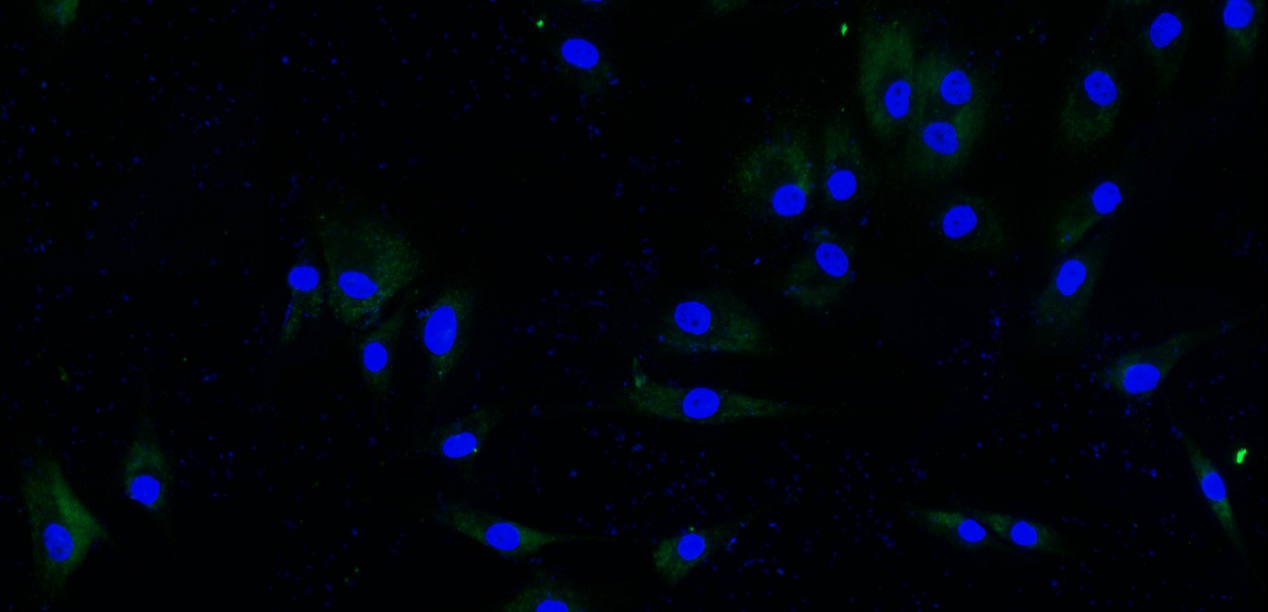


oe RNF219 COL2A1

**Figure5D**

**
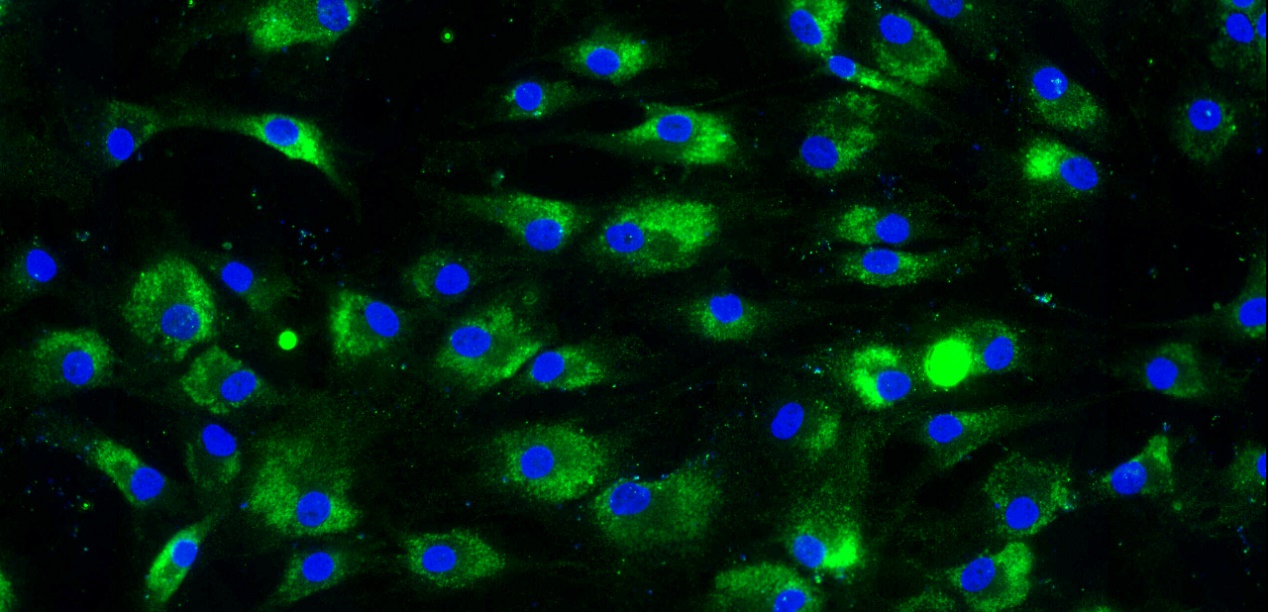
**

NC MMP13


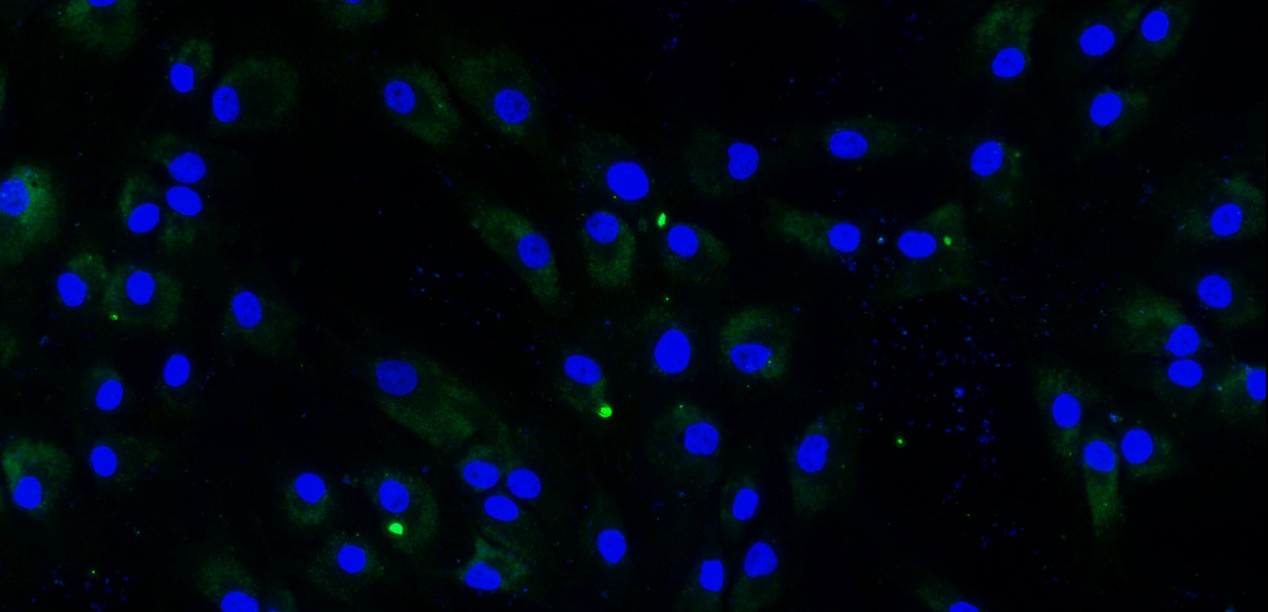


sh CAV1#1 MMP13


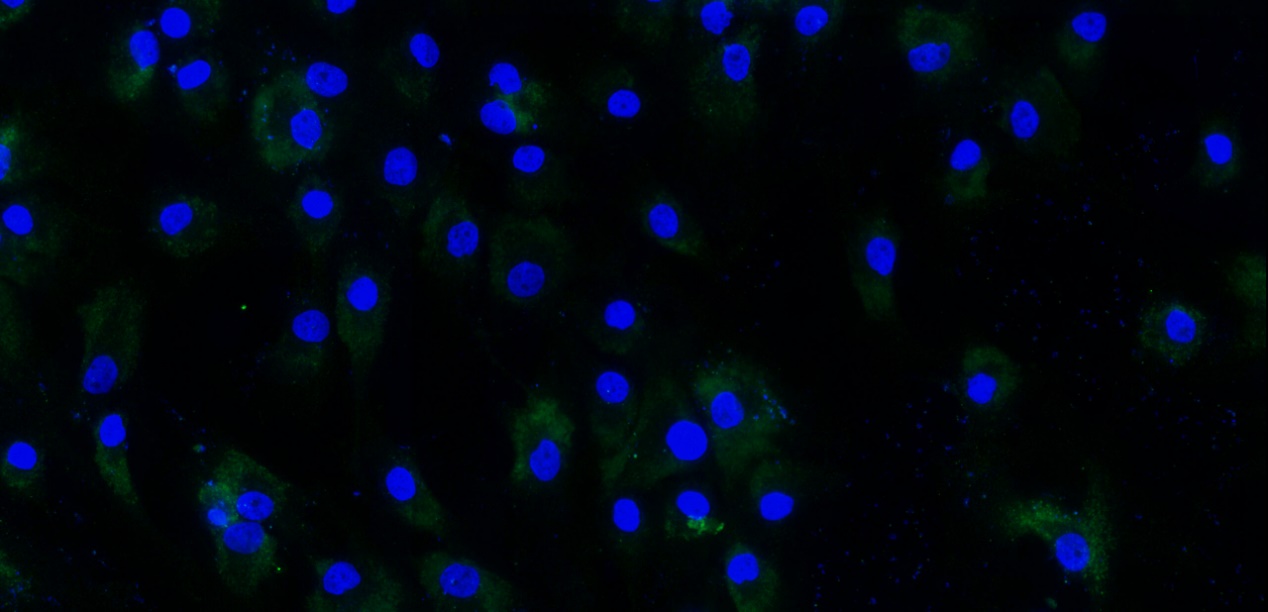


sh CAV1#2 MMP13


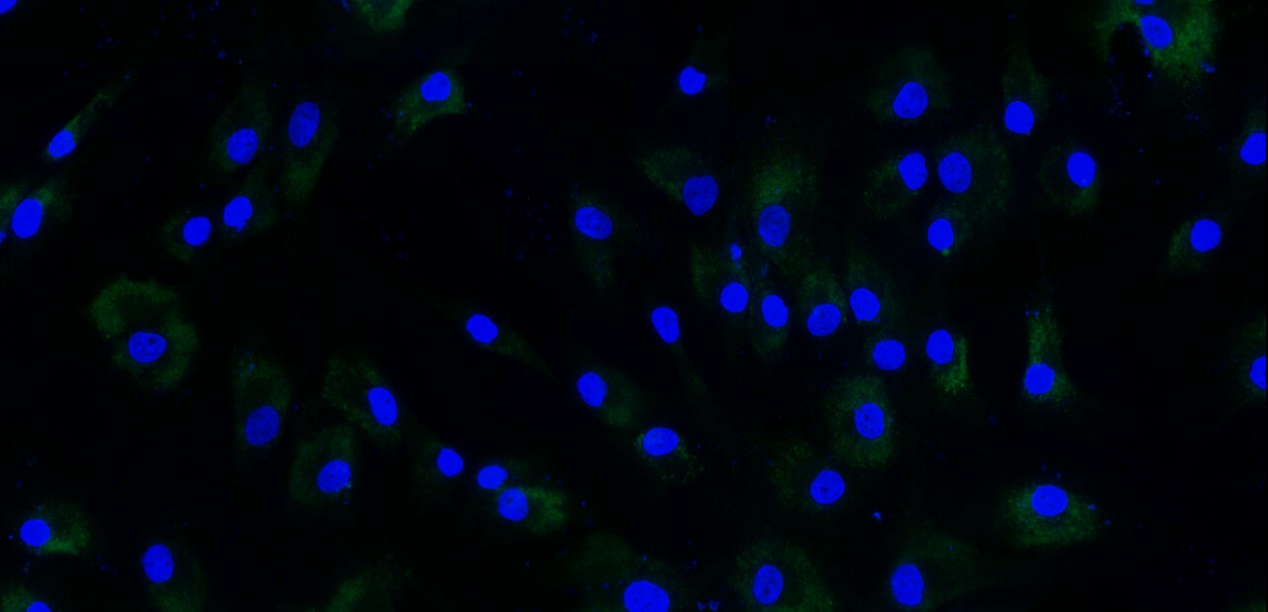


NC COL2A1


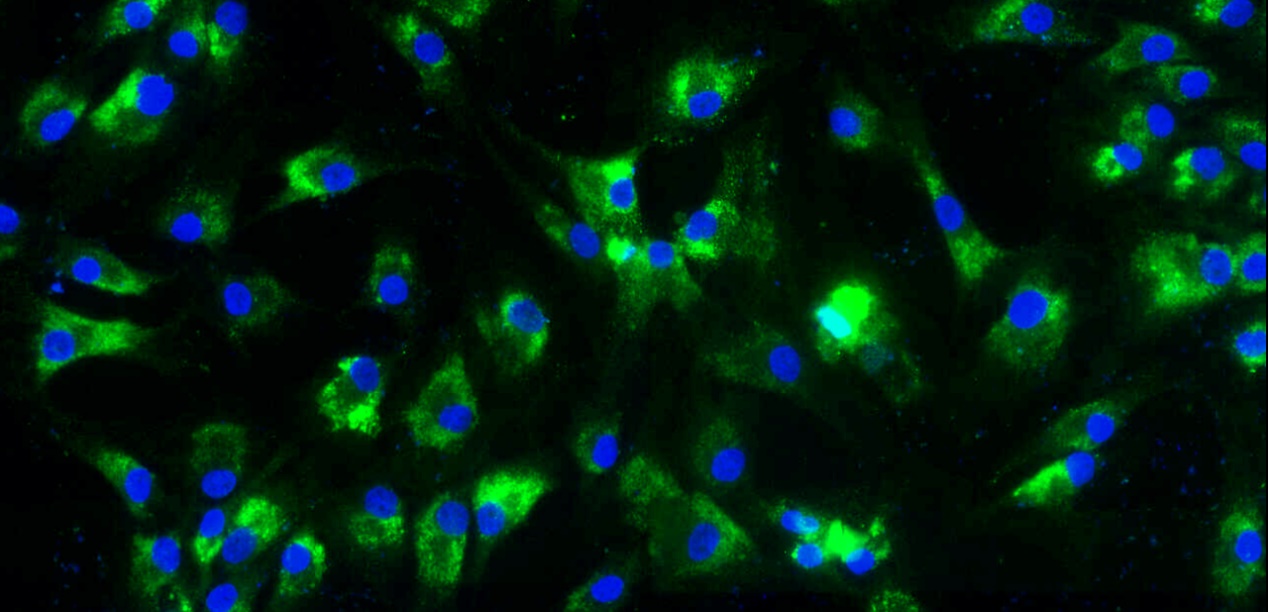


sh CAV1#1 COL2A1


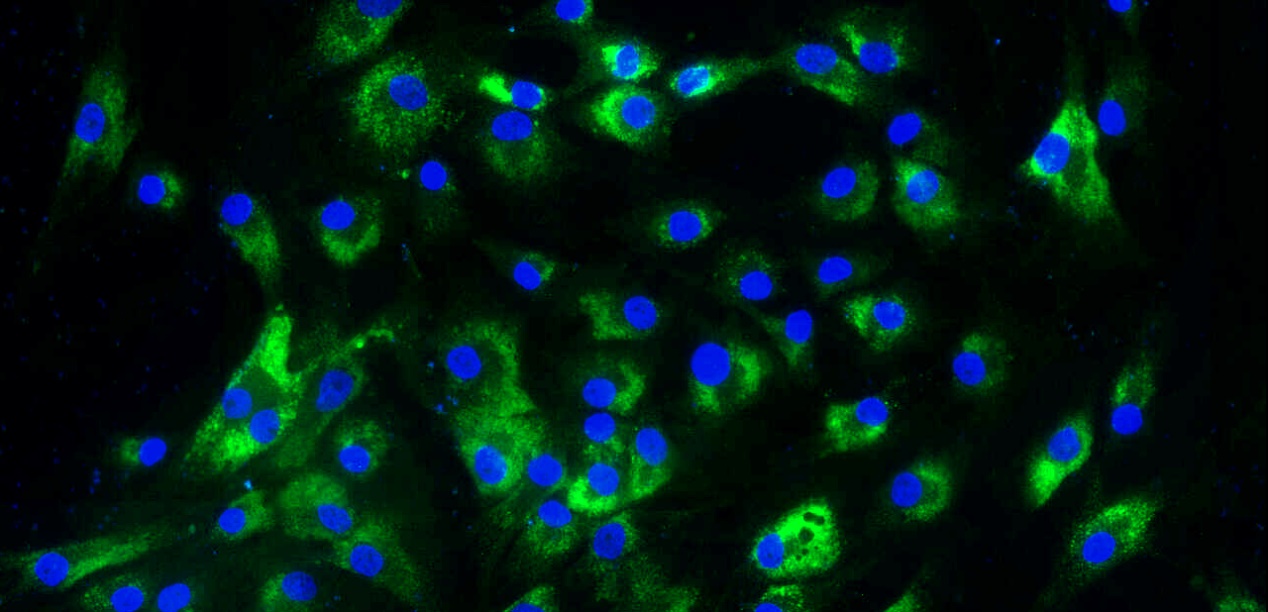


sh CAV1#2 COL2A1

**Figure5G**

**
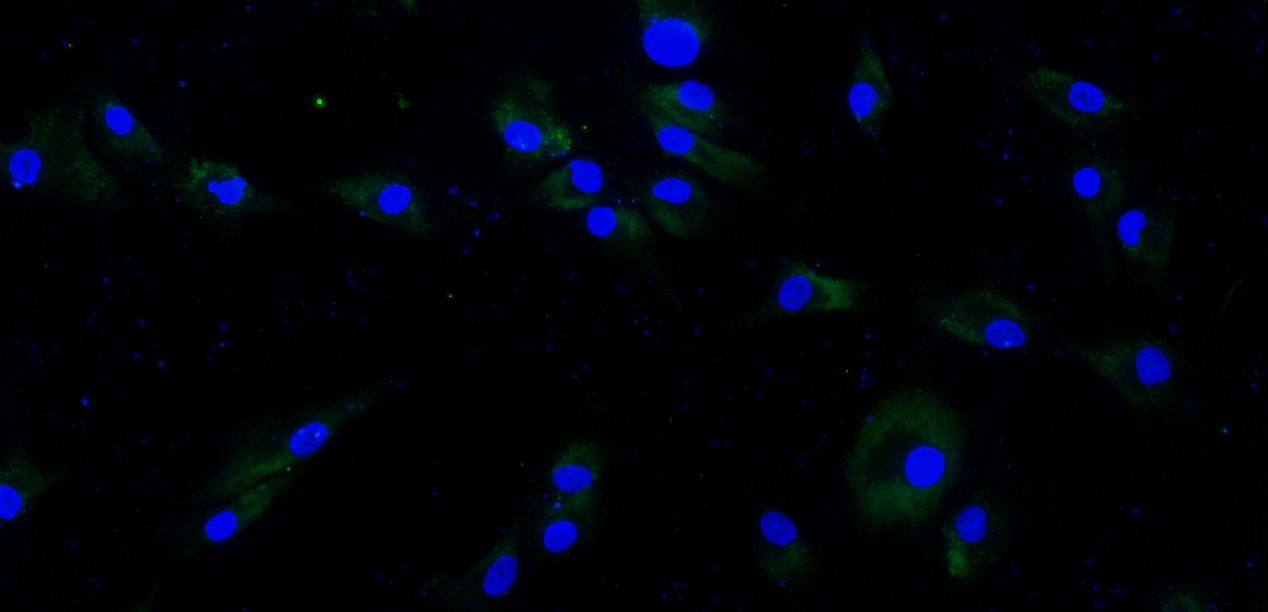
**

NC MMP13


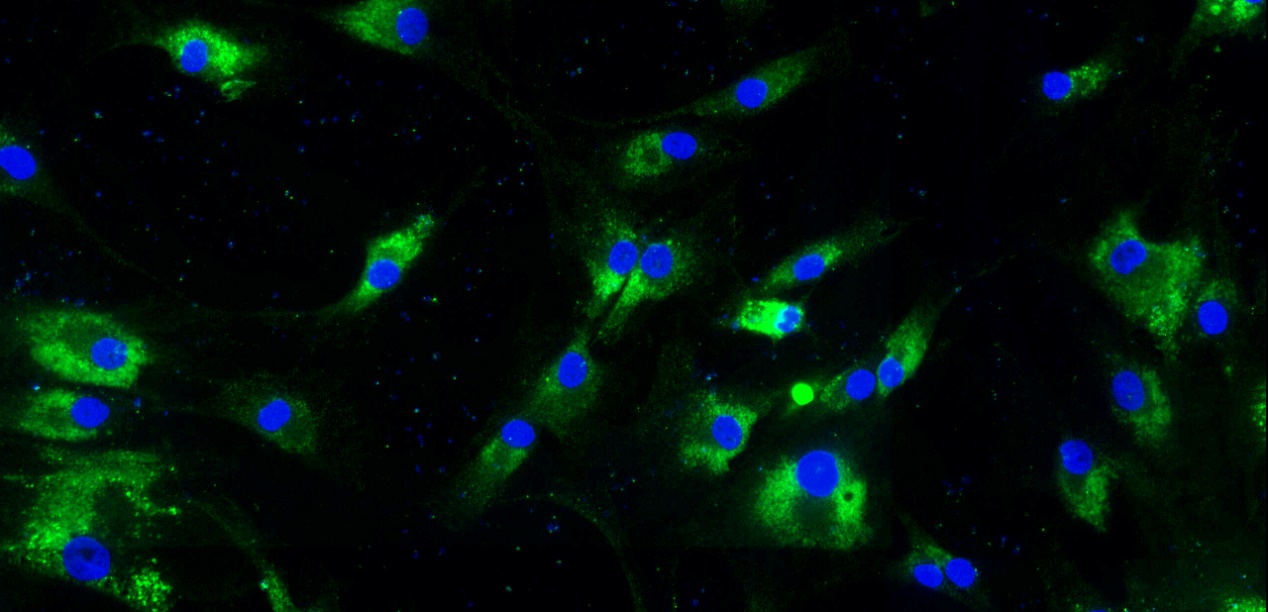


oe CAV1 MMP13


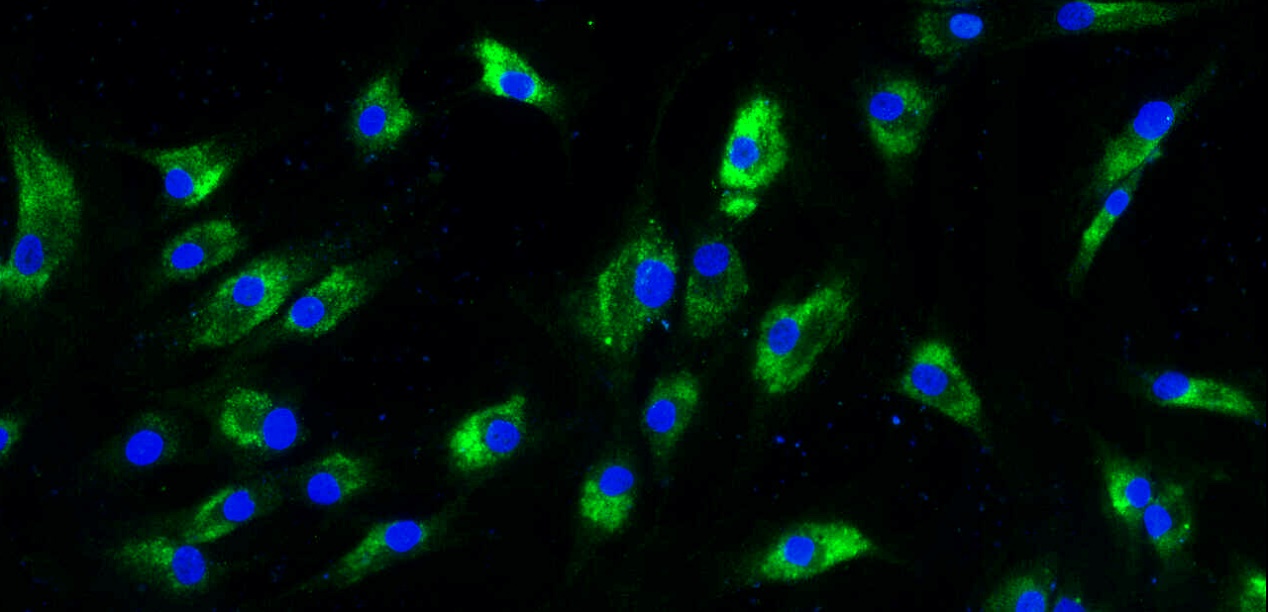


NC COL2A1


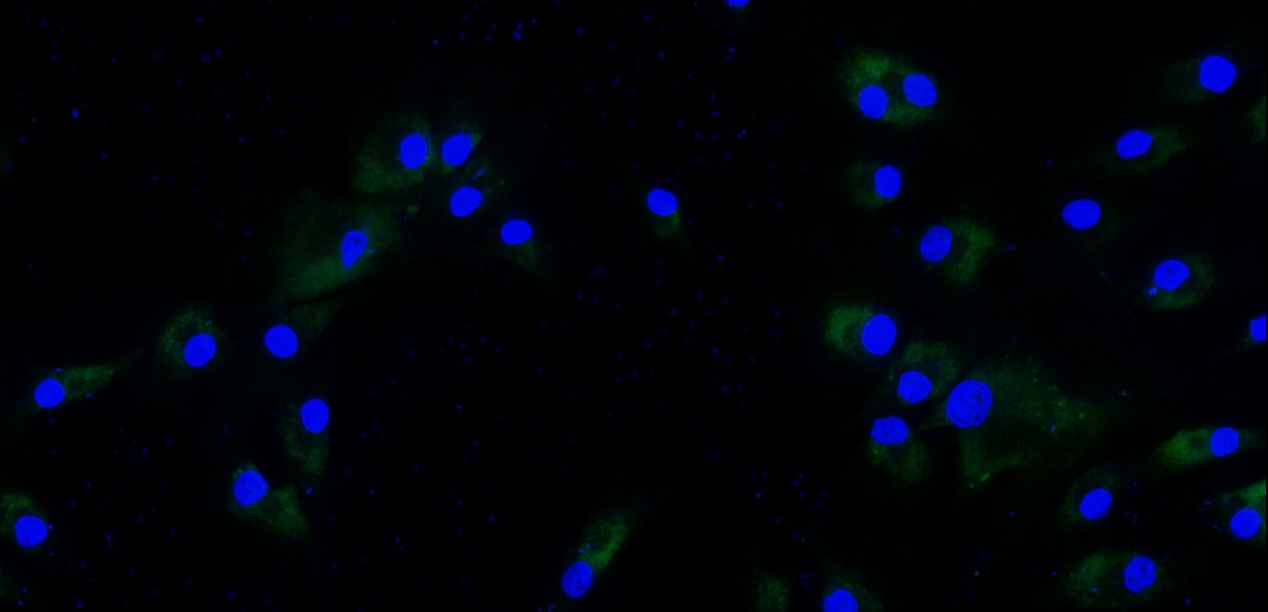


oe CAV1 COL2A1

**Figure6C**

**
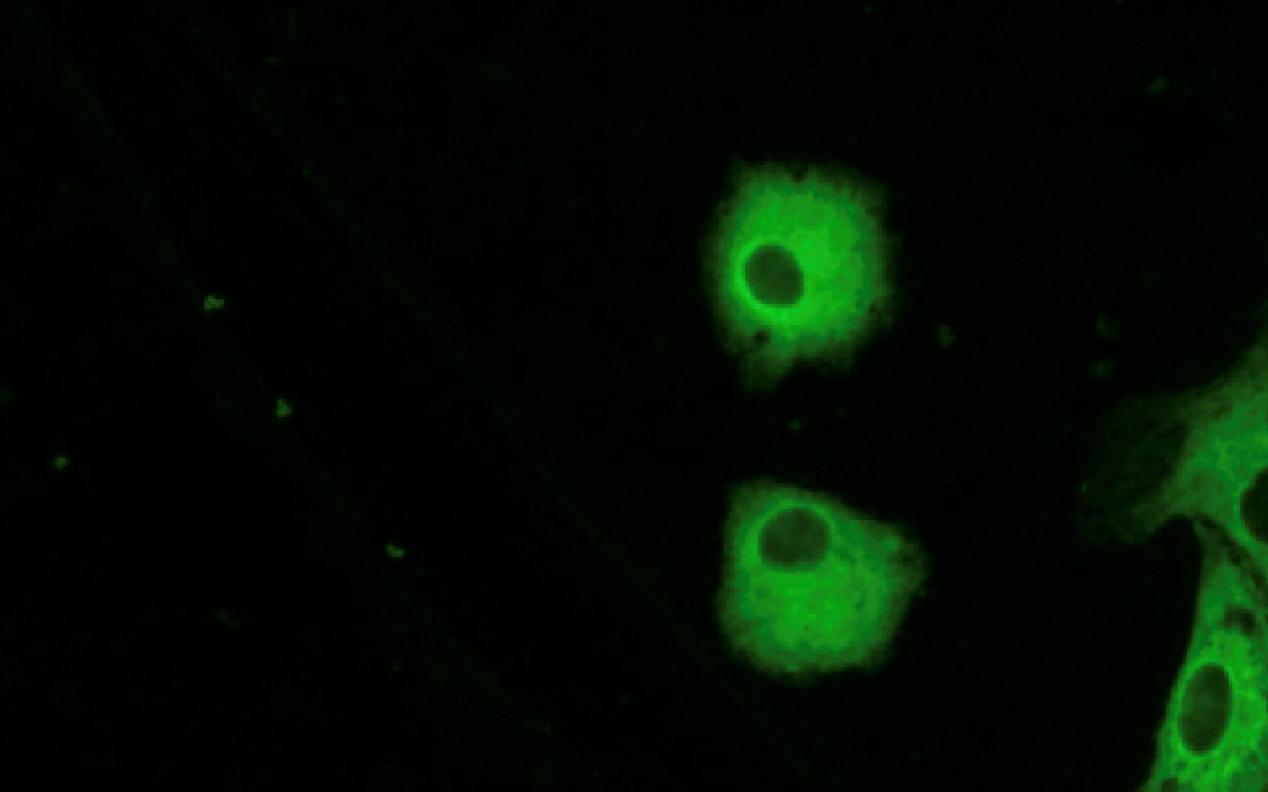
**

CAV1


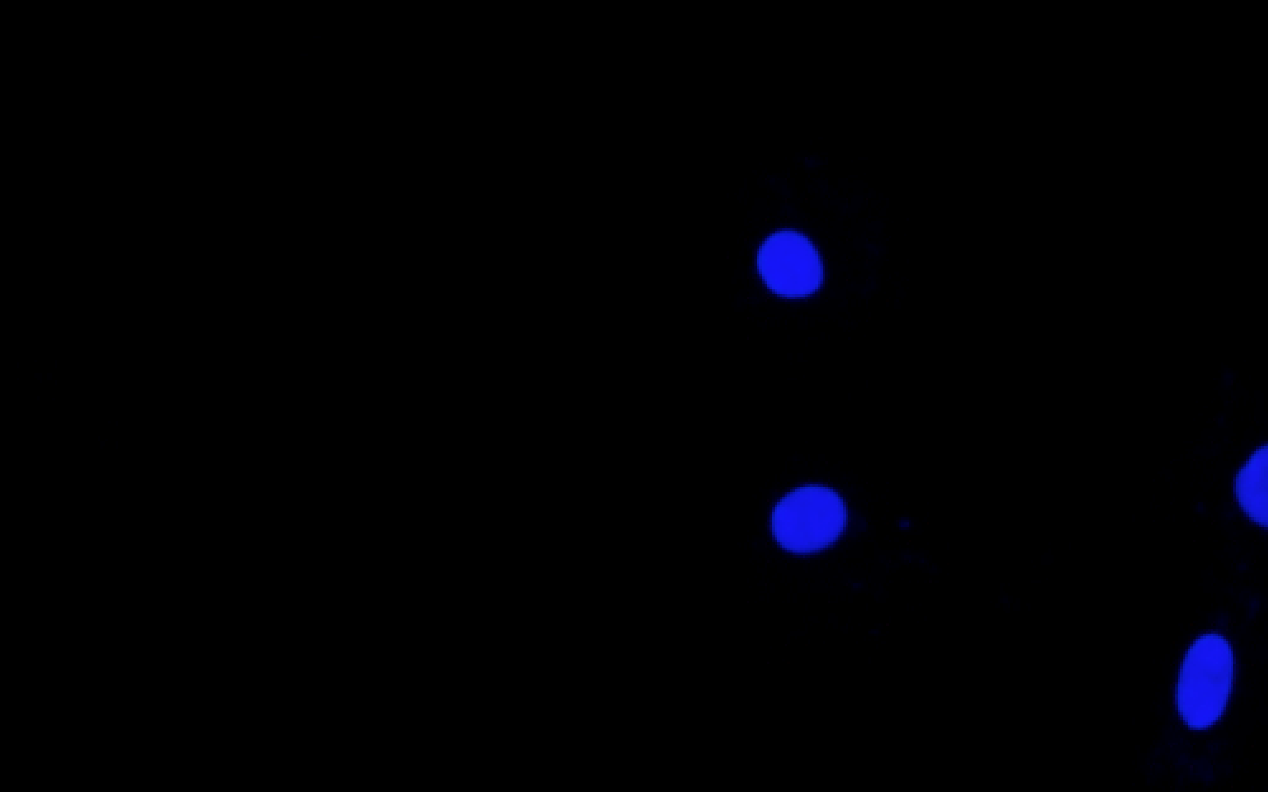


DAPI
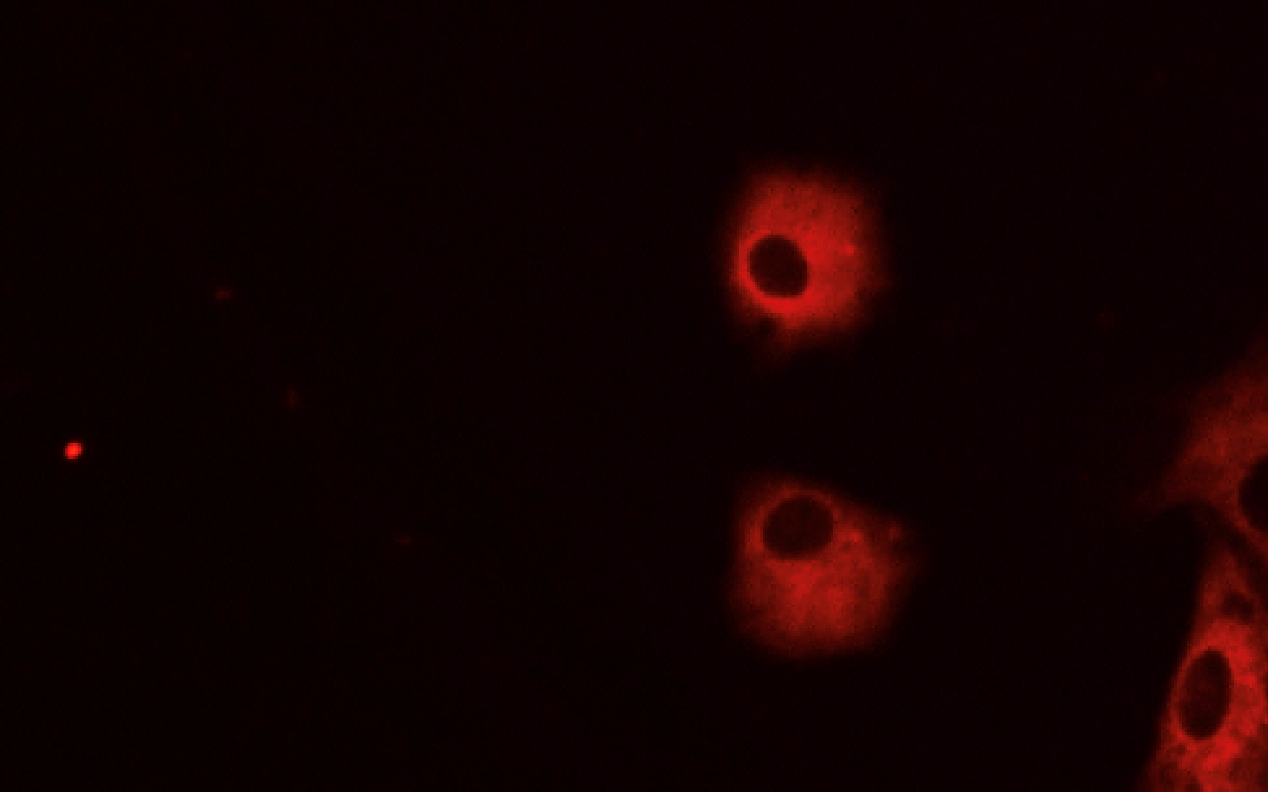


RNF219
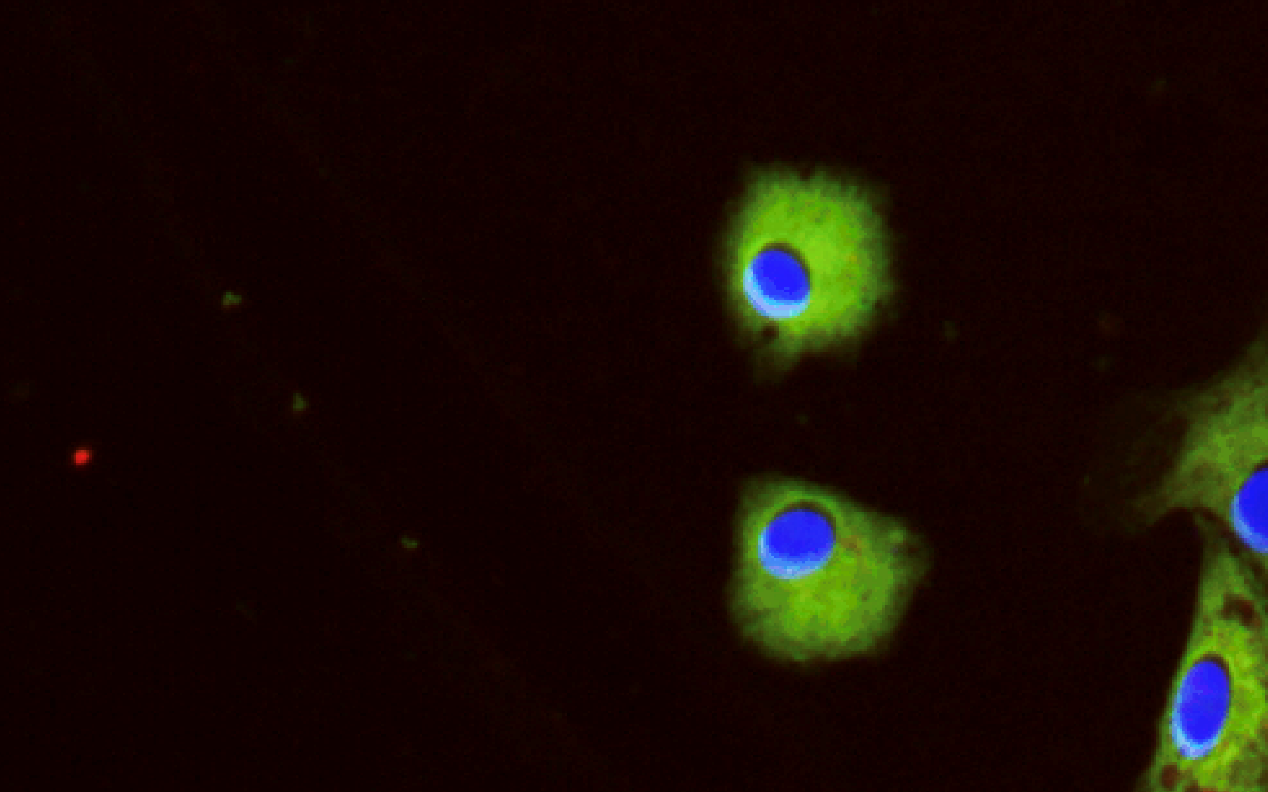


Merge

**Figure9A**

**
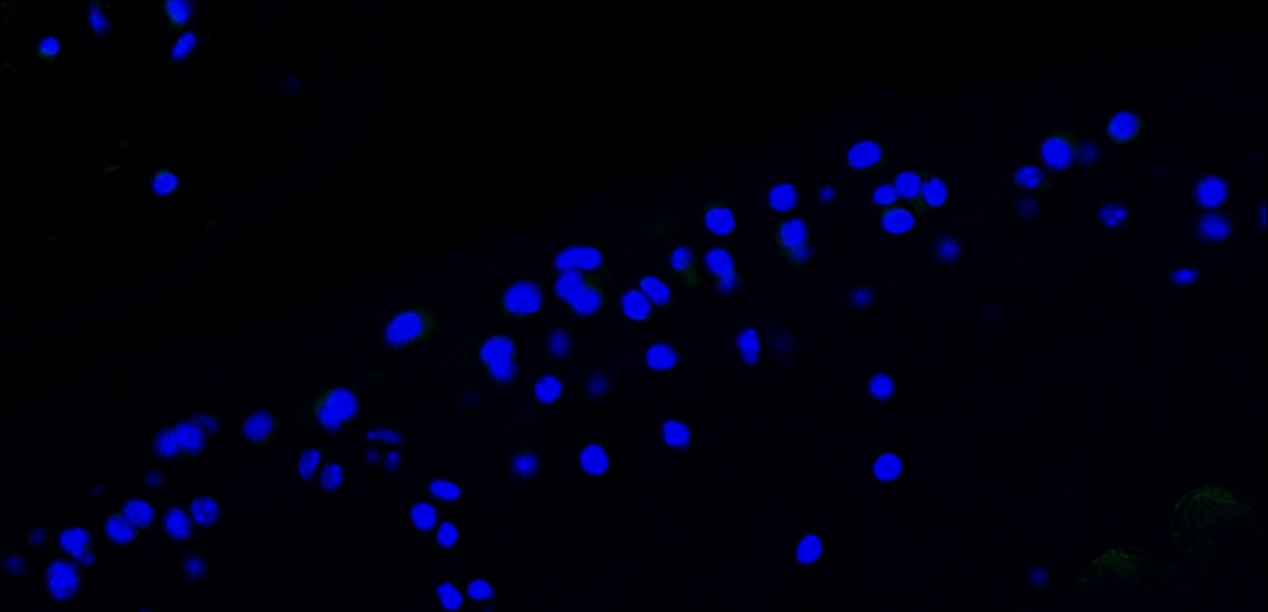
**

sham circGNB1


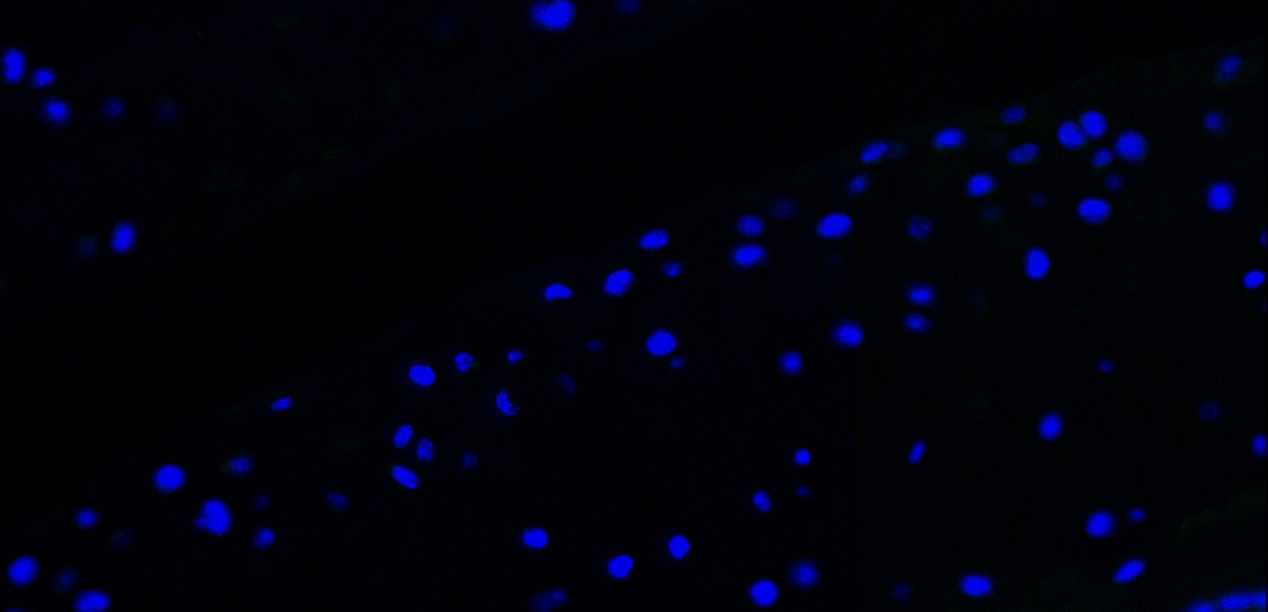


sham+NC circGNB1


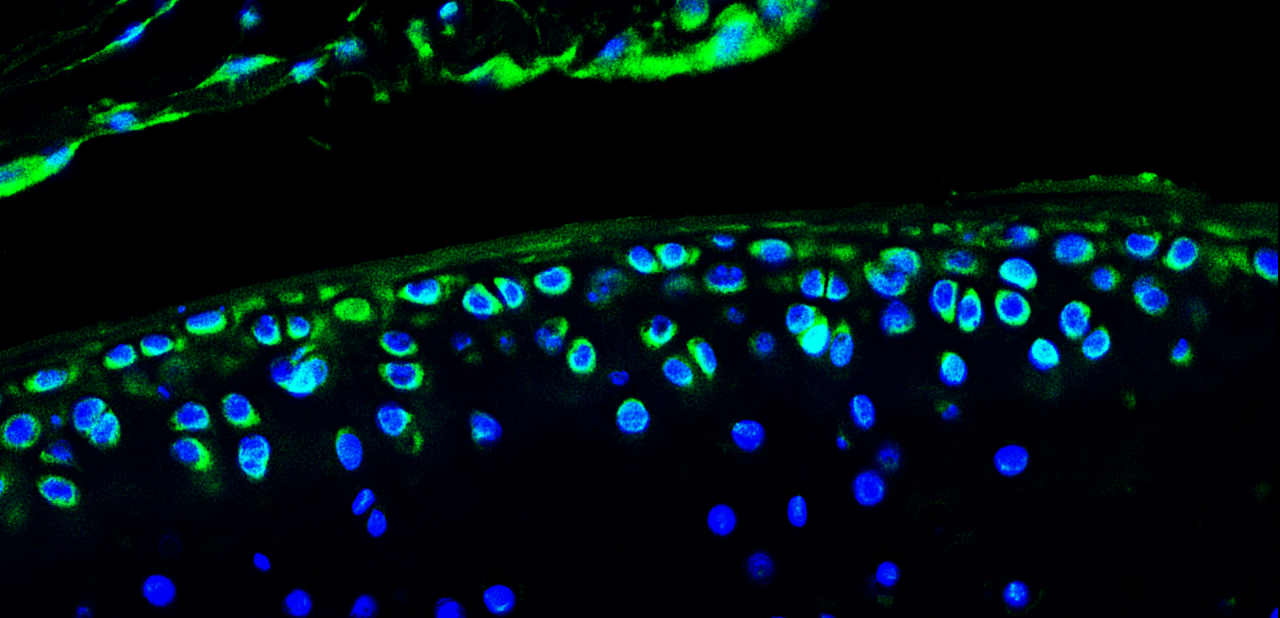


sham+AAV circGNB1 circGNB1


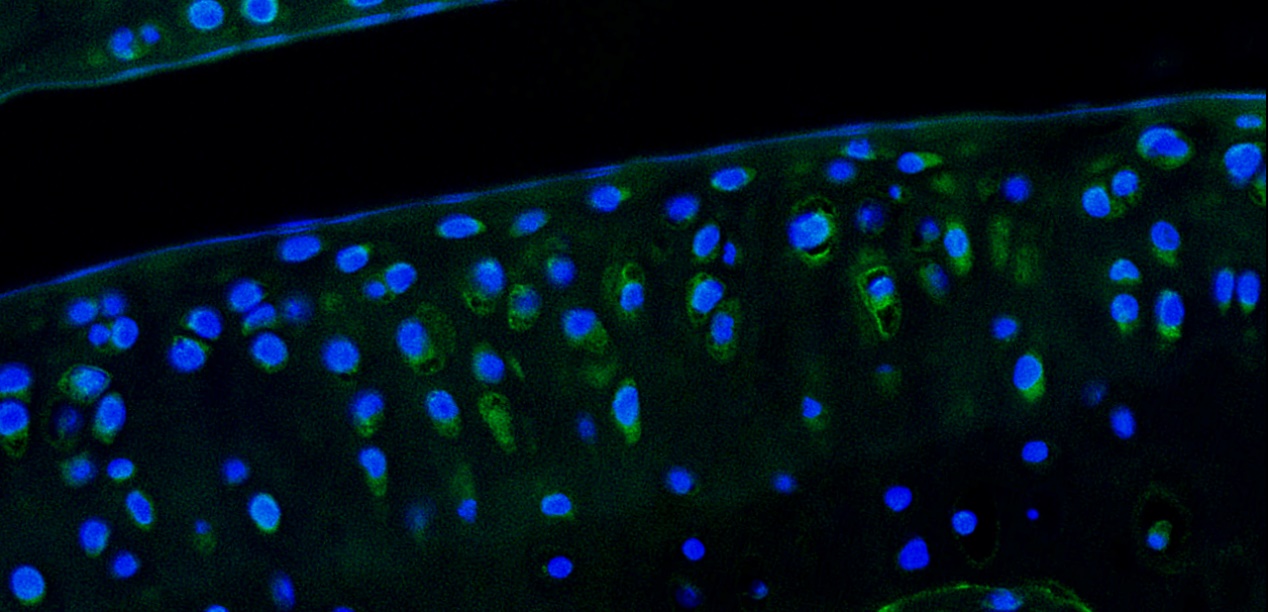


DMM circGNB1


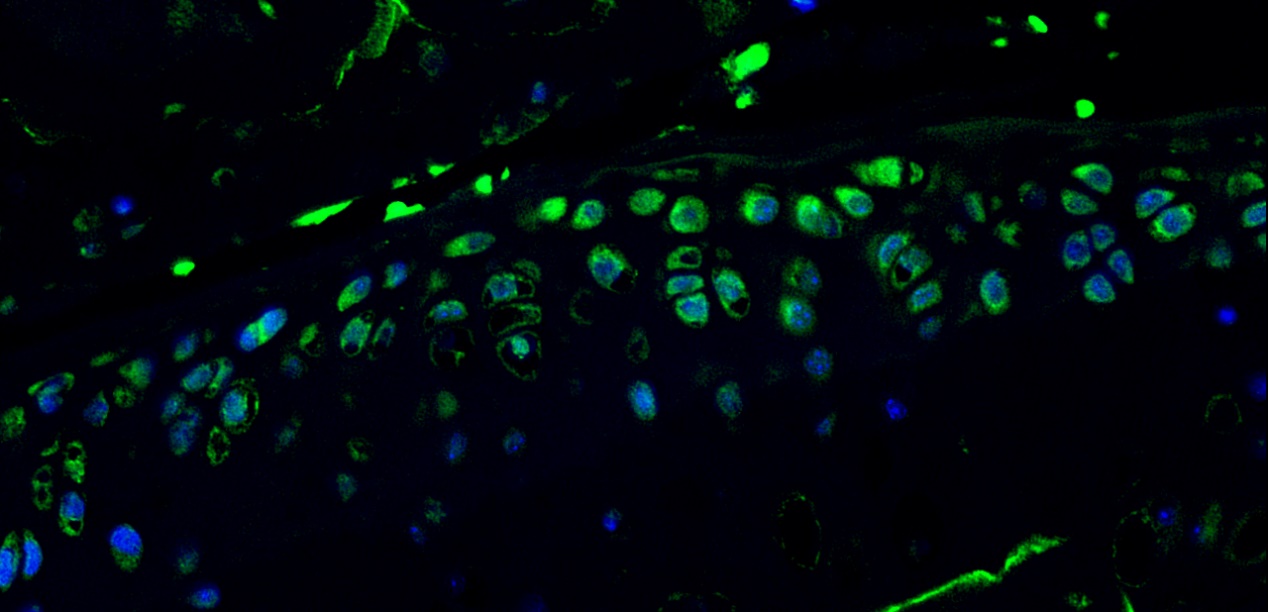


DMM+AAV circGNB1 circGNB1


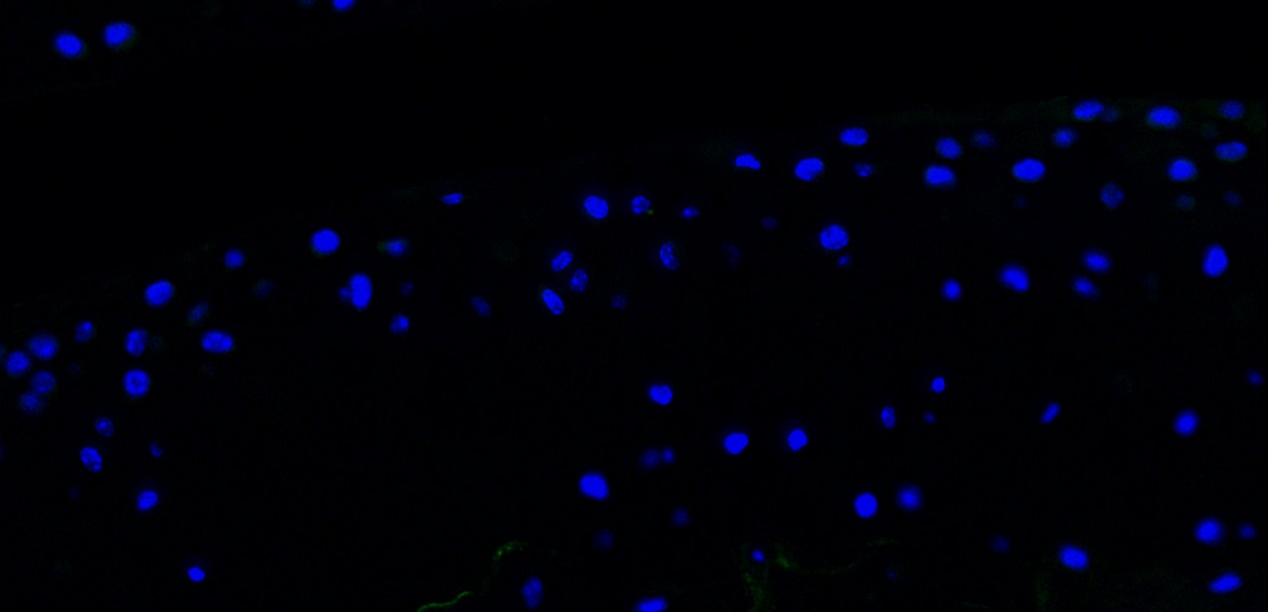


DMM+AAV sh-mmu-circGNB1 circGNB1


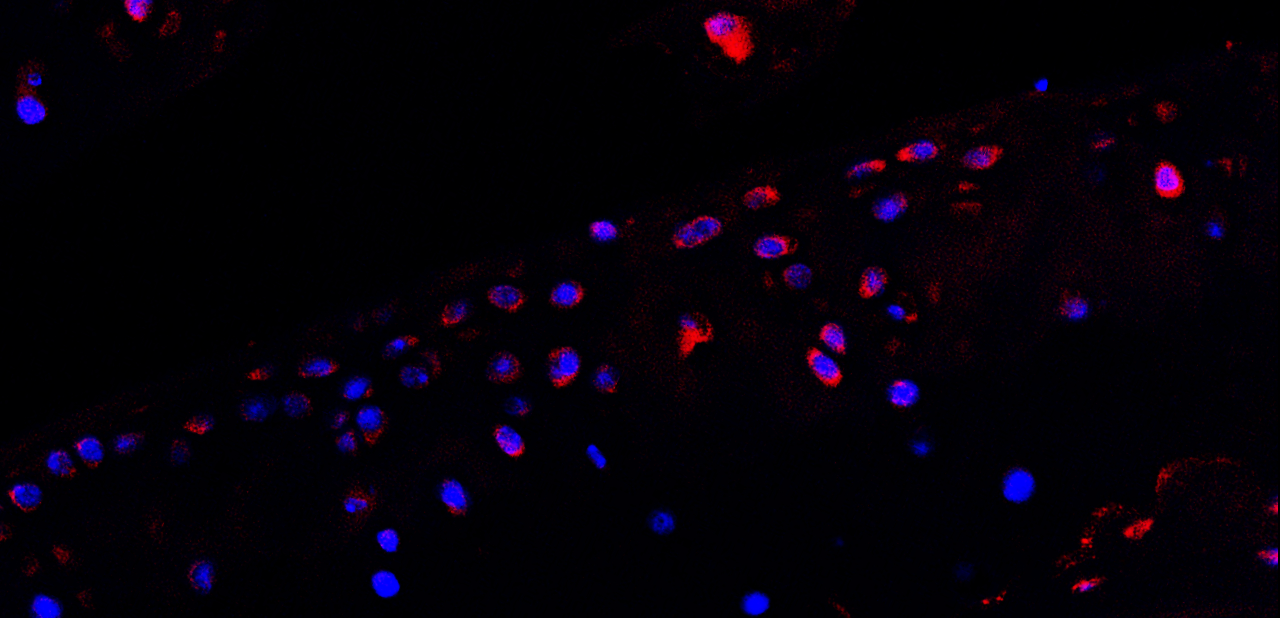


sham miR-152-3p


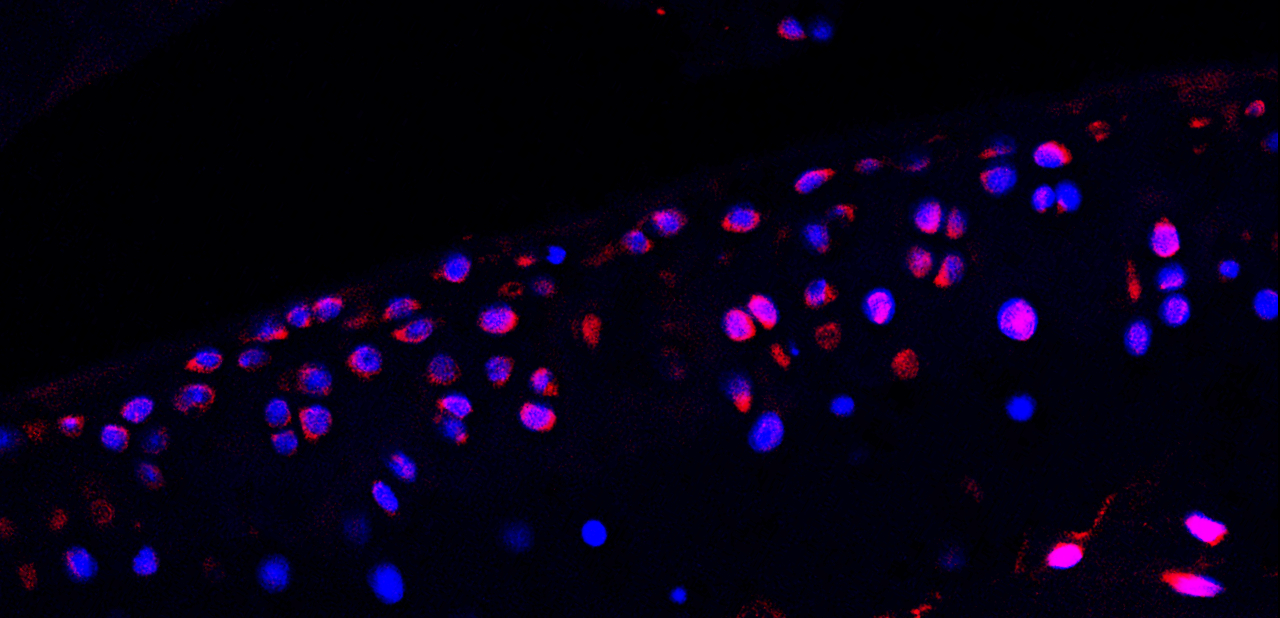


sham+NC miR-152-3p


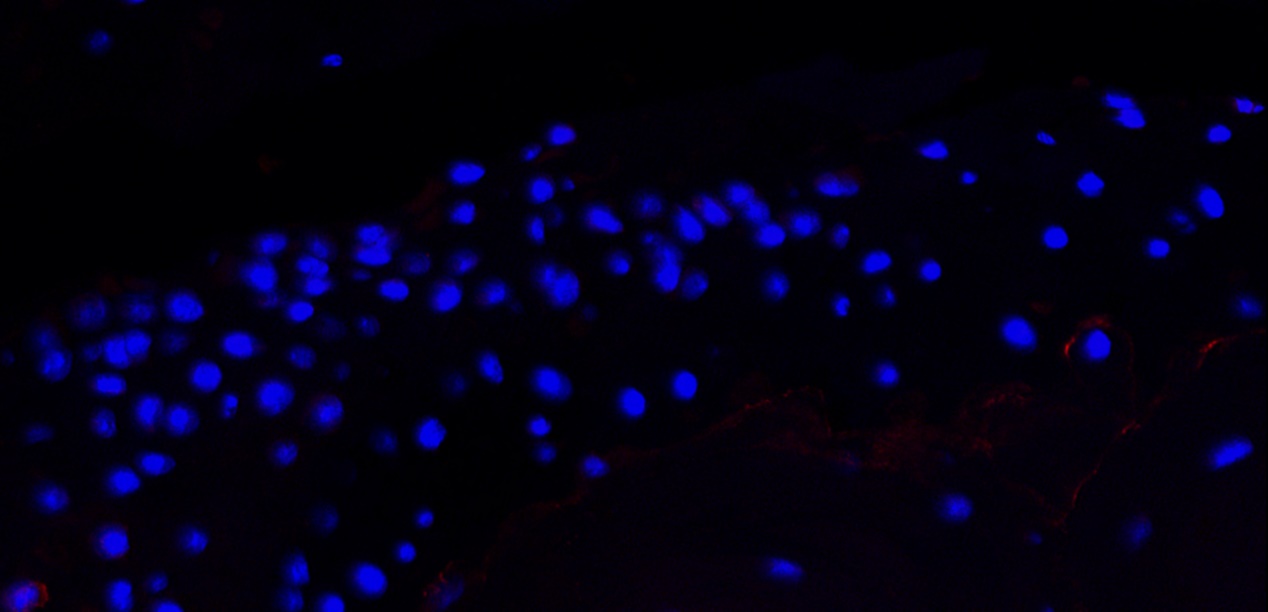


sham+AAV circGNB1 miR-152-3p


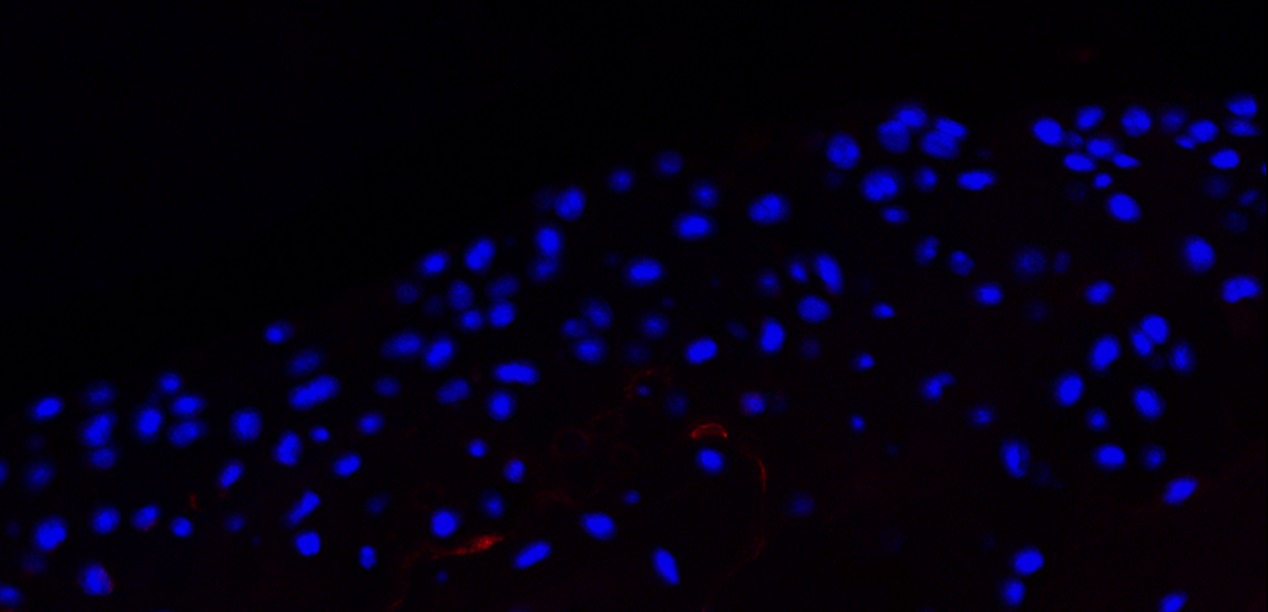


DMM miR-152-3p


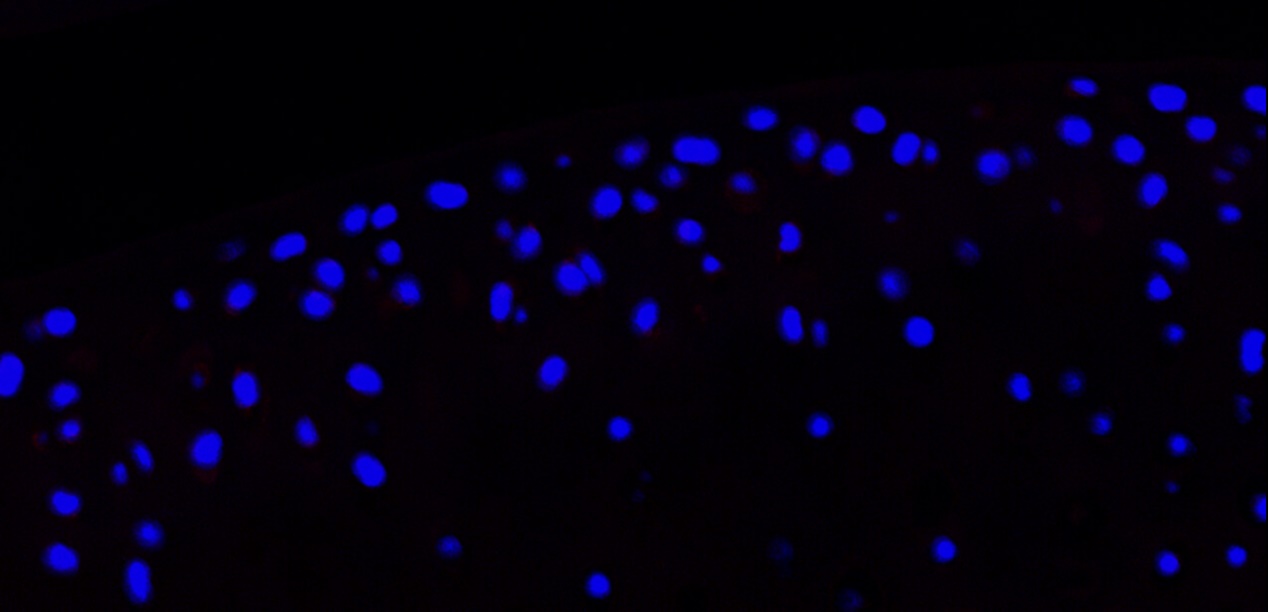


DMM+AAV circGNB1 miR-152-3p


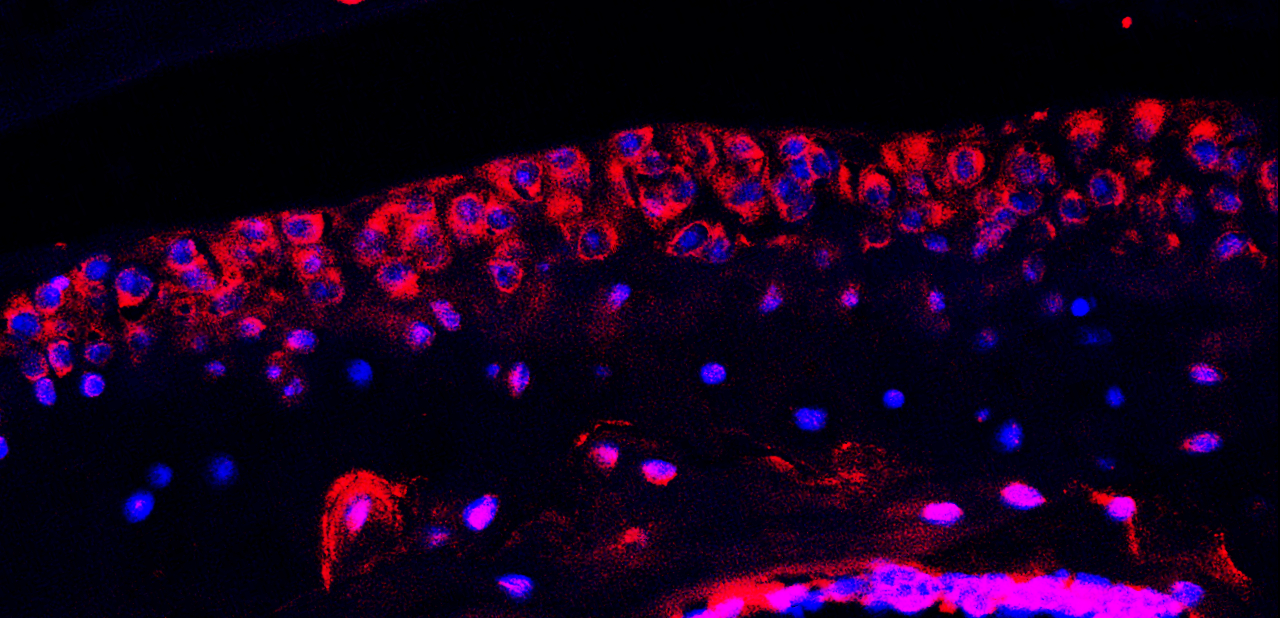


DMM+AAV sh-mmu-circGNB1 miR-152-3p

**Supplementary Figure1G**

**
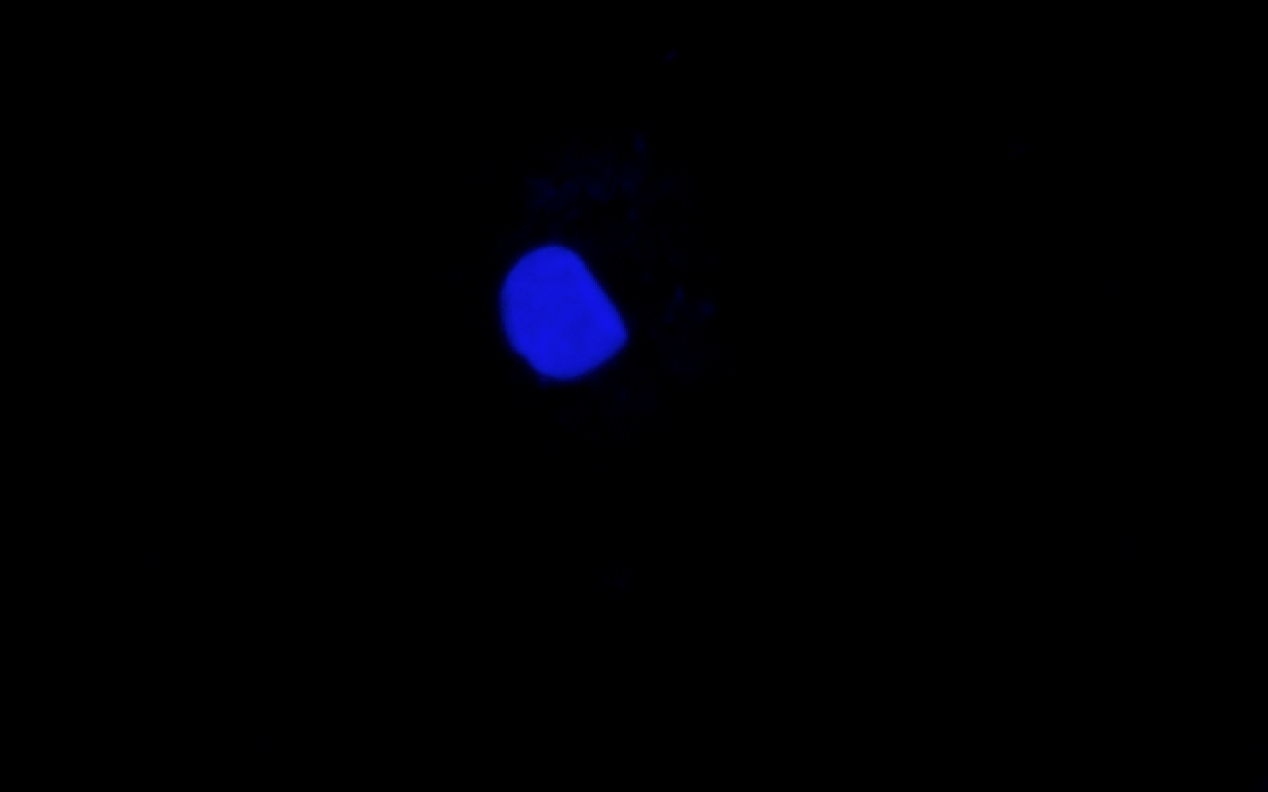
**

DAPI


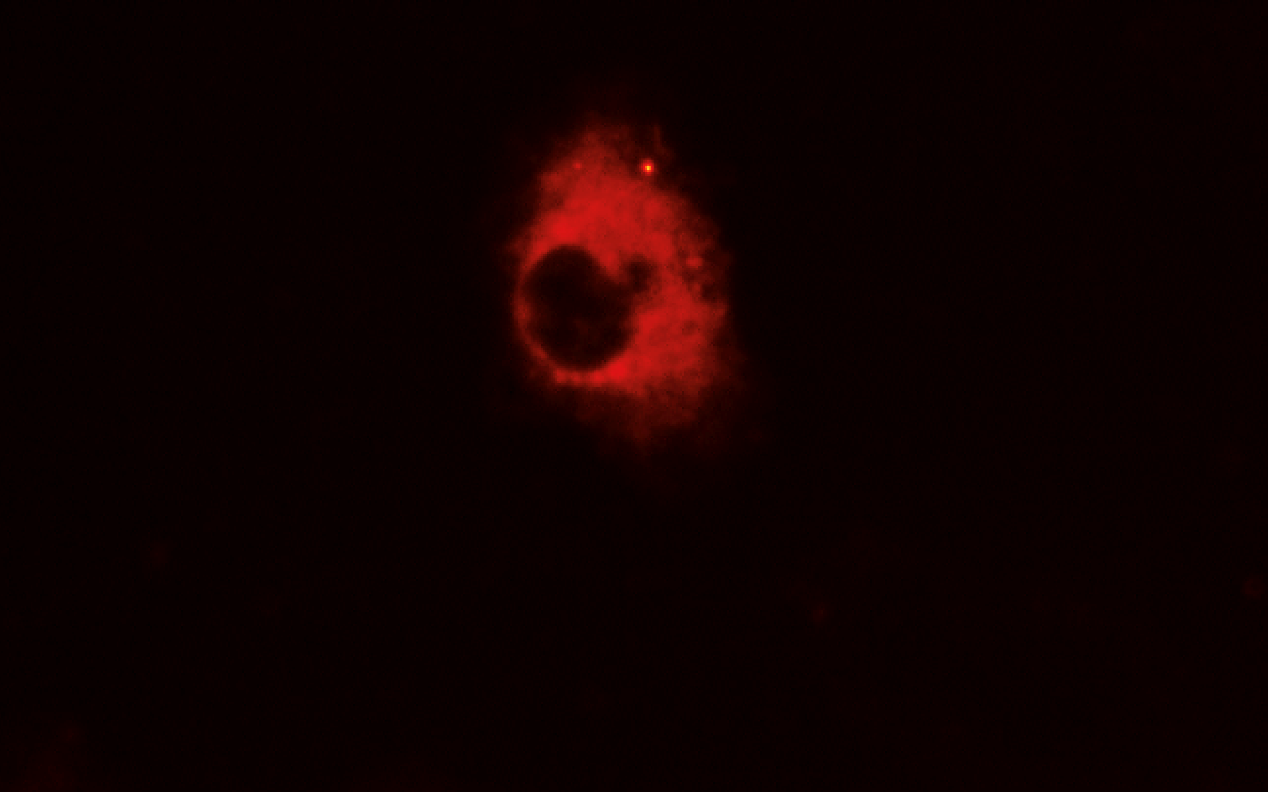


circGNB1


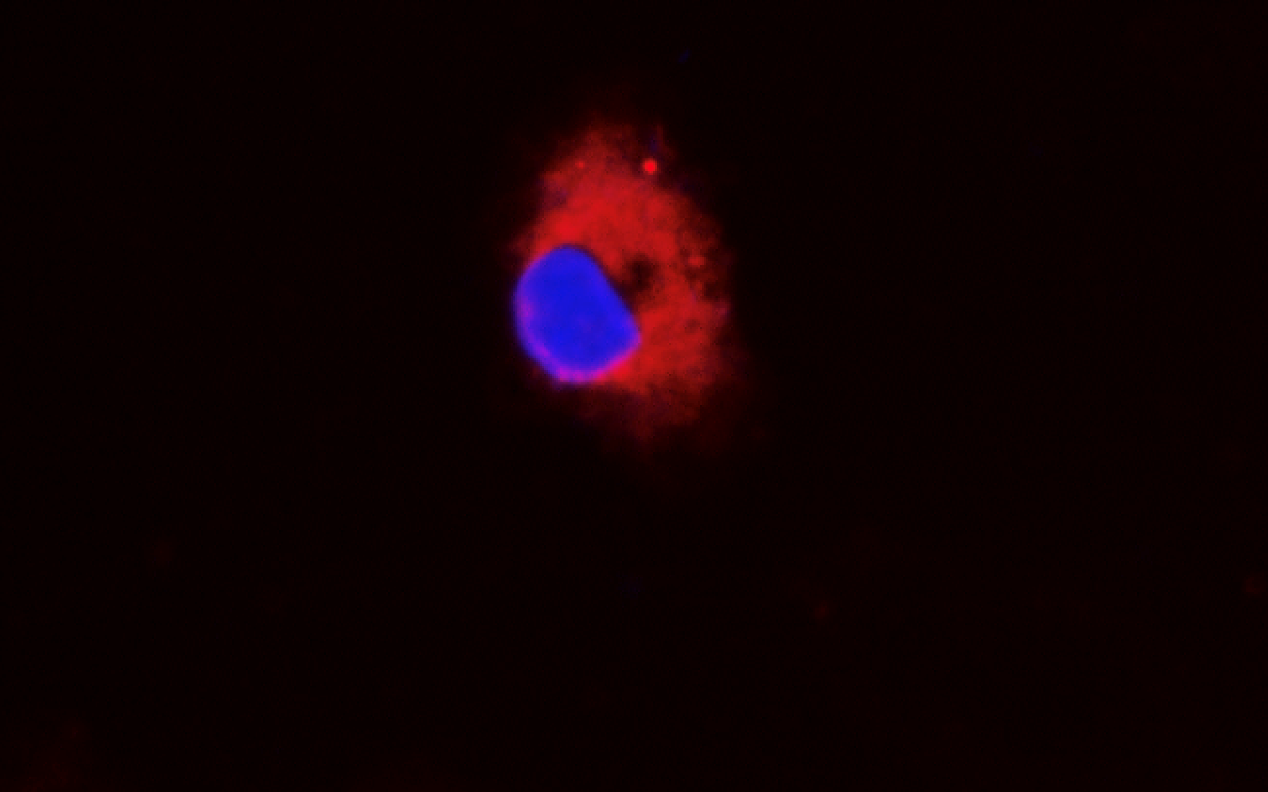


Merge

**Supplementary Figure3A**

**
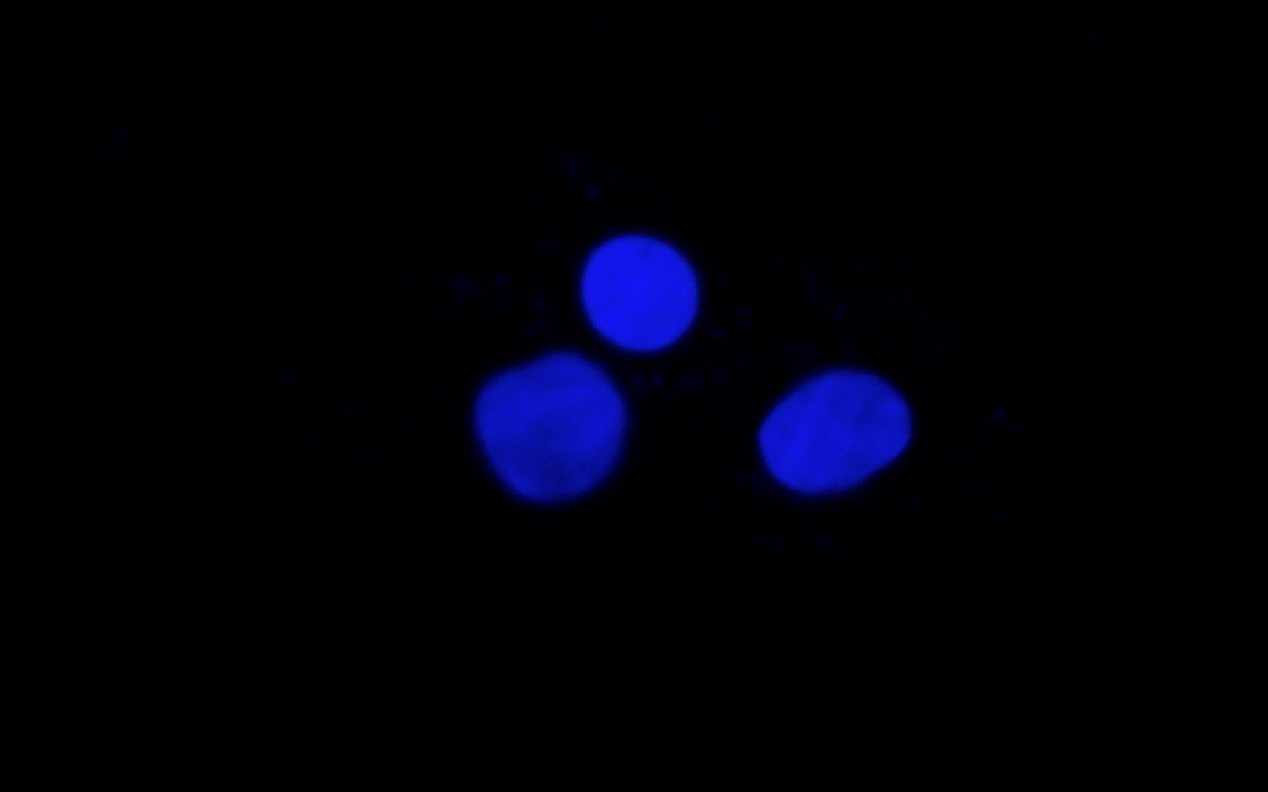
**

DAPI


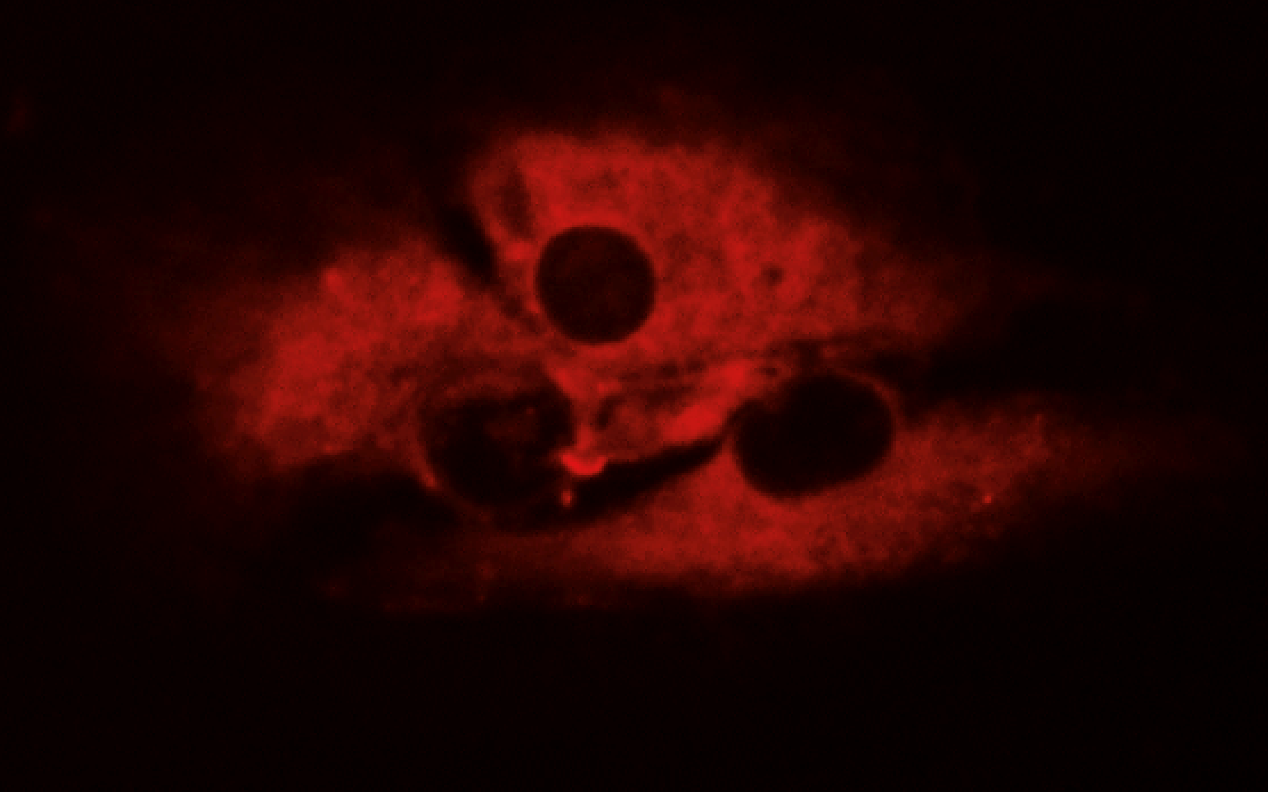


circGNB1
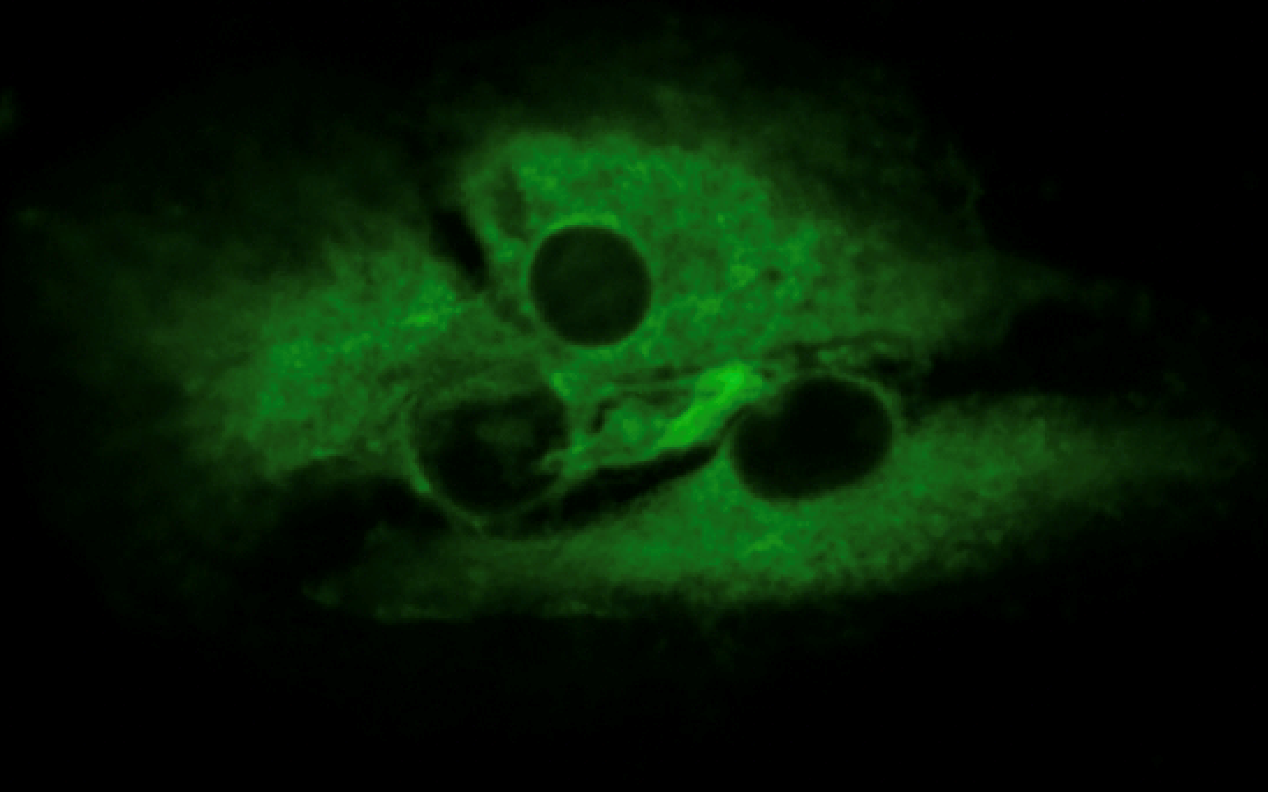


miR-152-3p


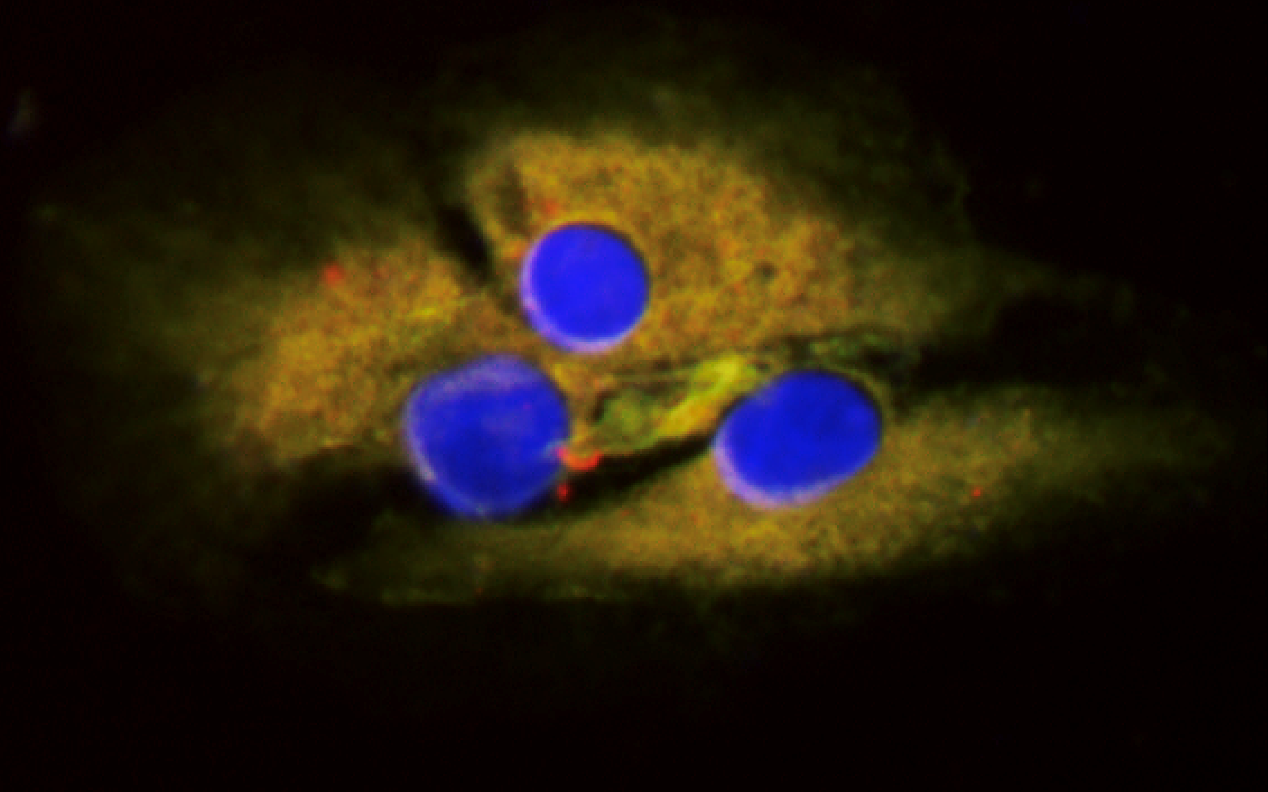


Merge
