# Supplementary material for: CircGNB1 drives osteoarthritis pathogenesis by inducing oxidative stress in chondrocytes
Source: Clin Transl Med. 2023 Aug 3;13(8):e1358. doi: 10.1002/ctm2.1358 (PMC10400757; doi:10.1002/ctm2.1358)
Supplement: Supplementary file 3 — Supporting Information [file CTM2-13-e1358-s002.docx]

**CircGNB1 drives osteoarthritis pathogenesis by inducing oxidative stress in chondrocytes**

Yi Liang^#^, Lifeng Shen^#^, Weiyu Ni^#^, Yuhong Ding, Wentao Yang, Tianyuan Gu, Chenfeng Zhang, Jasper H. N. Yik, Dominik R. Haudenschild, Shunwu Fan, Shuying Shen, Ziang Hu

^#^These authors contributed equally to this work.

**Supplementary Materials and Methods**

- 1. *RNA sequencing*

Total RNA was extracted from a mixture of primary chondrocytes (n=5 patients) treated with 500 μM of H_2_O_2_ for five days (H_2_O_2_-MIX). Untreated chondrocytes were used as negative controls (NC-MIX). The samples were subjected to RNA sequencing to identify circRNAs as described previously.^1^ Differentially expressed circRNAs were defined as having a log2 fold-change greater than 1, and a false discovery rate of less than 0.05. Expression of candidate circRNAs was validated by quantitative real-time PCR (qRT-PCR).

- 1. *RNA extraction, reverse transcription, and qRT-PCR*

A gDNA Reagent Kit (Sangon Biotech, Shanghai, China) was used to isolate gDNA. TRIzol Reagent (Invitrogen, USA) was used to extract total RNA from cartilage tissues and HCs, according to the manufacturer's instructions. A PrimeScript RT Reagent Kit (Accurate Biology, China) was used to synthesize cDNA from 1g of total RNA. To evaluate the expression levels of candidate genes, qRT-PCR was performed in an ABI 7900HT System (Applied Biosystems, USA) with a SYBR Green PCR Kit (Yeasen Biotechnology, China). The 2^−△△CT^ method was used to calculate the relative miRNA and circRNA/mRNA expression levels normalized to those of U6 and β-actin, respectively.

- 1. *circRNA plasmid construction*

The vector named pHBAAV-CMV-circRNA-EF1-ZsGreen (HanBio ,Shanghai, China) was cut by Xho enzyme. Then the circGNB1 primer with homologous arm was designed and cloned by PCR. The vector containing circGNB1 was recombinated using the recombinant enzyme. Cyclization can be driven by reverse complementary pairing of circRNA flanking sequence (Alu structure). Loop-forming elements (Alu structure) were inserted at both ends of the multi-cloning site of the vector to drive the cyclization of the target circGNB1. After the vector was constructed, the transient transfected cells were detected by qRT-PCR to verify the overexpression efficiency.

- 1. *Mouse OA models*

All animal experiments were carried out with the approval of the Institute of Health Sciences Institutional Animal Care and Use Committee (Zhejiang, China). In vivo tests were performed on 8-week old male C57BL/6 mice (n = 60). The positive control in a prior study was medial meniscus destabilization (DMM) surgery, that was used to establish post-traumatic OA.^2, 3^ Mice were randomly divided into six groups (n = 10/group): 1) Sham, 2) Sham + AAV negative control (NC), 3) Sham + AAV circGNB1, 4) DMM, 5) DMM + AAV circGNB1, and 6) DMM + AAV si-mmu_circGNB1. Briefly, animals were anesthetized and the both medial joint capsules were incised. The medial meniscotibial ligament (MMTL) were then cut using microsurgical scissors to destabilize the medial meniscus, followed by flushing with sterile saline and closure of the incision with sutures. As sham operations, the medial joint capsule was incised without the transaction of the MMTL. Adeno-associated viruses (AAVs) harboring circGNB1, sh-mmu_circGNB1, miR-152-3p, RNF219 sh RNA or NC were generated from HanBio (Shanghai, China). A total of 10 μL (about 1 x 10^12^ vg/mL) of AAV NC, AAV circGNB1, AAV si-mmu_circGNB1, AAV miR-152-3p or AAV RNF219 shRNA were injected intra-articularly into the knee joint one week post operation. Mice were then sacrificed after two months and both knee articular cartilages were collected for histological analysis.

- 1. *Measurement of intracellular ROS levels*

A reactive oxygen species assay kit (Beyotime, Shanghai, China) was used to measure intracellular ROS levels according to the manufacturer’s instruction. After 48-hour treatment with stimulants, chondrocytes were given 2 mL of serum-free DMEM supplemented with 10 μM 2,7-Dichlorodihydrofluorescein diacetate (DCFH-DA) and incubated at 37 °C for 20 minutes. Dichlorodihydrofluorescein (DCF), the fluorescent metabolite of DCFH-DA oxidized by intracellular ROS, was detected in chondrocytes using a flow cytometer (Beckman,CytoFLEX, USA). The data was analyzed with FlowJo v10.

- 1. *Immunofluorescence*

HCs were fixed on slides using 4 % paraformaldehyde for 30 minutes and then washed three times with PBS for two minutes each. Cells were covered with PBS containing 0.5 % Triton X-100 for 15 minutes, and then incubated with 5 % BSA in PBS for 1 hour at room temperature. Cells were then incubated overnight at 4 °C with the following primary antibodies at 1:100 dilutions in PBS: anti-MMP13 (18165-1-AP, Proteintech, Wuhan, China), anti-aggrecan (13880-1-AP, Proteintech, Wuhan, China), or anti-Col2a1 (ab34712, Abcam, Cambridge, UK). Cells were then washed three times with PBS, followed by incubation with secondary antibodies at room temperature for 1 hour: DyLight 488 AffiniPure goat anti-rabbit IgG (H&L) (FD0136, Fudebio, HangZhou, China) or DyLight 594 AffiniPure goat anti-rabbit IgG (H&L) (FD0129, Fudebio, HangZhou, China). The nuclei were stained with DAPI for 5 minutes. Target proteins were visualized by a fluorescence microscope (Carl Zeiss, USA).

Immunofluorescence pictures taken in the same batch of experiments under the same conditions were selected. The cell density in the specified area of different groups was the same. Then the pictures were dragged into ImageJ software and converted to gray format picture format. Ten background and cell boxes were selected in turn and measured. The final fluorescence intensity was calculated by IntDen (correction) = IntDen (cell)-Area (cell) * Mean (background).

- 1. *Immunohistochemistry (IHC)*

The sections were incubated with 3% H_2_O_2_ deionized water at room temperature for 5-10min to eliminate endogenous peroxidase activity. Then the sections were incubated with sodium citrate antigen retrieval solution (Boster, California, USA) at 60°C overnight after being washed three times with PBS. The sections were blocked with 5% bovine serum albumin (BSA) in PBS for 30min at 37°C. Sections were incubated with antibodies against MMP13, Aggrecan, CAV1 (at 1:100 dilution, Abcam), RNF219 (at 1:100 dilution, Santa Cruz Biotechnology, California, USA) or IL-10RA (at 1:100 dilution, ABclonal technology, Wuhan, China) at 4°C overnight. After being washed three times with PBS, the sections were incubated with HRP-conjugated goat anti-rabbit secondary antibodies (Boster, California, USA) for 30min at 37°C and were then washed 3 times with PBS. The sections were developed with DAB chromogenic solution (Boster, California, USA) for 1 min at room temperature and then were re-stained with Mayer' Hematoxylin solution (Boster, California, USA) for 2 min at room temperature. Images of sections were acquired using a microscope (CX33TRF, Olympus, Tokyo, Japan). Quantitative analysis adopted blind method，which evaluation of the proportion of positive cells by Image-Pro Plus software.

- 1. *Fluorescence In situ Hybridization (FISH)*

CircGNB1 probes with CY3 labeling and miR-152-3p probes with FAM labeling were designed and produced by RiboBio (Guangzhou, China). A FISH kit (RiboBio, Guangzhou, China) was used to detect probe signals in HCs as per the manufacturer's instructions. Fluorescence images were captured with a Nikon A1Si Laser Scanning Confocal Microscope (Nikon Instruments Inc., Japan. Images were processed with K-Viewer and data were reported as relative fluorescence intensity.

- 1. *RNA immunoprecipitation (RIP)*

The Magna RIP RNA-Binding Protein Immunoprecipitation kit (Millipore, Billerica, MA, USA) was used to perform RNA immunoprecipitation (RIP) experiments. HEK-293T cells (BinSuiBio, Shanghai, China) maintained in DMEM, 10% FBS were seeded onto 100 mm dish. The argonaute-2 (AGO2) plasmid or empty vector (HanBio, Shanghai, China) was transfected into cells using PEI transfection reagents (Sigma-Aldrich, St. Louis,MO, USA) After 2 days, approximately 1x10^7^ cells were pelleted by centrifugation and then resuspended in 100 μL of RIP Lysis Buffer containing RNase inhibitors and protease inhibitors. The cell lysates were then incubated with rabbit anti-IgG or anti-AGO2 (Millipore, Boston, USA) antibodies overnight at 4 °C on a rotatory platform. The lysates were then treated with proteinase K buffer, and the immunoprecipitated RNA was extracted with the RNeasy MinElute Cleanup kit (Qiagen), followed by reverse transcription using a PrimeScript RT Reagent Kit (AGbio) to generate cDNA. CircGNB1 levels was quantified by qRT-PCR and the data was presented as a percentage of the total input materials.

- 1. *RNA antisense purification (RAP)*

RNA antisense purification experiments were performed using the RNA antisense purification technology kit (BersinBio,Guangzhou, China). Briefly, 1x10^7^ human chondrocytes were collected, lysed, and sonicated. To create probe-coated beads, C-1 magnetic beads were incubated with an oligo or circGNB1 probe (RiboBio, Guangzhou, China) at 25 °C for 2 h. To pull down circGNB1, these probe-coated beads were left in contact with the cell lysates at 4 °C overnight. RNA complexes attached to the beads were extracted using an RNeasy Mini kit (QIAGEN, Dusseldorf, Germany). The samples were then further analyzed by qRT-PCR.

- 1. *Knock-down or overexpression*

Small interfering RNAs (siRNAs) were designed and constructed by RiboBio (Guangzhou, China). These include siRNAs that target the circGNB1 back splicing junction (si circGNB1), the specific site of Ring finger protein 219 (si RNF219), unconventional myosin-XVIIIa (si MYO18A), Plectin (si PLEC), Caveolin-1 (si CAV1), Clathrin heavy chain 1 (si CLTC), UDP-glucose 6-dehydrogenase (si UGDH), and the mimic or inhibitor of miR-152-3p (miR-152-3p mimic, miR-152-3p inhibitor). miRNA mimics work by mimicking miRNAs. miRNA inhibitors work by attaching to the complementary strand of the miRNA. HCs were seeded at a density of 2x10^5^ cells per well in 6-well plates and then transfected with miR-152-3p mimic/inhibitor or siRNAs using Lipofectamine RNAiMAX (Thermo Fisher, Waltham, USA) according to the manufacturer's instructions.

The same siRNA sequences were used to generate lentiviral constructs harboring short hairpin RNA (shRNA) to establish stable downregulation or overexpression of circGNB1, RNF219, and CAV1 in HCs. HCs were infected with 1×10^8^ TU/ml titer of different lentivirus in the presence of 10 μg/ml polybrene (Solarbio, Beijing, China).

The transient transfection protocol of the HC: HCs were seeded at a density of 2x10^5^ cells per well in 6-well plates and then transfected with 500ng plasmids by PEI transfection reagents per well (Sigma-Aldrich, St. Louis,MO, USA).

- 1. C*o-immunoprecipitation (co-IP)*

Co-IP was performed using the cell lysates with antibodies specific for HA (1:1000, 4970s, Cell Signaling Technology), Myc (1:1000, ab32, abcam), FLAG (1:1000, ab205606, abcam), RNF219 (1:1000, ab91464, abcam), CAV1 (1:5000, ab192869, abcam) or anti-Ubiquitin antibody (1:1000, ab140601, abcam). Proteins attached to beads were released by

protein heat-denature and detected by Western blot with appropriate secondary antibody and a chemiluminescence system (Bio-Rad, USA).

- 1. *Micro-CT analysis*

All knee joints samples fixed in paraformaldehyde were scanned using a high-resolution μCT (Skyscan 1072; Skyscan, Aartselaar, Belgium) in 17 mm scanning tubes with 11 mm^3^ volume at 180 mA, 55,000 V, and an acquisition time of 115-minute. Data was analyzed with Skyscan software (VGStudio MAX; Volume Graphics, Heidelberg, Germany).

- 1. *Dual-luciferase reporter assay*

Luciferase reporter plasmids and miRNAs mimics were generated by Genechem (Shanghai, China). Wild-type or mutant circGNB1 and RNF219 fragments were inserted into the Xba1 restriction site of the firefly luciferase-Renila luciferase vector (hFLuc-XbaL-hRLuc) to generate plasmid (Luc-circGNB1 WT or Luc-circGNB1 Mut) and (Luc-RNF219 WT or Luc-RNF219 Mut). HEK-293T cells seeded in 96-well plates at 50%-70% confluency were co-transfected with various luciferase reporter plasmids, and different miRNA mimics by PEI transfection reagents (Sigma-Aldrich, St. Louis,MO, USA). The luciferase activity was measured after 48 hours, using the Luciferase Assay Reagent (Yeason, Shanghai, China). Relative luciferase activities were determined by calculating the ratio between firefly and Renilla luciferase activities.

- 1. *Western blot*

Chondrocytes were lysed using RIPA lysis buffer (Beyotime, Shanghai, China). Cell lysates were subjected to SDS-PAGE and the protein bands transferred to polyvinylidene fluoride (PVDF) membranes (Sigma-Aldrich, St. Louis,MO, USA). After blocking with 5% BSA (Beyotime, Shanghai, China) in TBST for one hour, the membranes were incubated with various primary antibodies overnight at 4 °C. It was then washed and incubated with the appropriate secondary antibodies for one hour. Protein bands were detected using the FDbio-Femto ECL kit (Fudebio, Hangzhou, China) and the images captured using the chemiluminescence system (Bio-Rad, USA). Signal intensities were quantified using Image J software. The antibodies used and their dilutions were: anti-β-actin (1:1000, 4970s, Cell Signaling Technology), anti-MMP3 (1:1000, ab52915, Abcam), anti-MMP13 (1:1000, ab51072, Abcam), anti-SOX9 (1:1000, ab185966, Abcam), anti-Aggrecan (1:1000, ab3778, Abcam), anti-COL2A1 (1:1000, ab34712, Abcam), anti-RNF219 (1:1000, ab91464, Abcam), anti-CAV1 (1:5000, Abcam, ab192869, Abcam), anti-FLAG (1:1000, ab205606, Abcam), anti-Myc (1:1000, ab32, Abcam), anti-HA (1:1000, 4970s, Cell Signaling Technology), anti-Ubiquitin (1:1000, ab140601, Abcam), anti-Phospho-Stat3 (1:1000, #9145, Cell Signaling Technology), JAK1 (1:1000, ab133666, Abcam), p-JAK1 (1:1000, ab138005, Abcam), STAT3 (1:1000, ab68153, Abcam).

- 1. *β-galactosidase (β-gal) staining*

Senescence associated (SA)-β-gal assay was performed using Senescence β-Galactosidase Staining Kit (Beyotime, Shanghai, China) according to the manufacturer’s instruction. HCs seeded on microscope slides were fixed in 4 % paraformaldehyde and then stained with the β-gal staining solution overnight at 37°C. Images were capture using a microscope (CX33TRF, Olympus, Tokyo, Japan). Senescent cells were counted using Image J.

- 1. *Nuclear cytoplasmic separation experiment*

Nuclear cytoplasmic separation experiment was performed by cytoplasmic nuclear RNA Purification Kit (AmyJet Scientific, WuHan, China). 1x10^7^ human chondrocytes suspension was transfered to a tube and centrifuged at 1000rpm, and then pour out the supernatant. Adding 200uL Lysis Buffer J to the precipitate and centrifuge at 5000rpm for 10 minutes. Next, the supernatant containing cytoplasmic RNA was transfered to another tube with the precipitate contains cytosolic RNA fraction. The cytoplasmic RNA and nuclear RNA were then purified by binding them to the spin columns, respectively, followed by reverse transcription using a PrimeScript RT Reagent Kit (AGbio) to generate cDNA. CircGNB1 levels was quantified by qRT-PCR.

- 1. *Histological analysis*

Human cartilage or mouse knee specimens were fixed in 4 % paraformaldehyde and then embedded in paraffin. Samples were cut into 5 μm sections and stained with 0.1% Safranin O solution and 0.001% Fast Green solution (Sigma-Aldrich, St. Louis,MO, USA). Severity of OA in human samples was assessed by two blinded observers using the OARSI grading system.^4, 5^ The maximal score of observed cartilage was used to measure the severity of OA.

**References**

1. Yang, Y.; Shen, P.; Yao, T.; Ma, J.; Chen, Z.; Zhu, J.; Gong, Z.; Shen, S.; Fang, X., Novel role of circRSU1 in the progression of osteoarthritis by adjusting oxidative stress. *Theranostics* **2021,** *11* (4), 1877-1900.

2. Glasson, S. S.; Blanchet, T. J.; Morris, E. A., The surgical destabilization of the medial meniscus (DMM) model of osteoarthritis in the 129/SvEv mouse. *Osteoarthritis and cartilage* **2007,** *15* (9), 1061-9.

3. Chen, D.; Shen, J.; Zhao, W.; Wang, T.; Han, L.; Hamilton, J. L.; Im, H. J., Osteoarthritis: toward a comprehensive understanding of pathological mechanism. *Bone research* **2017,** *5*, 16044.

4. Glasson, S. S.; Chambers, M. G.; Van Den Berg, W. B.; Little, C. B., The OARSI histopathology initiative - recommendations for histological assessments of osteoarthritis in the mouse. *Osteoarthritis and cartilage* **2010,** *18 Suppl 3*, S17-23.

5. Pritzker, K. P.; Gay, S.; Jimenez, S. A.; Ostergaard, K.; Pelletier, J. P.; Revell, P. A.; Salter, D.; van den Berg, W. B., Osteoarthritis cartilage histopathology: grading and staging. *Osteoarthritis and cartilage* **2006,** *14* (1), 13-29.
