# Supplementary material for: CircGNB1 drives osteoarthritis pathogenesis by inducing oxidative stress in chondrocytes
Source: Clin Transl Med. 2023 Aug 3;13(8):e1358. doi: 10.1002/ctm2.1358 (PMC10400757; doi:10.1002/ctm2.1358)
Supplement: Supplementary file 4 — Supporting Information [file CTM2-13-e1358-s001.docx]

**CircGNB1 drives osteoarthritis pathogenesis by inducing oxidative stress in chondrocytes**

Yi Liang^#^, Lifeng Shen^#^, Weiyu Ni^#^, Yuhong Ding, Wentao Yang, Tianyuan Gu, Chenfeng Zhang, Jasper H. N. Yik, Dominik R. Haudenschild, Shunwu Fan, Shuying Shen, Ziang Hu

^#^These authors contributed equally to this work.

**Supplementary tables**

**Table S1 Primers used in the study**

| **Gene** | **Species** | **Primer** |
| --- | --- | --- |
| β-actin-F | human | AGAGCTACGAGCTGCCTGAC |
| β-actin-R | human | AGCACTGTGTTGGCGTACAG |
| hsa_circGNB1-F | human | AGAATCCAAATGCGCACGAG |
| hsa_circGNB1-R | human | GAAGGGCTTCTGGTCTGGTC |
| hsa_circ_0005354-F | human | ATCATGTGGCTGGACCATCG |
| hsa_circ_0005354-R | human | GGTCTGCAAAAGCCAACAGG |
| hsa_circ_0006173-F | human | CCAGACAGGACTTTCTTCTGCT |
| hsa_circ_0006173-R | human | TGTGGTCAGAATCAGGGCCTA |
| hsa_circ_0000607-F | human | AGAAATTCAGACTGCAAATGAACA |
| hsa_circ_0000607-R | human | GCAATCCAGTTTGGGCGTTT |
| hsa_circ_0002220-F | human | AGGGATTCTGTAGCCCCAAAC |
| hsa_circ_0002220-R | human | GATCCAGGCCCTCTTTGATGA |
| hsa_circ_0006646-F | human | TTCACGTGGATATGGGCGTC |
| hsa_circ_0006646-R | human | AGTTGGGGTCAAGGTAAGCAG |
| hsa_circ_0003391-F | human | ACACAACATCTTCCCCCACA |
| hsa_circ_0003391-R | human | GAAGGGGTCACCGACATGG |
| hsa_circ_0002431-F | human | AAGGTAGCAGTGACACCTCC |
| hsa_circ_0002431-R | human | TTGACTTGCAATCCTCAGAGAGT |
| hsa_circ_0001472-F | human | TGAGCGAATAGAGAGAGAATCAGC |
| hsa_circ_0001472-R | human | TCAGGTGCCAGCTGTCATTA |
| hsa_circ_0002359-F | human | TGTTGCTGCTGATGAAGACGTT |
| hsa_circ_0002359-R | human | GATCAGGGTCTGGGCGAACTA |
| hsa_circ_0001495-F | human | TGGTGAATGGAATAATTGTGTGCC |
| hsa_circ_0001495-R | human | AGTCACCAATTTCTGGAGGGT |
| hsa_circ_0001447-F | human | AAAAGAACTGCTGCCCAGTGA |
| hsa_circ_0001447-R | human | ATCGAGACATGGGAGTCCTG |
| hsa_circ_0007312-F | human | CAAGATTAGCACAGATTTGCTTGTA |
| hsa_circ_0007312-R | human | TTGCAGACACTTTAGGCACTGA |
| hsa_circ_0000836-F | human | AAGCTGGAGCAGATGTTACC |
| hsa_circ_0000836-R | human | GCACACTTCCATCGAATGCC |
| hsa_circ_0001861-F | human | GATCCGAGTTGGCTACACCC |
| hsa_circ_0001861-R | human | CTCCACCTCACAGTTCTTCACT |
| **Gene** | **Species** | **Primer** |
| hsa_circ_0000257-F | human | GGAGCAGACCAAGGCAGCG |
| hsa_circ_0000257-R | human | CGTCAAAGATCACGACTGTCCC |
| hsa_circ_0008539-F | human | ATGCCGACTCTCATACAGGC |
| hsa_circ_0008539-R | human | TTGATCCGCAAGAGATGCCC |
| hsa_circ_0001788-F | human | GCTAGAAAAAGCATCAAATCCCA |
| hsa_circ_0001788-R | human | GGCTGGGAGATCCCATCAATTT |
| hsa_circ_0001614-F | human | TCATACAGTGCACACTGGTTTTT |
| hsa_circ_0001614-R | human | TCTGTATGACAGCATTTTCATGGT |
| hsa_circ_0008777-F | human | CTGCTACTGCTACAGCTCCA |
| hsa_circ_0008777-R | human | AAGGAATGCGCTGGGAAGAA |
| mGNB1-F | human | AGCTTGACCAGTTACGGCAG |
| mGNB1-R | human | TCCTCGTGCGCATTTGGATT |
| MMP3-F | human | CCTACAAGGAGGCAGGCAAG |
| MMP3-R | human | CCCGTCACCTCCAATCCAAG |
| MMP13-F | human | TCGGCCACTCCTTAGGTCTT |
| MMP13-R | human | AAGTGGCTTTTGCCGGTGTA |
| ADAMTS5-F | human | CCGGAGCCACTGCTTCTATC |
| ADAMTS5-R | human | ACCCCCACAGAGGTCAAAGA |
| SOX9-F | human | GCTCTGGAGACTTCTGAACGA |
| SOX9-R | human | CCGTTCTTCACCGACTTCCT |
| Aggrecan-F | human | AAGGGCGAGTGGAATGATGT |
| Aggrecan-R | human | CGTTTGTAGGTGGTGGCTGTG |
| COL2A1-F | human | CCAGATGACCTTCCTACGCC |
| COL2A1-R | human | TTCAGGGCAGTGTACGTGAAC |
| IL-1β-F | human | TCGCCAGTGAAATGATGGCT |
| IL-1β-R | human | TGGAAGGAGCACTTCATCTGTT |
| TNFα-F | human | TAGCCCATGTTGTAGCAAACC |
| TNFα-R | human | GCTCTTGATGGCAGAGAGGA |
| COX2-F | human | CTGGCGCTCAGCCATACAG |
| COX2-R | human | CGCACTTATACTGGTCAAATCCC |
| iNOS-F | human | TTCAGTATCACAACCTCAGCAAG |
| iNOS-R | human | TGGACCTGCAAGTTAAAATCCC |
| hsa-miR-660-3p | human | CGACCTCCTGTGTGCATGGATTA |
| hsa-miR-7161-3p | human | TAGATCTTTGACTCTGGCAGTCTCCAG |
| hsa-miR-3663-5p | human | TATAGCTGGTCTGCGTGGTGC |
| hsa-miR-764 | human | GCAGGTGCTCACTTGTCCTC |
| hsa-miR-6843-3p | human | CGATGGTCTCCTGTTCTCTGCAG |
| hsa-miR-933 | human | TGTGCGCAGGGAGACCT |
| hsa-miR-99a-3p | human | CCAAGCTCGCTTCTATGGGTCTG |
| hsa-miR-152-3p | human | CCGTCAGTGCATGACAGAACTTGG |
| hsa-miR-6829-3p | human | TATATATGCCTCCTCCGTGGCCTC |
| hsa-miR-3657 | human | CGCGTGTGTCCCATTATTGGTGATT |
| hsa-miR-4757-5p | human | AGGCCTCTGTGACGTCACG |
| **Gene** | **Species** | **Primer** |
| hsa-miR-3074-5p | human | GGTTCCTGCTGAACTGAGCCA |
| hsa-miR-4435 | human | ATGGCCAGAGCTCACACAGAG |
| hsa-miR-1976 | human | CCTCCTGCCCTCCTTGC |
| hsa-miR-219a-1-3p | human | TAGAGTTGAGTCTGGACGTCCCG |
| hsa-miR-5008-3p | human | TATATATACCTGTGCTCCCAGGGCCT |
| hsa-miR-22-5p | human | CGCAGTTCTTCAGTGGCAAGCTTTA |
| hsa-miR-6889-3p | human | TCTGTGCCCCTACTTCCCAG |
| hsa-miR-6761-5p | human | CTCTGAGAGAGCTCGATGGCAG |
| CSF1-F | human | TGGCGAGCAGGAGTATCAC |
| CSF1-R | human | AGGTCTCCATCTGACTGTCAAT |
| DDX6-F | human | ATGGGTCTGTCCAGTCAAAATG |
| DDX6-R | human | GGTGGTCATACTCTGTGCTTG |
| MLEC-F | human | CACAGTCCCAGCAAAAGGTAT |
| MLEC-R | human | ATGCCCAACACGATCAAAGAT |
| SESN3-F | human | CTGGGAAAATCATGGGTTCTCC |
| SESN3-R | human | GCATGGTTGTGTCAACATCCT |
| RNF219-F | human | CAGACCGTGCAGAATGTTACA |
| RNF219-R | human | CAGGCTGACGTACCTTCCC |
| IQGAP1-F | human | AGAACGTGGCTTATGAGTACCT |
| IQGAP1-R | human | CCAGTCGCCTTGTATCTGGT |
| LMNA-F | human | AGCAGCGTGAGTTTGAGAGC |
| LMNA-R | human | AGACTGCCTGGCATTGTCC |
| MYL12A-F | human | GCTTGCTTCATTGGGGAAGAA |
| MYL12A-R | human | CCTGTATGGTGCCAGTTGCT |
| PLEC-F | human | TGTACCGGCAGACCAACCT |
| PLEC-R | human | GCATGGCGTCATACAGCGA |
| MYO18A-F | human | GGACTCGGGCCACCTAAGTA |
| MYO18A-R | human | GGGCGTCGAGGTTTCTGAG |
| CAV1-F | human | GCGACCCTAAACACCTCAAC |
| CAV1-R | human | ATGCCGTCAAAACTGTGTGTC |
| CLTC-F | human | ATTCTGCCAATTCGTTTTCAGGA |
| CLTC-R | human | GCTTTCAGTGCAATTACTTTGCT |
| UGDH-F | human | CCCTGTGTGCTGTATATGAGC |
| UGDH-R | human | TGCTTATTCTCTGGGCAAGAAAA |
| RPS3A-F | human | AGGGTCGTGTGTTTGAAGTGA |
| RPS3A-R | human | CATGGAAGTTAGTCAGGCAGTTT |
| RPL7A-F | human | GGACCGCCAAACAGCTACTC |
| RPL7A-R | human | GGTGGTCTCTTCGTTGGGAC |
| IL10RA-F | human | CCTCCGTCTGTGTGGTTTGAA |
| IL10RA-R | human | CACTGCGGTAAGGTCATAGGA |
| CXCL11-F | human | GACGCTGTCTTTGCATAGGC |
| CXCL11-R | human | GGATTTAGGCATCGTTGTCCTTT |
| IFNB1-F | human | ATGACCAACAAGTGTCTCCTCC |
| **Gene** | **Species** | **Primer** |
| IFNB1-R | human | GGAATCCAAGCAAGTTGTAGCTC |
| IFNL1-F | human | CACATTGGCAGGTTCAAATCTCT |
| IFNL1-R | human | CCAGCGGACTCCTTTTTGG |
| IFNL2-F | human | ACGCGAGACCTGAATTGTGT |
| IFNL2-R | human | AGCGACTGGGTGGCAATAAA |
| CCL5-F | human | CCAGCAGTCGTCTTTGTCAC |
| CCL5-R | human | CTCTGGGTTGGCACACACTT |
| CXCL6-F | human | AGAGCTGCGTTGCACTTGTT |
| CXCL6-R | human | GCAGTTTACCAATCGTTTTGGGG |
| INHBB-F | human | CGGGTCCGCCTATACTTCTTC |
| INHBB-R | human | CGTAGGGCAGGAGTTTCAGG |
| GAPDH-F | human | CTGGGCTACACTGAGCACC |
| GAPDH-R | human | AAGTGGTCGTTGAGGGCAATG |
| U2-F | human | CATCGCTTCTCGGCCTTTTG |
| U2-R | human | TGGAGGTACTGCAATACCAGG |

**Table S2 General Conditions of Patients**

| NO. | Gender | Age | BMI | WOMAC GRADE  (100 points total) | | | OARSI | |
| --- | --- | --- | --- | --- | --- | --- | --- | --- |
|  |  |  |  | Pain | Stiffness | Activity | Lateral | Medial |
| 1 | F | 65 | 26.4 | 11 | 4 | 31 | 0 | 3 |
| 2 | M | 63 | 27.3 | 10 | 3 | 33 | 1 | 4 |
| 3 | F | 61 | 28.2 | 13 | 5 | 37 | 0 | 3 |
| 4 | F | 60 | 23.6 | 10 | 4 | 40 | 2 | 5 |
| 5 | F | 67 | 25.8 | 12 | 5 | 38 | 0 | 4 |
| 6 | M | 66 | 25.4 | 11 | 3 | 37 | 1 | 2 |
| 7 | F | 67 | 27.5 | 9 | 2 | 32 | 0 | 3 |
| 8 | M | 62 | 22.3 | 13 | 4 | 35 | 1 | 1 |
| 9 | M | 68 | 28.2 | 12 | 3 | 31 | 2 | 4 |
| 10 | M | 65 | 27.5 | 10 | 2 | 30 | 2 | 3 |
| 11 | M | 69 | 22.2 | 11 | 2 | 39 | 1 | 5 |
| 12 | F | 64 | 29.7 | 12 | 3 | 37 | 0 | 4 |
| 13 | M | 67 | 28.9 | 9 | 2 | 30 | 1 | 3 |
| 14 | F | 62 | 23.1 | 10 | 2 | 36 | 1 | 3 |
| 15 | F | 67 | 26.4 | 14 | 3 | 32 | 2 | 4 |
| 16 | M | 72 | 29.8 | 19 | 8 | 56 | 4 | 6 |
| 17 | M | 76 | 23.9 | 17 | 6 | 55 | 5 | 6 |
| 18 | F | 70 | 22.5 | 20 | 8 | 59 | 4 | 6 |
| 19 | M | 81 | 22.3 | 18 | 7 | 46 | 3 | 5 |
| 20 | F | 79 | 25.3 | 17 | 6 | 48 | 5 | 6 |
| 21 | M | 83 | 27.9 | 18 | 8 | 58 | 4 | 5 |
| 22 | M | 81 | 24.7 | 18 | 7 | 63 | 3 | 6 |
| 23 | F | 82 | 25.6 | 20 | 8 | 62 | 5 | 5 |
| 24 | M | 77 | 25.9 | 19 | 6 | 51 | 3 | 6 |
| 25 | F | 75 | 23.1 | 20 | 8 | 55 | 4 | 5 |
| 26 | F | 77 | 23.5 | 18 | 6 | 50 | 3 | 6 |
| 27 | F | 73 | 27.8 | 17 | 6 | 58 | 4 | 6 |
| 28 | M | 76 | 23.6 | 19 | 7 | 56 | 3 | 5 |
| 29 | M | 82 | 22.0 | 20 | 8 | 59 | 4 | 5 |
| 30 | F | 74 | 24.4 | 19 | 7 | 52 | 5 | 6 |
